# Indoline-5-Sulfonamides: A Role of the Core in Inhibition of Cancer-Related Carbonic Anhydrases, Antiproliferative Activity and Circumventing of Multidrug Resistance

**DOI:** 10.3390/ph15121453

**Published:** 2022-11-23

**Authors:** Stepan K. Krymov, Alexander M. Scherbakov, Lyubov G. Dezhenkova, Diana I. Salnikova, Svetlana E. Solov’eva, Danila V. Sorokin, Daniela Vullo, Viviana De Luca, Clemente Capasso, Claudiu T. Supuran, Andrey E. Shchekotikhin

**Affiliations:** 1 Gause Institute of New Antibiotics, 11 B. Pirogovskaya Street, 119021 Moscow, Russia; 2 Department of Experimental Tumor Biology, Blokhin N.N. National Medical Research Center of Oncology, 115522 Moscow, Russia; 3 Department of NEUROFARBA, Section of Pharmaceutical and Nutraceutical Sciences, University of Florence, 50122 Florence, Italy; 4 Institute of Biosciences and Bioresources, CNR, Via Pietro Castellino 111, 80131 Napoli, Italy

**Keywords:** indoline-5-sulfonamide, carbonic anhydrase IX, antiproliferative activity, hypoxia, breast cancer, skin cancer, multidrug resistance, carbonic anhydrase XII, P-glycoprotein

## Abstract

The overexpression and activity of carbonic anhydrase (CA, EC 4.2.1.1) isoforms CA IX and CA XII promote the accumulation of exceeding protons and acidosis in the extracellular tumor environment. Sulfonamides are effective inhibitors of most families of CAs. In this study, using scaffold-hopping, indoline-5-sulfonamide analogs **4a–u** of the CA IX-selective inhibitor **3** were designed and synthesized to evaluate their biological properties. 1-Acylated indoline-5-sulfonamides demonstrated inhibitory activity against tumor-associated CA IX and XII with K_I_ values up to 132.8 nM and 41.3 nM. Compound **4f,** as one of the most potent inhibitors of CA IX and XII, exhibits hypoxic selectivity, suppressing the growth of MCF7 cells at 12.9 µM, and causes partial inhibition of hypoxia-induced CA IX expression in A431 skin cancer cells. **4e** and **4f** reverse chemoresistance to doxorubicin of K562/4 with overexpression of P-gp.

## 1. Introduction

One of the factors of the reduced effectiveness of chemo- and radiotherapy is hypoxic regions of tumors [1,2,3]. The deficient oxygen supply leads to the activation of transcriptional factor HIF-1α in tumor cells. In its turn, HIF-1α conceives expression of many proteins that advantage the development of aggressive phenotype of cancer cells [4]. One of the features of hypoxic cancer cells is the acidic extracellular pH (pHe) [5]. Increased level of protons causes resistance to the widely applied chemotherapeutic agents, including doxorubicin (Dox) [6] and cisplatin [7]. Aside from resistance development, acidic pHe destructs extracellular matrix and declines cell adhesion resulting in invasion and metastasis of tumors [8,9].

The acidic extracellular tumor environment arises from the coordinated work of transmembrane carbonic anhydrase (CA) IX and CA XII with sodium-hydrogen exchanger-1 [10]. CA IX and CA XII genes have a hypoxia response element that indicates their activation under the control of HIF-1α [11]. CA IX and CA XII are among 13 zinc metalloproteins of the α-carbonic anhydrases family with enzymatic activity [12]. Both enzymes are anchored in the cell membrane with active sites located in the extracellular milieu [13]. Utilizing the Zn^2+^ ion and His residues, CA IX and CA XII catalyze the hydration of CO_2_ to HCO_3_^−^ and H^+^ [14]. CA IX presence in normal tissue is limited to GI mucosa, whereas its expression in tumor cells is widespread and found in highly aggressive types of cancer, including breast, lung, brain, colon, rectum and kidney tumors [12,15]. Numerous studies have shown that the expression of CA IX is the prediction of a poor outcome [16,17,18,19]. However, CA XII, along with expression in several types of tumors, was also found in breast and colon normal tissues [20,21] and can correlate with a positive [22] as well as a negative prognosis [23,24]. Despite such uncertainty, several studies demonstrated the significance of CA XII in cancer severity [25,26]. For example, CA XII expression was increased in chemoresistant cells and influenced P-glycoprotein (P-gp) activity [27]. The development of CA XII as a pharmacological target for cancer therapy has led to successful reports of selective CA XII inhibitors overcoming multidrug resistance (MDR) in vivo [28,29].

**Figure 1:**
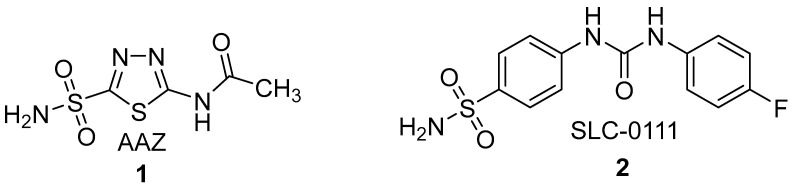
Chemical structures of clinically used diuretic AAZ (**1**) and CA IX inhibitor under clinical trial on diarylcarbamide scaffold (**2**).

Coordinating with the Zn^2+^ ion, sulfonamides such as acetazolamide (**1**) and SLC-0111 (**2**) (Figure 1) are the most studied inhibitors of the CA enzyme family [30,31,32,33,34,35,36,37,38]. SLC-0111 is the selective CA IX inhibitor; it reduces acidic pH, leading to the tumor and metastasis regression in preclinical models [39,40,41]. Combinations of different chemotherapeutic agents with SLC-0111 demonstrate a high potential for CA IX inhibition to treat aggressive and resistant types of cancer [42,43,44]. For example, the combination of the SLC-0111 with gemcitabine increased survival and enhanced tumor cell death of highly hypoxic and resistant pancreatic ductal adenocarcinomas in vivo [45].

**Figure 2:**
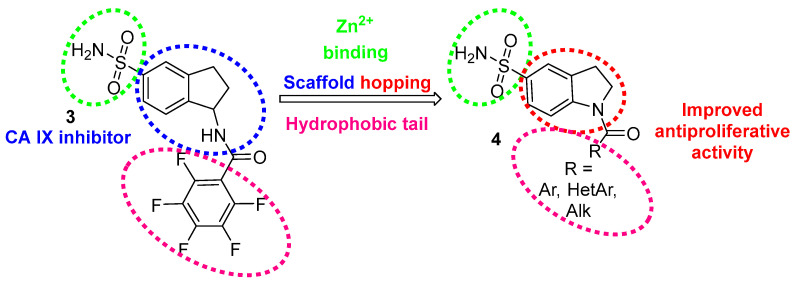
Scaffold hopping approach for the design of novel CA inhibitors based on indoline-5-sulfonamide core **4**.

Different groups have reported successful approaches to inhibit cancer-related CA IX and CA XII by indole-based sulfonamides [46,47,48]. Earlier, we studied 1-substituted isatin-5-sulfonamides as new CAs inhibitors, which suppressed the growth of tumor cells at low micromolar concentrations [49]. At this stage, we continue the work on modifying the structure of 5-indanesulfonamide derivative **3**, which selectively inhibits CA IX [50] and enhances the therapeutic effect of tumor irradiation in vivo [51]. However, 1-aminoindane-5-sulfonamide **3** has a limited antiproliferative effect on tumor cells [49], so the development of carbonic anhydrase inhibitors with improved anticancer potencies is a pivotal task. Scaffold hopping is an effective method to discover novel biologically active compounds by central core modification of the known lead compound [52,53,54,55,56]. Based on the selectivity of **3** toward CA IX and the known antiproliferative effect of indoline derivatives [57,58,59], we applied the scaffold hopping approach to replace 1-aminoindane to indoline core **4**, varying the structure of hydrophobic tail for the best binding to the targets (Figure 2).

**Figure 3:**
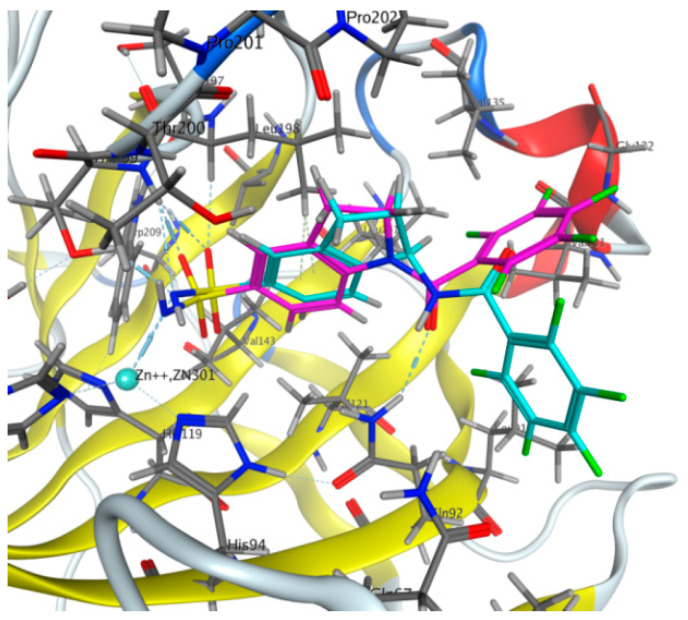
Modelling of aminoindanesulfoamide **3** (blue) and indoline analog **4e** (purple) in the active site of CA IX (PDB 5sz5).

To examine the results of the scaffold hopping for the discovery of new carbonic anhydrase inhibitors, we docked indane-5-sulfonamide **3** with indoline-5-sulfonamide analog **4** in the active site of CA IX (Figure 3). According to the modeling, **3** and perfluoro benzoyl derivative **4** demonstrate a similar orientation in the active site of CA IX. Both ligands interact with Zn^2+^ ions as sulfonamidate anions and form identical hydrogen bonds with Thr 199 and arene-H interactions with Leu 198 residues. The distinction of indoline ligand **4** from indane **3** lies in the orientation of the perfluorobenzoyl group that allows indoline inhibitor **4** to form novel interactions with the amino acid residue of CA IX. Thus, the additional hydrogen bond between the amides group of indoline ligand **4** and Gln 92 and the similar binding mode of central cores point to a promising potential of 1-acylindoline-5-sulfonamides **4** as new CA IX inhibitors.

## 2. Results

### 2.1. Chemistry

**Scheme 1:**
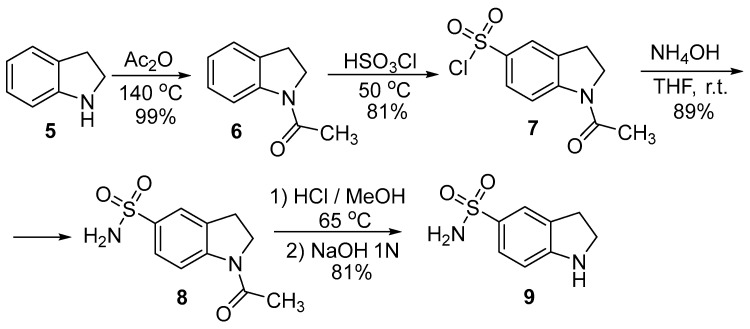
The synthesis of indoline-5-sulfonamide **9**.

Indolines exhibit chemical properties close to N-substituted anilines. Developed methods of indole reduction and reverse dehydrogenation open the convenient synthetic way to 5-substituted indoles. In particular, Terent’ev and Preobrazhenskaya applied this method to obtain indole-5-sulfonamide [60]. Firstly, to carry out an electrophilic substitution, indoline (**5**) was protected with acetic anhydride in quantitative yield (Scheme 1). Next, 1-acetylindoline (**6)** was treated with chlorosulfuric acid, which led to 1-acetylindloine-5-sulfochloride (**7**) in a good yield (81%). The following interaction of sulfochloride **7** with ammonia in THF resulted in the formation of 1-acetylindoline-5-sulfonamide (**8**) with an 89% yield. In the next step, hydrolysis of indoline **8** by hydrochloric acid gave indoline-5-sulfonamide (**9**) with 81% yield.

**Scheme 2:**
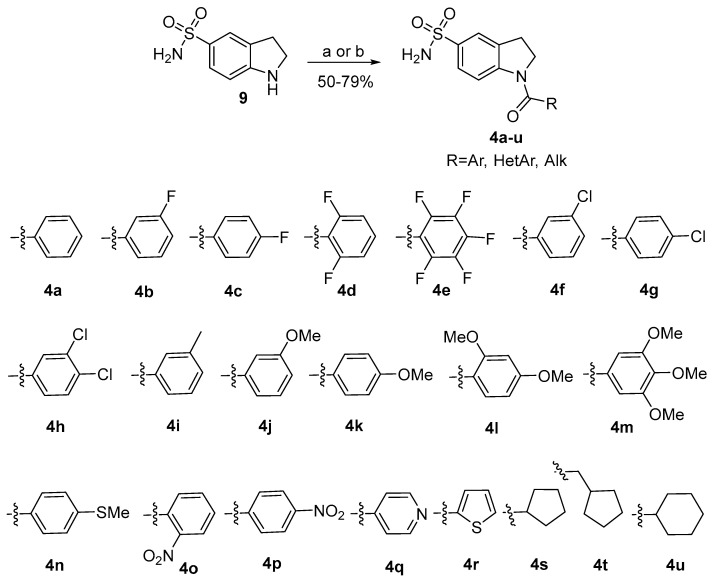
The synthesis of 1-acylindoline-5-sulfonamides **4a**–**4u**. Reagents and conditions: (a) acyl chloride, Py, CHCl_3_; r.t., (b) CDI, DMAP, THF, 40 °C.

To synthesize a library of 1-acylindoline-5-sulfonamides **4**, indoline-5-sulfonamide **9** was acylated by a series of acyl chlorides in the presence of pyridine in CHCl_3_ (Scheme 2). For the synthesis of isonicotinic derivative **4q**, the corresponding acid was activated by CDI and then treated with indoline-5-sulfonamide **9** in the presence of DMAP in THF. Applying these procedures, a library of 21 1-acylindoline-5-sulfonamides **4a**–**4u** was synthesized with 50–79% yields.

**Scheme 3:**
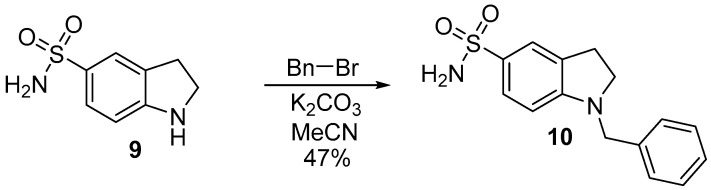
The synthesis of 1-benzylindoline-5-sulfonamide **10**.

To extend structure-activity relationships data, indoline-5-sulfonamide **9** was alkylated by benzyl bromide in MeCN in the presence of K_2_CO_3_ that yielded 1-benzyl-5-sulfonamide **10** (Scheme 3).

### 2.2. Carbonic Anhydrase Inhibition Assay

Synthesized indoline-5-sulfonamide derivatives **4a**–**4u**, **10** were evaluated against hCA I, hCA II, hCA IX and hCA XII by a stopped-flow technique (Table 1).

CA I and CA II are cytosolic proteins, that are widely expressed in erythrocytes, the eye, the GI tract, osteoclasts, and kidney cells [61]. CA II inhibitors are used in the clinical setting as diuretics and for glaucoma-related intraocular hypertension [62]. At the same time, CA I and CA II are considered the main off-target isoforms for the development of cancer-related CA IX and CA XII inhibitors [63].

The majority of indoline-5-sulfonamides **4a**–**4u**, **10** inhibits CA I at concentrations less than 100 nM. Structure-activity relationship points out that derivatives of benzoic acids with chlorine atoms **4f**, **g**, **h** demonstrate lower affinity toward this off-target isoform. At the same time, replacement of the phenyl **4a** by pyridine **4q**, thiophene **4r** or cycloalkanes **4s**, **t**, **u** does not affect K_I_ notably.

Indolines **4a4a–uu** inhibit CA II at much lower concentrations despite its structural similarity to indane derivative **3**. However, a group of indoline-5-sulfonamides **4f**–**4i**, **4p** has somewhat higher K_I_ at 31–66 nM. Interestingly, previously studied 6-chloro-5-sulfamylindolines as potential furosemide analogs did not show notable diuretic activity in vivo [64].

3-Chlorophenyl **4f**, thiophene **4r** and cyclopentyl **4s**, **t** derivatives are the most potent derivatives inhibiting CA IX around 100 nM. These compounds outperform a group of the least active derivatives by more than 100 times, which indicates the high importance of the structure of the lipophilic fragment for CA IX inhibition. Additionally, a comparison of indane **3** with indoline analog **4e** reveals that 1-aminoindane scaffold appears as a preferred scaffold toward CA IX isoform. Thereby, the affinity of 1-acylindoline-5-sulfonamides **4a4a–uu** for CA IX mainly depends on the structure of acyl moiety and is notably enhanced by 3-chlorophenyl and five-membered rings of aromatic and saturated nature.

1-Acylated indoline-5-sulfonamides **4d**, **g**, **h** are potent inhibitors of CA XII. Notably, for indoline-5-sulfonamides, the introduction of acyl group (**4a**), compared to less polar alkyl fragment (**10**), leads to a clear gain of activity against CA XII. The majority of 1-acylated indoline-5-sulfonamides inhibit CA XII around 100 nM. Additionally, 4-chloro **4g** and 3,4-dichloro derivatives **4h** demonstrate selectivity over CA I and CA IX isoforms and have similar K_I_ with CA II. Based on the CA inhibition profile, 3-chlorobenzoyl derivative **4f**, as one of the most potent compounds toward CA IX and CA XII, was selected as the lead compound for further investigation.

### 2.3. Docking Studies

**Figure 4:**
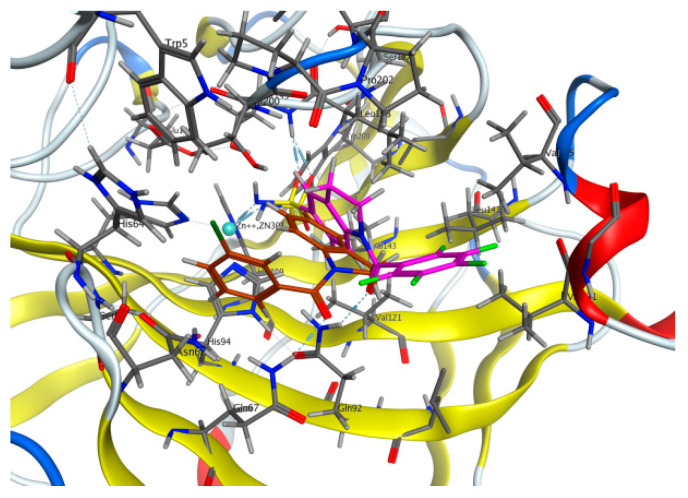
Docking of 3-chlorobenzoyl derivative **4f** (brown) and **4e** (purple) in the active site of CA IX (PDB 5sz5).

To investigate the difference of K_I_ against CA IX in 1-acylindoline-5-sulfonamides, we docked compound **4f** (Figure 4) in the active site of CA IX and compared it to a model of **4e**. As in the case with perfluoro derivative **4e**, **4f** displays coordination with Zn^2+^ ion as a sulfonamidate anion. Additionally, both ligands are acceptors of hydrogen bonds of Thr 199 residue. The differences between **4e** and **4f** occurred in interactions of indoline and acyl fragments. In particular, ligand **4f**, compared with **4e**, lacks arene-H interaction with Leu 198 but interacts with Gln 67 instead of Gln 92. Almost 10-fold higher activity of **4f** in vitro may be explained by the strength of interactions and physicochemical properties of compounds.

### 2.4. Antiproliferative Activity of Indoline-5-sulfonamides

A series of synthesized indoline-5-sulfonamides was tested on the MCF7 cancer cell line under normoxia and hypoxia using the MTT test. According to the results presented in Table 2, indoline-5-sulfonamides demonstrate a moderate antiproliferative effect. In general, indoline-5-sulfonamides do not lose their activity under hypoxia, which is the common reason for resistance to current antitumor agents. Comparing MTT results of **3** and indoline analog **4e**, we observed a clear gain of antiproliferative activity for the indoline derivative against the MCF7 line in normoxia and hypoxia. Again, indoline-5-sulfonamide **4f** as the most potent compound against MCF7 cells under hypoxia conditions (IC_50_ = 12.9 µM) demonstrates two-fold higher activity compared to normoxia conditions.

**Figure 5:**
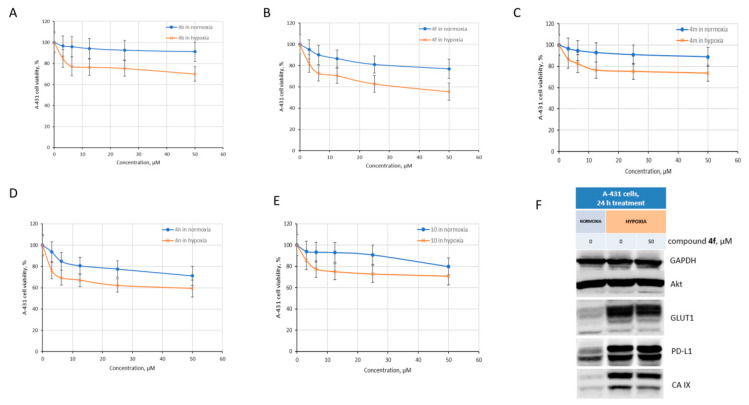
The activity of compounds **4b**, **f**, **m**, **n** and **10** against A431 skin cancer cells in normoxia and hypoxia. (**A**–**E**) antiproliferative evaluation; (**F**) immunoblotting with antibodies against Akt, GLUT1, PD-L1, CA IX; GAPDH antibodies were used as loading controls.

To evaluate the influence of CA inhibition on the antiproliferative activity, we selected five compounds **4b**, **f**, **m**, **n** and **10** with different inhibition profiles against CA IX for in-depth testing on cancer cells with increased CA IX expression. The database www.proteinatlas.org (accessed on 13 October 2022) contains data on gene expression in various cell lines. According to the data obtained from this database, A431 cells express CA IX at a very high level, which distinguishes them from human cell lines belonging to other tissues [https://www.proteinatlas.org/ENSG00000107159-CA9/cell+line (accessed on 13 October 2022)]. A431 cells are skin cancer cells, and their growth has already been intensely analyzed in hypoxia [65,66,67]. Hypoxia significantly alters the activity of signaling pathways in these cells; some of the revealed changes may be related to the induced activity of CAs. Moreover, Ren and colleagues described that hypoxia modulates cellular pathways in A431 cells, which are associated with radioresistance and enhanced migration [65]. Given the observations described above, we were expecting to reveal the high antiproliferative activity of the selected compounds against skin cancer cells. Surprisingly, the activity turned out to be at a moderate level. The cell growth curves are shown in Figure 5A–E. As can be seen in normoxia, the compounds inhibit cell growth by no more than 20%. Compounds **4b** and **4m** were the least active in normoxia; this finding may be associated with their low activity against CA IX (Table 1). In hypoxia, the activity of tested compounds was increased, but the 50% inhibition of growth was never achieved.

Compound **4f** was the most active under hypoxic conditions, inhibiting the growth of A431 cells by 44%. We wondered whether compound **4f** affected CA IX expression or whether its activity was limited to inhibiting the enzyme activity reported above (Table 1). To induce CA IX expression, cells were placed in hypoxia for 24 h, and then protein expression was analyzed by immunoblotting. Because the data on the expression of hypoxia-related proteins in the literature are highly inconsistent, we introduced additional controls. Antibodies to hypoxia-regulated and “non-hypoxic” proteins were used [68,69]. The expression of kinase Akt, which is not regulated directly by hypoxia/HIF-1α, was not altered under hypoxia conditions (Figure 5F). On the contrary, the expression of proteins (CA IX, PD-L1, GLUT1) associated with hypoxia pathways was significantly increased. Thus, these data again support a high expression of CA IX in A431 cells. In A431 cells treated with compound **4f**, a moderate decrease in CA IX expression was observed, whereas the expression of hypoxia-regulated proteins PD-L1 and GLUT1 did not change. Thus, lead compound **4f** not only blocks the enzyme activity of CA IX, but also causes partial inhibition of hypoxia-induced CA IX expression in skin cancer cells.

Next, we examined the activity of the whole series of indoline-5-sulfonamides against leukemia cell line K562. Surprisingly, among all tested compounds, only perfluoro derivative **4e** demonstrated significant activity and inhibited the growth of the cells at 10 µM. Other indoline derivatives showed low activity against K562 cells at 50 µM or the highest concentrations.

**Figure 6:**
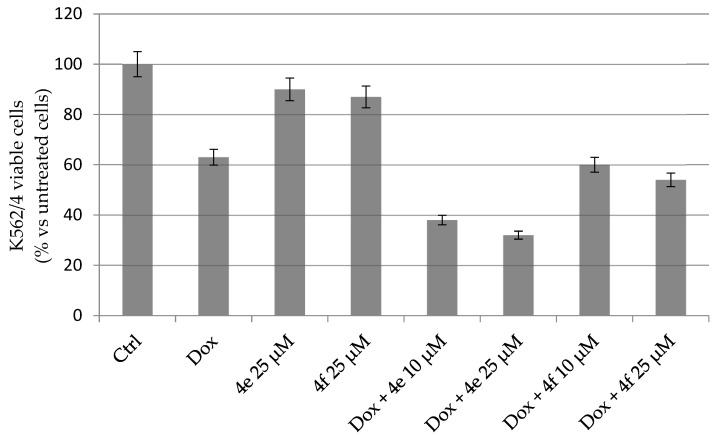
Susceptibility of resistant K562/4 line to combinations of Dox (25 µM) with indoline-5-sulfonamides **4e** and **4f**. Data are presented as means ± SD (*n* = 3).

Earlier, Kopecka and colleagues have shown that CA XII can interact with P-gp and influence its activity [27]. Subsequently, silencing or inhibition of CA XII by chemical agents may restore the sensitivity to Dox of resistant K562 and other tumor cells [70,71]. Given the potent inhibition of **4f** against CA XII and the activity of **4e** against K562, we examined their ability to reverse the chemoresistance of K562/4 with P-gp to Dox. MTT-test results summarized in Figure 6 show that **4e** and **4f** have low cytotoxic activity against MDR subline K562/4 as monoagents. However, treatment of K562/4 cells with combinations of Dox with **4e** and **4f** led to the decline of viable K562/4 cells. Specifically, the combination of Dox with perfluoro derivative **4e** demonstrated a clear dose-dependent antiproliferative effect against K562/4 cells.

## 3. Discussion

Scaffold hopping makes it possible to reveal previously unknown data on the structure-activity relationships. It is especially important for the search for selective inhibitors of enzymes with off-target isoforms. In addition to a Zn^2+^ binding sulfonamide group, inhibitors of tumor-associated CA IX require a lipophilic fragment responsible for interaction with the hydrophobic side of the enzyme. Thus, in the structure of the CA IX-selective sulfonamide **3**, we replaced the 1-aminoindane with an indoline fragment and varied the hydrophobic tail to create new inhibitors of CA IX. As a result, we have obtained a broad series of 1-substituted indoline-5-sulfonamides exhibiting a broad spectrum of K_I_ against four CA isoforms. In general, replacement of 1-aminoindane scaffold with indoline led to a significant increase of activity against cytosolic CA I and CA II isoforms. Simultaneously, inhibition of CA IX and CA XII by 1-acylindoline-5-sulfonamides **4a**–**4u** can vary in the range of two orders. Among indoline-5-sulfonamides with a high affinity toward cancer-related isoforms, indoline **4f** demonstrated a decreased activity toward cytosolic CA I and CA II.

The antiproliferative activity of the indoline-5-sulfonamides **4a**–**4u** series indicated the good activity of potent CA IX and CA XII inhibitor **4f**, which exhibits hypoxic selectivity and inhibits the growth of MCF7 cells at 12.9 µM. At the same time, indoline analog **4e** outperforms indane analog **3** against the MCF7 line. MTT-test of chosen indoline-5-sulfonamides did not reveal a high antiproliferative activity against A431 cells with a high expression of CA IX. However, immunoblotting has shown that the lead **4f** not only inhibits CA IX, but also suppresses CA IX expression under hypoxic conditions in A431 skin cancer cells.

The good inhibitory activity of some indoline-5-sulfonamides against CA XII, a new target for overcoming MDR, made it compelling to study **4e** and **4f** ability to overcome the resistance of K562/4 cells with the expression of P-gp. Combinations of compounds **4e** and **4f** with Dox pointed to their ability to increase the suppression of resistant cells K562/4.

Overall, 1-acylated indoline-5-sulfonamides represent a new scaffold of nanomolar inhibitors and suppressors of tumor-associated CA IX and CA XII that points out their potential as adjuvant and MDR-overcoming agents (Supplementary Materials).

## 4. Materials and Methods

### 4.1. Synthesis

#### 4.1.1. Instruments and General Information

NMR spectra were recorded on a Varian VXR-400 instrument operated at 400 MHz (^1^H NMR) and 100 MHz (^13^C NMR). Chemical shifts were measured in DMSO-d6, using tetramethylsilane as an internal standard. Analytical TLC was performed on Silica Gel F254 plates (Merck), column chromatography with a SilicaGel Merck 60. Melting points were determined using a Buchi SMP-20 apparatus and were uncorrected. High-resolution mass spectra were recorded with electron spray ionization on a Bruker Daltonics microOTOF-QII instrument. HPLC was performed using a Shimadzu Class-VP V6.12SP1 system. IR spectra were recorded on a Nicolet iS10 Fourier transform IR spectrometer (Nicolet iS10 FT-IR, Madison, WI). All solvents, chemicals and reagents were obtained commercially and used without purification. The purity of final compounds **4a**–**4u**, **10** was ≥95% as determined by HPLC analysis.

Indoline-5-sulfonamide **9** was synthesized from indoline **5** according to a previously reported procedure [72].

#### 4.1.2. 1-Acetylindoline (**6**)

To a stirring acetic anhydride (15 mL, 0.16 mol) was added indoline (**5**, 4 g, 0.034 mol). The reaction mixture was refluxed for 10 min, cooled to room temperature and poured onto ice. The pinkish precipitate was filtered and washed with water to obtain 5.39 g (99%) of *N*-acetylindoline, m.p. = 102–104 °C (102–104 °C lit.).

#### 4.1.3. 1-Acetyl-5-(chlorosulfonyl)indoline (**7**)

To a stirring chlorosulfonic acid (15 mL, 0.225 mol) cooled in an ice bath was added *N*-acetylindoline (**6**, 5.4 g, 0.034 mol) portionwise at 0–5 °C. The resulting mixture was heated to 50 °C for 2 h. Upon completion, the reaction mixture was cooled and poured onto ice, and the precipitate was filtered and washed with cold water twice to obtain 7.0 g (81%) of 5-(chlorosulfonyl)-*N*-acetylindoline as a white solid, m.p. = 167–169 °C (167–169 °C lit.).

#### 4.1.4. 1-Acetylindoline-5-sulfonamide (**8**)

To a solution of 5-(chlorosulfonyl)-*N*-acetylindoline (**7**, 7.0 g, 0.027 mol) in THF (40 mL) was added NH_4_OH (10 mL, 0.098 mol, 18% solution) at room temperature. The reaction mixture was stirred for 1 h and then concentrated in vacuo. The residue was diluted with water and adjusted to a pH of 7–8 with 1 N aq. HCl solution to give 5.8 g (89%) of *N*-acetylindoline-5-sulfonamide as a white solid, m.p. = 223–225 °C (223–224 °C lit.).

#### 4.1.5. Indoline-5-sulfonamide (**9**)

To a suspension of *N*-acetylindoline-5-sulfonamide (**8**, 5.8 g, 0.024 mol) in MeOH (30 mL) was added concentrated HCl (10 mL, 0.1 mol). The mixture was refluxed for 2 h, cooled, and the solvent was removed in vacuo. The product was dissolved in water (40 mL), and the solution was adjusted to a pH of 7–8 with 1N aq. NaOH solution. The brown precipitate was filtered and purified by flash chromatography to obtain 3.9 g (81%) of pure indoline-5-sulfonamide. ^1^H NMR (400 MHz, DMSO-*d*_6_): *δ* 7.39 (s, 1H, Ar), 7.35 (dd, *J*^1^ = 8.3, *J^2^* = 1.6 Hz, 1H, Ar), 6.88 (s, 2H, NH_2_), 6.46 (d, *J* = 8.3 Hz, 1H, Ar), 6.19 (d, 1H, Ar), 3.49 (t, *J* = 8.4 Hz, 2H, CH_2_), 2.94 (t, *J* = 8.4 Hz, 2H, CH_2_). ^13^C NMR (100 MHz, DMSO-*d*_6_): *δ* 155.9, 131.6, 129.2, 126.8, 122.5, 106.5, 46.9, 28.8.

#### 4.1.6. 1-Benzoylindoline-5-sulfonamide (**4a**)

To a solution of indoline-5-sulfonamide (**9**, 80 mg, 0.4 mmol) and pyridine (66 µL, 0.8 mmol) in CHCl_3_ (3 mL) was added 56 µL (0.48 mmol) of benzoyl chloride in CHCl_3_ (2 mL) at 0–5 °C. The resulting mixture was stirred at room temperature for 2 h, and the solvent was removed in vacuo. The residue was diluted with water (7 mL) and adjusted to a pH of 4–5 with 1 N aq. HCl solution and filtrated. The crude product was crystallized from MeOH to get 96 mg (79%) of pure 1-benzoylindoline-5-sulfonamide **4a**. A white powder, mp > 250 °C. HPLC (LW = 300 nm, gradient B 30/70/30% (35 min)) t_R_ = 11.7 min, purity 99%. ^1^H NMR (400 MHz, DMSO-*d*_6_): *δ* 7.96 (br s, 1H, Ar), 7.69 (s, 1H, Ar), 7.64 (d, *J* = 6.4 Hz, 1H, Ar), 7.59 (d, *J* = 7.2 Hz, 2H, Ar), 7.54–7.45 (m, 3H, Ar), 7.27 (s, 2H, NH_2_), 4.04 (t, *J* = 8.2 Hz, 2H, CH_2_), 3.12 (t, *J* = 8.2 Hz, 2H, CH_2_). ^13^C NMR (100 MHz, DMSO-*d*_6_): *δ* 169.1, 145.9, 139.6, 136.9, 134.2, 130.9, 129.0 (2C), 127.4 (2C), 125.7, 123.0, 116.3, 51.3, 27.9. HRMS (ESI) (*m*/*z*) [M+H]^+^: calculated for C_15_H_15_N_2_O_3_S 303.0798, found 303.0811.

#### 4.1.7. 1-(3-Fluorobenzoyl)indoline-5-sulfonamide (**4b**)

This compound was prepared from **9** and 3-fluorobenzoyl chloride as described for **4a**. A white powder, yield 67%, mp > 250 °C. HPLC (LW = 300 nm, gradient B 30/70/30% (35 min)) t_R_ = 13.7 min, purity 99%. ^1^H NMR (400 MHz, DMSO-*d*_6_): *δ* 8.07 (br s, 1H, Ar), 7.69 (s, 1H, Ar), 7.65 (d, *J* = 7.3 Hz, 1H, Ar), 7.59–7.51 (m, 1H, Ar), 7.51–7.42 (m, 2H, Ar), 7.45 (m, 1H, Ar), 7.26 (s, 2H, NH_2_), 4.05 (t, *J* = 8.2 Hz, 2H, CH_2_), 3.14 (t, *J* = 8.2 Hz, 2H, CH_2_). ^13^C NMR (100 MHz, DMSO-*d*_6_): *δ* 167.5, 162.2 (d, *J* = 245.4 Hz, C-F), 145.6, 139.8, 139.1 (d, *J* = 6.9 Hz, C-F), 134.2, 131.3 (d, *J* = 7.7 Hz), 125.8, 123.6, 123.0, 117.7 (d, *J* = 20.7 Hz), 116.4, 114.5 (d, *J* = 22.2 Hz), 51.2, 27.9. HRMS (ESI) (*m*/*z*) [M+H]^+^: calculated for C_15_H_14_FN_2_O_3_S 321.0704, found 321.0679.

#### 4.1.8. 1-(4-Fluorobenzoyl)indoline-5-sulfonamide (**4c**)

This compound was prepared from **9** and 4-fluorobenzoyl chloride as described for **4a**. A white powder, yield 73%, mp > 250 °C. HPLC (LW = 300 nm, gradient B 30/70/30% (35 min)) t_R_ = 13.2 min, purity 100%. ^1^H NMR (400 MHz, DMSO-*d*_6_): *δ* 7.90 (br s, 1H, Ar), 7.74–7.61 (m, 4H, Ar), 7.32 (m, 2H, Ar), 7.27 (s, 2H, NH_2_), 4.06 (t, *J* = 8.2 Hz, 2H, CH_2_), 3.13 (t, *J* = 8.2 Hz, 2H, CH_2_). ^13^C NMR (100 MHz, DMSO-*d*_6_): δ 168.1, 163.5 (d, *J* = 247.7 Hz, C-F), 145.8, 139.6, 134.2, 133.4, 130.3 (d, *J* = 8.4 Hz, 2C), 125.7, 123.0, 116.4, 116.0 (d, *J* = 21.5 Hz, 2C), 51.3, 27.9. HRMS (ESI) (*m*/*z*) [M+H]^+^: calculated for C_15_H_14_FN_2_O_3_S 321.0704, found 321.0721.

#### 4.1.9. 1-(2,6-Difluorobenzoyl)indoline-5-sulfonamide (**4d**)

This compound was prepared from **9** and 2,6-difluorobenzoyl chloride as described for **4a**. A white powder, yield 72%, mp = 244–246 °C. HPLC (LW = 300 nm, gradient B 30/70/30% (35 min)) t_R_ = 13.5 min, purity 98%. ^1^H NMR (400 MHz, DMSO-*d*_6_): *δ* 8.24 (d, *J* = 8.6 Hz, 1H, Ar), 7.79–7.70 (m, 2H, Ar), 7.62 (m, 1H, Ar), 7.32 (s, 2H, NH_2_), 7.31–7.22 (m, 2H, Ar), 3.93 (t, *J* = 8.6 Hz, 2H, CH_2_), 3.21 (t, *J* = 8.6 Hz, 2H, CH_2_). ^13^C NMR (100 MHz, DMSO-*d*_6_): *δ* 159.6, 158.4 (d, *J* = 248.4 Hz, C-F), 158.3 (d, *J* = 248.4 Hz, C-F), 144.6, 140.7, 134.3, 133.2 (t, *J* = 9.2 Hz), 126.1, 123.2, 116.3, 114.6 (t, *J* = 23.0 Hz), 112.9 (d, *J* = 23.0 Hz, 2C), 49.5, 27.5. HRMS (ESI) (*m*/*z*) [M+H]^+^: calculated for C_15_H_13_F_2_N_2_O_3_S 339.0609, found 339.0609.

#### 4.1.10. 1-(Perfluorobenzoyl)indoline-5-sulfonamide (**4e**)

This compound was prepared from **9** and perfluorobenzoyl chloride as described for **4a**. A white powder, yield 72%, mp = 187–189 °C. HPLC (LW = 300 nm, gradient B 30/70/30% (35 min)) t_R_ = 20.9 min, purity 99%. ^1^H NMR (400 MHz, DMSO-*d*_6_): *δ* 8.19 (d, *J* = 8.6 Hz, 1H, Ar), 7.77–7.70 (m, 2H, Ar), 7.35 (s, 2H, NH_2_), 4.05 (t, *J* = 8.2 Hz, 2H, CH_2_), 3.13 (t, *J* = 8.2 Hz, 2H, CH_2_). ^13^C NMR (100 MHz, DMSO-*d*_6_): *δ* 156.3, 144.2, 141.6 (m, C-F, 2C), 141.2, 139.1 (m, C-F, 2C), 134.7, 126.1, 125.2, 123.3, 116.5, 111.6 (m, C-F), 49.6, 27.5. HRMS (ESI) (*m*/*z*) [M+H]^+^: calculated for C_15_H_10_F_5_N_2_O_3_S 393.0327, found 393.0332. IR *ν* max, (film) cm^−1^ 3373 m, 3283 m, 1654 s, 1597 m, 1552 w, 1529 m, 1486 s, 1434 m, 1419 w, 1394 s, 1342 s, 1321 w, 1303 w, 1283 w, 1252 w, 1225 w, 1183 m, 1149 m, 1137 w, 1107 m, 1070 s, 988 s, 936 m, 916 m, 891 w, 832 s, 813 w, 785 m, 702 m, 677 m.

#### 4.1.11. 1-(3-Chlorobenzoyl)indoline-5-sulfonamide (**4f**)

This compound was prepared from **9** and 3-chlorobenzoyl chloride as described for **4a**. A white powder, yield 75%, mp > 250 °C. HPLC (LW = 300 nm, gradient B 30/70/30% (35 min)) t_R_ = 17.0 min, purity 99%. ^1^H NMR (400 MHz, DMSO-*d*_6_): *δ* 8.25–7.90 (br s, 1H, Ar), 7.72–7.62 (m, 3H, Ar), 7.62–7.48 (m, 3H, Ar), 7.26 (s, 2H, NH_2_), 4.04 (t, *J* = 8.2 Hz, 2H, CH_2_), 3.13 (t, *J* = 8.2 Hz, 2H, CH_2_). ^13^C NMR (100 MHz, DMSO-*d*_6_): *δ* 167.4, 145.6, 139.8, 138.9, 134.2, 133.7, 131.0, 130.7, 127.3, 126.1, 125.8, 123.0, 116.4, 51.2, 27.9. HRMS (ESI) (*m*/*z*) [M+H]^+^: calculated for C_15_H_14_ClN_2_O_3_S 337.0408, found 337.0386. IR *ν* max, (film) cm^−1^ 3336 s, 3189 m, 3079 w, 1644 s, 1592 m, 1566 w,1539 m, 1482 s, 1433 s, 1394 s, 1333 s, 1315 s, 1257 m, 1194 s, 1169 m, 1152 s, 1111 w, 1079 s, 999 w, 912 s, 892 s, 869 s, 835 s, 802 s, 772 w, 741 w, 714 m, 683 w.

#### 4.1.12. 1-(4-Chlorobenzoyl)indoline-5-sulfonamide (**4g**)

This compound was prepared from **9** and 4-chlorobenzoyl chloride as described for **4a**. A white powder, yield 74%, mp > 250 °C. HPLC (LW = 300 nm, gradient B 30/70/30% (35 min)) t_R_ = 17.1 min, purity 99%. ^1^H NMR (400 MHz, DMSO-*d*_6_): *δ* 8.15–7.90 (br s, 1H, Ar), 7.69 (s, 1H, Ar), 7.68–7.61(m, 3H, Ar), 7.56 (d, *J* = 8.2 Hz, 2H, Ar), 7.26 (s, 2H, NH_2_), 4.05 (t, *J* = 8.2 Hz, 2H, CH_2_), 3.13 (t, *J* = 8.2 Hz, 2H, CH_2_).^13^C NMR (100 MHz, DMSO-*d*_6_): *δ* 168.0, 145.7, 139.7, 135.7 135.6, 134.2, 129.5 (2C), 129.1 (2C), 125.8, 123.0, 116.4, 51.2, 27.9. HRMS (ESI) (*m*/*z*) [M+H]^+^: calculated for C_15_H_14_ClN_2_O_3_S 337.0408, found 337.0409.

#### 4.1.13. 1-(3,4-Dichlorobenzoyl)indoline-5-sulfonamide (**4h**)

This compound was prepared from **9** and 3,4-dichlorobenzoyl chloride as described for **4a**. A white powder, yield 73%, mp > 250 °C. HPLC (LW = 300 nm, gradient B 30/70/30% (35 min)) t_R_ = 21.5 min, purity 97%. ^1^H NMR (400 MHz, DMSO-*d*_6_): *δ* 8.35–8.02 (br s, 1H, Ar), 7.90 (d, *J* = 1.4 Hz, 1H, Ar), 7.76 (d, *J* = 8.6 Hz, 1H, Ar), 7.69 (s, 1H, Ar), 7.68–7.63(m, 1H, Ar), 7.60 (dd, *J* = 8.6 Hz, *J* = 1.4 Hz, 1H, Ar),7.26 (s, 2H, NH_2_), 4.05 (t, *J* = 8.2 Hz, 2H, CH_2_), 3.13 (t, *J* = 8.2 Hz, 2H, CH_2_). ^13^C NMR (100 MHz, DMSO-*d*_6_): *δ* 166.5, 145.5, 139.9, 137.3, 134.2, 133.6, 131.9, 131.4, 129.6, 127.8, 125.8, 123.0, 116.5, 51.2, 27.9. HRMS (ESI) (*m*/*z*) [M+H]^+^: calculated for C_15_H_13_Cl_2_N_2_O_3_S 371.0018, found 371.0005.

#### 4.1.14. 1-(3-Methylbenzoyl)indoline-5-sulfonamide (**4i**)

This compound was prepared from **9** and 3-methylbenzoyl chloride as described for **4a**. A white powder, yield 69%, mp = 247–249 °C. HPLC (LW = 300 nm, gradient B 30/70/30% (35 min)) t_R_ = 15.3 min, purity 99%. ^1^H NMR (400 MHz, DMSO-*d*_6_): *δ* 8.05–7.83 (br s, 1H, Ar), 7.68 (s, 1H, Ar), 7.63 (d, *J* = 6.6 Hz, 1H, Ar), 7.43–7.30 (m, 4H, Ar), 7.24 (s, 2H, NH_2_), 4.04 (t, *J* = 8.6 Hz, 2H, CH_2_), 3.12 (t, *J* = 8.6 Hz, 2H, CH_2_). ^13^C NMR (100 MHz, DMSO-*d*_6_): *δ* 169.2, 145.9, 139.5, 138.4, 136.9, 134.1, 131.5, 128.9, 127.8, 125.7, 124.4, 123.0, 116.2, 51.2, 27.9, 21.4. HRMS (ESI) (*m*/*z*) [M+H]^+^: calculated for C_16_H_17_N_2_O_3_S 317.0954, found 317.0981.

#### 4.1.15. 1-(3-Methoxybenzoyl)indoline-5-sulfonamide (**4j**)

This compound was prepared from **9** and 3-methoxybenzoyl chloride as described for **4a**. A white powder, yield 62%, mp = 225–227 °C. HPLC (LW = 300 nm, gradient B 30/70/30% (35 min)) t_R_ = 13.1 min, purity 99%. ^1^H NMR (400 MHz, DMSO-*d*_6_): *δ* 7.97 (br s, 1H, Ar), 7.68 (s, 1H, Ar), 7.63 (d, *J* = 6.9 Hz, 1H, Ar), 7.40 (t, *J* = 7.8 Hz 1H, Ar), 7.25 (s, 2H, NH_2_), 7.16–7.11 (m, 2H, Ar), 7.08 (dd, *J* = 7.8, *J* = 2.4 Hz, 1H, Ar), 4.04 (t, *J* = 8.2 Hz, 2H, CH_2_), 3.78 (s, 3H, OMe), 3.12 (t, *J* = 8.2 Hz, 2H, CH_2_). ^13^C NMR (100 MHz, DMSO-*d*_6_): *δ* 168.7, 159.6, 145.8, 139.6, 138.3, 134.2, 130.3, 125.7, 123.0, 119.4, 116.5, 116.3, 112.7, 55.8, 51.2, 27.9. HRMS (ESI) (*m*/*z*) [M+H]^+^: calculated for C_16_H_17_N_2_O_4_S 333.0904, found 333.0907.

#### 4.1.16. 1-(4-Methoxybenzoyl)indoline-5-sulfonamide (**4k**)

This compound was prepared from **9** and 4-methoxybenzoyl chloride as described for **4a**. A white powder, yield 64%, mp = 240–242 °C. HPLC (LW = 300 nm, gradient B 30/70/30% (35 min)) t_R_ = 12.2 min, purity 99%. ^1^H NMR (400 MHz, DMSO-*d*_6_): *δ* 7.82–7.73 (br s, 1H, Ar), 7.68 (s, 1H, Ar), 7.65–7.56 (m, 3H, Ar), 7.24 (s, 2H, NH_2_), 7.02 (d, *J* = 8.2 Hz, 2H, Ar), 4.10 (t, *J* = 8.2 Hz, 2H, CH_2_), 3.81 (s, 3H, OMe), 3.12 (t, *J* = 8.2 Hz, 2H, CH_2_). ^13^C NMR (100 MHz, DMSO-*d*_6_): *δ* 168.8, 161.4, 146.2, 139.3, 134.0, 129.8 (2C), 128.8, 125.7, 122.9, 116.3, 114.2 (2C), 55.8, 51.5, 28.0. HRMS (ESI) (*m*/*z*) [M+H]^+^: calculated for C_16_H_17_N_2_O_4_S 333.0904, found 333.0882.

#### 4.1.17. 1-(2,4-Dimethoxybenzoyl)indoline-5-sulfonamide (**4l**)

This compound was prepared from **9** and 2,4-dimethoxybenzoyl chloride as described for **4a**. A white powder, yield 64%, mp = 249–251 °C. HPLC (LW = 300 nm, gradient B 30/70/30% (35 min)) t_R_ = 12.9 min, purity 97%. ^1^H NMR (400 MHz, DMSO-*d*_6_): *δ* 8.18 (br s, 1H, Ar), 7.66 (s, 2H, Ar), 7.24 (br s, 3H, NH_2_, Ar), 6.66 (s, 1H, Ar), 6.61 (d, *J* = 7.8 Hz, 1H, Ar), 3.81 (s, 8H, CH_2,_ 2OMe) 3.11 (t, *J* = 8.2 Hz, 2H, CH_2_). ^13^C NMR (100 MHz, DMSO-*d*_6_): *δ* 167.7, 162.2, 156.9, 145.7, 139.4, 134.0, 129.3, 125.9, 123.0, 119.5, 116.0, 106.0, 99.1, 56.2, 55.9, 49.5, 27.5. HRMS (ESI) (*m*/*z*) [M+H]^+^: calculated for C_17_H_19_N_2_O_5_S 363.1009, found 363.0993.

#### 4.1.18. 1-(3,4,5-Trimethoxybenzoyl)indoline-5-sulfonamide (**4m**)

This compound was prepared from **9** and 3,4,5-trimethoxybenzoyl chloride as described for **4a**. A white powder, yield 58%, mp = 248–250 °C. HPLC (LW = 300 nm, gradient B 30/70/30% (35 min)) t_R_ = 10.9 min, purity 99%. ^1^H NMR (400 MHz, DMSO-*d*_6_): *δ* 7.85 (br s, 1H, Ar), 7.68 (s, 1H, Ar), 7.64 (d, *J* = 8.3 Hz, 1H, Ar), 7.24 (s, 2H, NH_2_), 6.90 (s, 2H, Ar), 4.10 (t, *J* = 8.2 Hz, 2H, CH_2_), 3.79 (s, 6H, 2OMe), 3.71 (s, 3H, OMe), 3.13 (t, *J* = 8.2 Hz, 2H, CH_2_). ^13^C NMR (100 MHz, DMSO-*d*_6_): *δ* 168.7, 153.3 (2C), 145.9, 139.5 (2C), 134.1, 132.2, 125.7, 122.9, 116.3, 105.1 (2C), 60.6, 56.6 (2C), 51.3, 27.8. HRMS (ESI) (*m*/*z*) [M+H]^+^: calculated for C_18_H_21_N_2_O_6_S 393.1115, found 333.1106. IR *ν* max, (film) cm^−1^ 3336 s, 3189 m, 3079 w, 1644 s, 1592 m, 1566 w, 1539 w, 1482 s, 1433 m, 1394 s, 1333 s, 1315 s, 1257 m, 1194 s, 1169 m, 1152 s, 1111 w, 1079 s, 999 w, 912 s, 892 s, 869 s, 835 s, 802 s, 772 w, 741 w, 714 m, 683 w.

#### 4.1.19. 1-(4-(Methylthio)benzoyl)indoline-5-sulfonamide (**4n**)

This compound was prepared from **9** and 4-(methylthio)benzoyl chloride as described for **4a**. A white powder, yield 63%, mp = 245–247 °C. HPLC (LW = 300 nm, gradient B 30/70/30% (35 min)) t_R_ = 16.4 min, purity 99%. ^1^H NMR (400 MHz, DMSO-*d*_6_): *δ* 7.98–7.76 (br s, 1H, Ar), 7.68 (s, 1H, Ar), 7.63 (d, *J* = 7.8 Hz, 1H, Ar), 7.55 (d, *J* = 8.2 Hz, 2H, Ar), 7.33 (d, *J* = 8.2 Hz, 2H, Ar), 7.26 (s, 2H, NH_2_), 4.08 (t, *J* = 8.6 Hz, 2H, CH_2_), 3.13 (t, *J* = 8.6 Hz, 2H, CH_2_). ^13^C NMR (100 MHz, DMSO-*d*_6_): *δ* 168.6, 146.0, 142.3, 139.5, 134.1, 132.7, 128.3 (2C), 125.7, 125.5 (2C), 123.0, 116.3, 51.4, 28.0, 14.6. HRMS (ESI) (*m*/*z*) [M+H]^+^: calculated for C_16_H_17_N_2_O_3_S_2_ 349.0675, found 349.0642.

#### 4.1.20. 1-(2-Nitrobenzoyl)indoline-5-sulfonamide (**4o**)

This compound was prepared from **9** and 2-nitrobenzoyl chloride as described for **4a**. A white powder, yield 66%, mp = 238–240 °C. HPLC (LW = 300 nm, gradient B 30/70/30% (35 min)) t_R_ = 11.7 min, purity 100%. ^1^H NMR (400 MHz, DMSO-*d*_6_): *δ* 8.27 (d, *J* = 8.2 Hz, 1H, Ar), 8.20 (d, *J* = 8.2 Hz, 1H, Ar), 7.30 (s, 2H, NH_2_), 3.83 (t, *J* = 8.2 Hz, 2H, CH_2_), 3.17 (t, *J* = 8.2 Hz, 2H, CH_2_). ^13^C NMR (100 MHz, DMSO-*d*_6_): *δ* 165.6, 145.3, 144.9, 140.1, 135.9, 133.9, 132.7, 131.4, 128.6, 126.1, 125.3, 123.1, 116.1, 50.2, 27.7. HRMS (ESI) (*m*/*z*) [M+H]^+^: calculated for C_15_H_14_N_3_O_5_S 348.0649, found 348.0612.

#### 4.1.21. 1-(4-Nitrobenzoyl)indoline-5-sulfonamide (**4p**)

This compound was prepared from **9** and 4-nitrobenzoyl chloride as described for **4a**. A white powder, yield 64%, mp = 247–249 °C. HPLC (LW = 300 nm, gradient B 30/70/30% (35 min)) t_R_ = 14.2 min, purity 97%. ^1^H NMR (400 MHz, DMSO-*d*_6_): *δ* 8.33 (d, *J* = 8.2 Hz, 2H, Ar), 8.19 (br s, 1H, Ar), 7.88 (d, *J* = 8.2 Hz, 2H, Ar), 7.71 (s, 2H, Ar), 7.30 (s, 2H, NH_2_), 4.02 (t, *J* = 8.2 Hz, 2H, CH_2_), 3.15 (t, *J* = 8.2 Hz, 2H, CH_2_). ^13^C NMR (100 MHz, DMSO-*d*_6_): *δ* 167.2, 148.7, 145.4, 142.8, 140.1, 134.3, 128.9 (2C), 125.8, 124.3 (2C), 123.0, 116.7, 51.1, 28.0. HRMS (ESI) (*m*/*z*) [M+H]^+^: calculated for C_15_H_14_N_3_O_5_S 348.0649, found 348.0654. IR *ν* max, (film) cm^−1^ 3352 m, 3258 s, 1650 s, 1593 m, 1512 s, 1476 s, 1431 m, 1392 s, 1335 s, 1313 m, 1290 w, 1254 m, 1192 m, 1146 s, 1108 m, 1079 s, 1016 m, 921 w, 907 m, 885 w, 861 s, 831 s, 737 w, 722 w, 702 s.

#### 4.1.22. 1-Isonicotinoylindoline-5-sulfonamide (**4q**)

To a solution of isonicotinic acid (90 mg, 0.73 mmol) in THF (5 mL) was added CDI (119 mg, 0.73 mmol). After 30 min until the end of the release of bubbles, a solution of indoline-5-sulfonamide (**9**, 110 mg, 0.6 mmol) in THF (5 mL) was added dropwise. The resulting mixture was heated to 40 °C for 2 h, cooled, and the solvent was removed in vacuo. The residue was diluted with water (7 mL) and filtrated. The crude product was crystallized from EtOH to get 84 mg (50%) of pure 1-isonicotinoylindoline-5-sulfonamide. A white powder, mp > 250 °C. HPLC (LW = 300 nm, gradient B 30/70/30% (35 min)) t_R_ = 8.8 min, purity 96%. ^1^H NMR (400 MHz, DMSO-*d*_6_): *δ* 8.73 (d, *J* = 5.7 Hz, 2H, Ar), 8.25–8.11 (br s, 1H, Ar), 7.70 (s, 1H, Ar), 7.59 (d, *J* = 5.7 Hz, 2H, Ar), 7.27 (s, 2H, NH_2_), 4.01 (t, *J* = 8.2 Hz, 2H, CH_2_), 3.15 (t, *J* = 8.2 Hz, 2H, CH_2_). ^13^C NMR (100 MHz, DMSO-*d*_6_): *δ* 166.9, 150.7 (2C), 145.4, 144.1, 140.1, 134.2, 125.9, 123.0, 121.5 (2C), 116.6, 51.0, 28.0. HRMS (ESI) (*m*/*z*) [M+H]^+^: calculated for C_14_H_14_N_3_O_3_S 304.0750, found 304.0752. IR *ν* max, (film) cm^−1^ 3350 w, 3180 w, 3072 w, 1638 s, 1591 m, 1545 m, 1484 s, 1440 m, 1400 s, 1332 m, 1308 s, 1255 w, 1224 w, 1197 w, 1144 s,1110 w, 1086 m, 1073 m, 992 w, 938 m, 898 w, 882 w, 832 s, 757 m, 729 w.

#### 4.1.23. 1-(Thiophene-2-carbonyl)indoline-5-sulfonamide (**4r**)

This compound was prepared from **9** and thiophene-2-carbonyl chloride as described for **4a**. A white powder, yield 62%, mp > 250 °C. HPLC (LW = 300 nm, gradient B 30/70/30% (35 min)) t_R_ = 11.0 min, purity 99%. ^1^H NMR (400 MHz, DMSO-*d*_6_): *δ* 8.11 (d, *J* = 8.2 Hz, 1H, Ar), 7.90 (d, *J* = 4.7 Hz, 1H, Ar), 7.79 (d, *J* = 3.9 Hz, 1H, Ar), 7.70 (s, 1H, Ar), 7.67 (d, *J* = 8.2 Hz, 1H, Ar) 7.27 (s, 2H, NH_2_), 7.21 (t, *J* = 3.9 Hz, *J* = 4.7 Hz, 1H, Ar), 4.47 (t, *J* = 8.2 Hz, 2H, CH_2_), 3.25 (t, *J* = 8.2 Hz, 2H, CH_2_). ^13^C NMR (100 MHz, DMSO-*d*_6_): *δ* 161.6, 146.3, 139.7, 139.4, 133.8, 132.4, 131.2, 128.5, 125.8, 122.9, 116.9, 51.1, 28.3. HRMS (ESI) (*m*/*z*) [M+H]^+^: calculated for C_13_H_13_N_2_O_3_S_2_ 309.0362, found 309.0372.

#### 4.1.24. 1-(Cyclopentanecarbonyl)indoline-5-sulfonamide (**4s**)

This compound was prepared from **9** and cyclopentanecarbonyl chloride as described for **4a**. A white powder, yield 58%, mp = 233–235 °C. HPLC (LW = 300 nm, gradient B 30/70/30% (35 min)) t_R_ = 14.2 min, purity 99%. ^1^H NMR (400 MHz, DMSO-*d*_6_): *δ* 8.15 (d, *J* = 7.8 Hz, 1H, Ar), 7.64–7.57 (m, 2H, Ar), 7.19 (s, 2H, NH_2_), 4.20 (t, *J* = 8.6 Hz, 2H, CH_2_), 3.18 (t, *J* = 8.6 Hz, 2H, CH_2_), 3.07–2.96 (m, 1H, CH), 1.93–1.82 (m, 2H, CH_2_), 1.78–1.69 (m, 2H, CH_2_), 1.68–1.60 (m, 2H, CH_2_), 1.59–1.50 (m, 2H, CH_2_). ^13^C NMR (100 MHz, DMSO-*d*_6_): *δ* 175.3, 146.4, 138.8, 133.3, 125.9, 122.7, 115.8, 48.5, 43.7, 29.9(2C), 27.6, 26.2 (2C). HRMS (ESI) (*m*/*z*) [M+H]^+^: calculated for C_14_H_19_N_2_O_3_S 295.1111, found 295.1133.

#### 4.1.25. 1-(2-Cyclopentylacetyl)indoline-5-sulfonamide (**4t**)

This compound was prepared from **9** and 2-cyclopentylacetyl chloride as described for **4a**. A white powder, yield 60%, mp = 184–186 °C. HPLC (LW = 300 nm, gradient B 30/70/30% (35 min)) t_R_ = 18.5 min, purity 100%. ^1^H NMR (400 MHz, DMSO-*d*_6_): *δ* 8.14 (d, *J* = 8.2 Hz, 1H, Ar), 7.63–7.58 (m, 2H, Ar), 7.19 (s, 2H, NH_2_), 4.13 (t, *J* = 8.6 Hz, 2H, CH_2_), 3.16 (t, *J* = 8.6 Hz, 2H, CH_2_), 2.30–2.18 (m, 1H, CH), 1.86–1.74 (m, 2H, CH_2_), 1.65–1.53 (m, 2H, CH_2_), 1.52–1.43 (m, 2H, CH_2_). ^13^C NMR (100 MHz, DMSO-*d*_6_): *δ* 171.9, 146.2, 138.7, 133.1, 125.9, 122.8, 115.5, 48.4, 41.6, 35.7, 32.6 (2C), 27.5, 25.0 (2C). HRMS (ESI) (*m*/*z*) [M+H]^+^: calculated for C_15_H_21_N_2_O_3_S 309.1267, found 309.1263.

#### 4.1.26. 1-(Cyclohexanecarbonyl)indoline-5-sulfonamide (**4u**)

This compound was prepared from **9** and cyclohexanecarbonyl chloride as described for **4a**. A white powder, yield 61%, mp > 250 °C. HPLC (LW = 300 nm, gradient B 30/70/30% (35 min)) t_R_ = 17.3 min, purity 100%. ^1^H NMR (400 MHz, DMSO-*d*_6_): *δ* 8.15 (d, *J* = 7.6 Hz, 1H, Ar), 7.62 (s, 1H, Ar), 7.60 (d, *J* = 7.6 Hz, 1H, Ar), 7.19 (s, 2H, NH_2_), 4.21 (t, *J* = 8.2 Hz, 2H, CH_2_), 3.17 (t, *J* = 8.2 Hz, 2H, CH_2_), 2.61–2.50 (m, 1H, CH), 1.77 (m, 4H, 2CH_2_), 1.65 (d, *J* = 11.9 Hz, 1H, CH_2_), 1.45–1.24 (m, 4H, 2CH_2_), 1.23–1.13 (m, 1H, CH_2_). ^13^C NMR (100 MHz, DMSO-*d*_6_): *δ* 175.1, 146.3, 138.9, 133.3, 125.8, 122.7, 115.9, 48.4, 43.1, 29.1(2C), 27.6, 25.9, 25.5 (2C). HRMS (ESI) (*m*/*z*) [M+H]^+^: calculated for C_15_H_21_N_2_O_3_S 309.1267, found 309.1223.

#### 4.1.27. 1-Benzylindoline-5-sulfonamide (**10**)

To a suspension of 100 mg (0.5 mmol) of indoline-5-sulfonamide **9** in MeCN (5 mL) was added 209 mg (1.5 mmol) of K_2_CO_3_ and 72 µL (0.6 mmol) of benzyl bromide at room temperature. After completion of the reaction monitored by TLC, the mixture was diluted with water (20 mL), adjusted to pH 7–8 with 1N HCl and extracted with EtOAc twice. The combined organic phases were dried over anhydrous Na_2_SO_4_, filtered, and concentrated in vacuum. The residue was purified by flash column chromatography (EtOAc/Hexane 1:2) to afford 70 mg of 1-benzylindoline-5-sulfonamide **10**. A white powder, yield 47%, mp = 144–146 °C. HPLC (LW = 300 nm, gradient B 30/70/30% (35 min)) t_R_ = 20.6 min, purity 99%. ^1^H NMR (400 MHz, DMSO-*d*_6_): *δ* 7.45 (dd, *J* = 8.2, *J* = 1.5 Hz, 1H, Ar), 8.19 (d, *J* = 1.5 Hz, 1H, Ar), 7.36–7.23 (m, 5H, Ar), 6.95 (s, 2H, NH_2_), 6.60 (d, *J* = 8.2, 1H, Ar), 4.38 (s, 2H, CH_2_), 3.42 (t, *J* = 8.6 Hz, 2H, CH_2_), 2.96 (t, *J* = 8.6 Hz, 2H, CH_2_). ^13^C NMR (100 MHz, DMSO-*d*_6_): *δ* 154.9, 137.9, 131.9, 130.1, 129.0 (2C), 128.2 (2C), 127.6, 126.9, 122.5, 105.1, 52.5, 51.2, 27.9. HRMS (ESI) (*m*/*z*) [M+H]^+^: calculated for C_15_H_17_N_2_O_2_S 289.1005, found 289.0982.

### 4.2. CA Inhibitory Assay

The CO_2_ hydration activity of the four hCA isoforms was monitored using an Applied Photophysics stopped-flow instrument [73]. Phenol red (at a concentration of 0.2 mM) was used as an indicator, working at the absorbance maximum of 557 nm, with 10 mM HEPES (pH 7.4) as a buffer, and 20 mM NaClO_4_ (for maintaining constant the ionic strength), following the initial rates of the CA-catalyzed CO_2_ hydration reaction for a period of 10–100 s. To determine the kinetic parameters by Lineweaver-Burk plots and the inhibition constants, a concentration of CO_2_ between 1.7 to 17 mM was used. At least six measurements of the original 5–10% reaction were used to assess the initial velocity for each inhibitor. The uncatalyzed rates were determined and detracted from the total observed rates. Stock inhibitor solutions (10–100 mM) were prepared in distilled-deionized water, and dilutions up to 0.1 nM were done with the buffer test. Inhibitor and enzyme solutions were preincubated together for 15 min at room temperature prior to assay in order to allow for the formation of the E-I complex or for the eventual active site-mediated hydrolysis of the inhibitor. The inhibition constants were obtained by non-linear least-squares methods using PRISM 6 and the Cheng-Prusoff equation, as reported earlier [74,75,76], and represent the mean from at least three different determinations. All enzymes were recombinant proteins obtained in-house, and their concentration in the assay system was 4.5–12 nM.

### 4.3. Molecular Modelling Studies

Molecular modelling was performed using Molecular Operating Environment (MOE) version 2014.09; Chemical Computing Group Inc., 1010 Sherbrooke St. West, Suite #910, Montreal, QC, Canada, H3A 2R7, 2014. CA IX structure was read from a PDB file 5sz5. Structural issues were automatically corrected using the Structure Preparation application. The hydrogen bond network and charges were optimized. Tethered energy minimization was performed using an AMBER10:EHT force field. The binding pocket of the receptor was specified by proximity to the cocrystallized ligand atoms. Chosen compounds were prepared using the wash command, and then partial charges were calculated. Ligand’s energy minimization was done using an MMFF94x force field. Deprotonation of strong acids and protonation of strong bases were checked in the wash panel. Docking placement was done using the triangle matcher algorithm with the ‘rotate bonds’ option. The 1st scoring function was London dG, and the 2nd scoring function was GBVI/WSA dG. MOE-Dock performed 30 independent docking runs. Docked complexes were ranked based on the docking scores (S). Finally, predicted complexes were analyzed for molecular interactions using the MOE window.

### 4.4. Cells and Antiproliferative Evaluation

The MCF7 human breast, A431 human skin cancer cells and K562 (ATCC CCL-243) chronic myelogenous leukemia cells were obtained from the ATCC collection. The MDR subline K562/4 [77] (kind gift of Dr. Alexander Shtil, Blokhin N.N. National Medical Research Center of Oncology) was obtained by stepwise selection of K562 cells for survival under continuous exposure to Dox. This subline expresses the MDR1 gene and functional P-gp and is characterized by a high resistance index for Dox [78]. The MCF7 and A431 cells were cultured in standard 4.5 g/L glucose DMEM medium (Gibco) supplemented with 10% FCS (HyClone), 2 mM L-glutamine, 50 U/mL penicillin, 50 µg/mL streptomycin (PanEco), 100 µg/mL sodium pyruvate (Santa Cruz) at 37 °C, 5% CO_2_ and 80–85% humidity in NuAire incubator. Suspension myelogenous leukemia cells (K562, K562/4) were propagated in RPMI-1640 (PanEco) with 5% FCS (HyClone), 2 mM L-glutamine, 100 U/mL penicillin, and 100 µg/mL streptomycin at 37 °C, 5% CO_2_, and 80–85% humidity in Binder incubator. Cells in the logarithmic phase of growth were used in the experiments. The growth inhibitory activity of compounds was assessed by the MTT test based on the metabolism of the MTT reagent (3-[4,5-dimethylthiazol-2-yl]-2,5-diphenyltetrazolium bromide) (Applichem) in living cells, with modifications as described previously in [49]. Compounds at different concentrations in 100 μL of the appropriate medium were added, and the cells were grown for 72 h. The hypoxia (1% O_2_) conditions were simulated in Binder multigas incubator, as described in [49]. After incubation with compounds, the medium was removed, the MTT reagent that was dissolved in the medium was added to the final concentration of 0.2 mg/mL to each well, and the incubation was performed for 2 h. Then the cell supernatants were removed, and purple formazan crystals were dissolved in 100% DMSO (350 μL per well). Culture plates were gently shaken, and the absorbance was measured at 571 nm with a reference wavelength of 630 nm on a MultiScan reader (ThermoFisher, Waltham, MA, USA). The viability of the cells was expressed as a percentage of the control. Dose-response curves were analyzed by regression analysis using sigmoid curves (Log(concentration) vs. normalized absorbance).

### 4.5. Statistical Analysis

All results were reported as means ± SD. One-way analysis of variance was used for the analysis of data. Differences were defined as significant at *p* < 0.05. GraphPad Prism7 (GraphPad Software, San Diego, CA, USA) was used for the determination of the half-maximal inhibitory concentration (IC_50_) values.

### 4.6. Immunoblotting

A431 cells were seeded on 100 mm dishes (Corning, Corning, NY, USA), and after 24 h growth, the compound **4f** was added to a fresh medium. To prepare cell extracts, A431 cells were twice washed in phosphate buffer and incubated for 10 min on ice in the modified lysis buffer containing 50 mM Tris-HCl, pH 7.5, 0.5% Igepal CA-630, 150 mM NaCl, 1 mM EDTA, 1 mM DTT, 1 mM PMSF, 0.1 mM sodium orthovanadate and aprotinin, leupeptin and pepstatin (1 µg/mL each) as described earlier in the work [79]. The protein content was determined by the Bradford method.

A431 cell lysates were separated in 10% SDS-PAGE under reducing conditions, transferred to a nitrocellulose membrane (GE HealthCare, Chicago, IL, USA), and processed according to a standard protocol. Akt, GLUT1, PD-L1, and CA IX antibodies were obtained from Cell Signaling Technology (Danvers, MA, USA); the antibodies against GAPDH (Cell Signaling Technology) were added to standardize loading. Goat anti-rabbit IgGs (Jackson ImmunoResearch, West Grove, PA, USA) conjugated to horseradish peroxidase were used as secondary antibodies. Signals were detected using the ECL reagent as described in Mruk and Cheng’s protocol [80] and an ImageQuant LAS4000 system (GE HealthCare).

## Figures and Tables

**Table 1:** Inhibition of hCAs I, II, IX and XII with indoline-5-sulfonamides **4a**–**4u**, **10** and AAZ and **3** as standards.

	K_I_ (nM) *			
Cmp	hCA I	hCA II	hCA IX	hCA XII
**4a**	79.0	5.4	>10^4^	258.5
**4b**	65.9	5.6	>10^4^	>10^4^
**4c**	88.3	9.5	2246.9	>10^4^
**4d**	268.1	9.1	1330.2	41.3
**4e**	52.2	3.4	1297.5	126.7
**4f**	303.9	31.3	141.2	111.7
**4g**	242.1	66.2	>10^4^	57.0
**4h**	350.1	54.0	>10^4^	56.0
**4i**	220.1	36.1	>10^4^	93.9
**4j**	67.7	9.2	>10^4^	110.0
**4k**	77.7	9.3	173.0	>10^4^
**4l**	71.5	4.0	203.2	112.5
**4m**	49.0	3.0	>10^4^	103.0
**4n**	71.4	9.3	1990.1	91.0
**4o**	83.4	4.5	>10^4^	119.6
**4p**	60.5	42.2	2109.4	198.1
**4q**	64.0	12.5	2394.3	>10^4^
**4r**	42.2	5.0	176.7	147.3
**4s**	60.9	7.1	132.8	88.1
**4t**	51.6	3.5	176.8	>10^4^
**4u**	67.0	1.8	222.6	>10^4^
**10**	41.0	3.0	>10^4^	>10^4^
**AAZ**	250	12.1	25.7	5.7
3 **	770	490	3.5	N.T.

* Mean from three different assays, by a stopped-flow technique (errors were in the range of ± 5–10% of the reported values); ** K_I_ data for compound **3** taken from previous research [50]; N.T.—not tested.

**Table 2:** Antiproliferative activity of indoline-5-sulfonamides **4a**–**4u**, **10** (the MTT assay after 72 h exposure; IC_50_, µM).

Compound	IC_50_^a^ against MCF7 Cells, µM	
	Normoxia	Hypoxia
**4a**	>50	36.9 ± 3.5
**4b**	>50	>50
**4c**	40.6 ± 3.8	32.7 ± 2.8
**4d**	>50	35.9 ± 3.4
**4e**	29.9 ± 2.7	20.2 ± 2.0
**4f**	24.5 ± 1.9	12.9 ± 1.2
**4g**	36.5 ± 3.3	24.2 ± 2.1
**4h**	35.2 ± 3.5	48.7 ± 4.5
**4i**	>50	43.6 ± 4.0
**4j**	46.2 ± 3.6	>50
**4k**	>50	>50
**4l**	37.0 ± 3.0	>50
**4m**	29.6 ± 2.2	36.7 ± 3.2
**4n**	18.4 ± 1.6	>50
**4o**	43.7 ± 3.7	39.5 ± 3.4
**4p**	48.8 ± 4.4	39.9 ± 3.5
**4q**	>50	>50
**4r**	47.7 ± 3.8	39.1 ± 3.9
**4s**	>50	>50
**4t**	38.0 ± 3.5	36.0 ± 3.3
**4u**	37.1 ± 3.5	39.2 ± 3.4
**10**	23.6 ± 2.0	34.8 ± 3.2
**3**	>50	>50

^a^ IC_50_ values represent an average of at least three independent experiments.

## Data Availability

Data is contained within the article.

## Supplementary Materials

The supporting information with ^1^H, ^13^C, IR spectra and HPLC analysis for indoline-5-sulfonamides can be downloaded at: https://www.mdpi.com/article/10.3390/ph15121453/s1.

## References

[1] Liang Y., Zheng T., Song R., Wang J., Yin D., Wang L., Liu H., Tian L., Fang X., Meng X. (2013). Hypoxia-mediated sorafenib resistance can be overcome by EF24 through Von Hippel-Lindau tumor suppressor-dependent HIF-1α inhibition in hepatocellular carcinoma. Hepatology.

[2] Xu K., Zhan Y., Yuan Z., Qiu Y., Wang H., Fan G., Wang J., Li W., Cao Y., Shen X. (2019). Hypoxia Induces Drug Resistance in Colorectal Cancer through the HIF-1α/miR-338-5p/IL-6 Feedback Loop. Mol. Ther..

[3] Morotti M., Bridges E., Valli A., Choudhry H., Sheldon H., Wigfield S., Gray N., Zois C.E., Grimm F., Jones D. (2019). Hypoxia-induced switch in SNAT2/SLC38A2 regulation generates endocrine resistance in breast cancer. Proc. Natl. Acad. Sci. USA.

[4] Jing X., Yang F., Shao C., Wei K., Xie M., Shen H., Shu Y. (2019). Role of hypoxia in cancer therapy by regulating the tumor microenvironment. Mol. Cancer.

[5] Švastová E., Hulíková A., Rafajová M., Zat’Ovičová M., Gibadulinová A., Casini A., Cecchi A., Scozzafava A., Supuran C.T., Pastorek J. (2004). Hypoxia activates the capacity of tumor-associated carbonic anhydrase IX to acidify extracellular pH. FEBS Lett..

[6] Mellor H.R., Callaghan R. (2011). Accumulation and distribution of doxorubicin in tumour spheroids: The influence of acidity and expression of P-glycoprotein. Cancer Chemother. Pharmacol..

[7] Federici C., Petrucci F., Caimi S., Cesolini A., Logozzi M., Borghi M., D’Ilio S., Lugini L., Violante N., Azzarito T. (2014). Exosome release and low pH belong to a framework of resistance of human melanoma cells to cisplatin. PLoS ONE.

[8] Chiche J., Ilc K., Laferrière J., Trottier E., Dayan F., Mazure N.M., Brahimi-Horn M.C., Pouysségur J. (2008). Hypoxia-Inducible Carbonic Anhydrase IX and XII Promote Tumor Cell Growth by Counteracting Acidosis through the Regulation of the Intracellular pH. Cancer Res..

[9] Estrella V., Chen T., Lloyd M., Wojtkowiak J., Cornnell H.H., Ibrahim-Hashim A., Bailey K., Balagurunathan Y., Rothberg J.M., Sloane B.F. (2013). Acidity Generated by the Tumor Microenvironment Drives Local Invasion. Cancer Res..

[10] Becker H.M. (2020). Carbonic anhydrase IX and acid transport in cancer. Br. J. Cancer.

[11] Wykoff C.C., Beasley N.J., Watson P., Turner K.J., Pastorek J., Sibtain A., Wilson G., Turley H., Talks K.L., Maxwell P. (2000). Hypoxia-inducible expression of tumor-associated carbonic anhydrases. Cancer Res..

[12] Pastorekova S., Gillies R.J. (2019). The role of carbonic anhydrase IX in cancer development: Links to hypoxia, acidosis, and beyond. Cancer Metastasis Rev..

[13] Pastoreková S., Pastorek J. (2004). Cancer-related carbonic anhydrase isozymes and their inhibition. Carbonic Anhydrase.

[14] Angeli A., Carta F., Nocentini A., Winum J.-Y., Zalubovskis R., Akdemir A., Onnis V., Eldehna W., Capasso C., Simone G. (2020). Carbonic Anhydrase Inhibitors Targeting Metabolism and Tumor Microenvironment. Metabolites.

[15] Ivanov S., Liao S.-Y., Ivanova A., Danilkovitch-Miagkova A., Tarasova N., Weirich G., Merrill M.J., Proescholdt M.A., Oldfield E.H., Lee J. (2001). Expression of Hypoxia-Inducible Cell-Surface Transmembrane Carbonic Anhydrases in Human Cancer. Am. J. Pathol..

[16] Nordfors K., Haapasalo J., Korja M., Niemelä A., Laine J., Parkkila A.-K., Pastorekova S., Pastorek J., Waheed A., Sly W.S. (2010). The tumour-associated carbonic anhydrases CA II, CA IX and CA XII in a group of medulloblastomas and supratentorial primitive neuroectodermal tumours: An association of CA IX with poor prognosis. BMC Cancer.

[17] Trastour C., Benizri E., Ettore F., Ramaioli A., Chamorey E., Pouysségur J., Berra E. (2007). HIF-1α and CA IX staining in invasive breast carcinomas: Prognosis and treatment outcome. Int. J. Cancer.

[18] Driessen A., Landuyt W., Pastorekova S., Moons J., Goethals L., Haustermans K., Nafteux P., Penninckx F., Geboes K., Lerut T. (2006). Expression of Carbonic Anhydrase IX (CA IX), a Hypoxia-Related Protein, Rather Than Vascular-Endothelial Growth Factor (VEGF), a Pro-Angiogenic Factor, Correlates with an Extremely Poor Prognosis in Esophageal and Gastric Adenocarcinomas. Ann. Surg..

[19] Loncaster J.A., Harris A.L., Davidson S.E., Logue J.P., Hunter R.D., Wycoff C.C., Pastorek J., Ratcliffe P.J., Stratford I.J., West C.M. (2001). Carbonic anhydrase (CA IX) expression, a potential new intrinsic marker of hypoxia: Correlations with tumor oxygen measurements and prognosis in locally advanced carcinoma of the cervix. Cancer Res..

[20] Wykoff C.C., Beasley N., Watson P.H., Campo L., Chia S.K., English R., Pastorek J., Sly W.S., Ratcliffe P., Harris A.L. (2001). Expression of the Hypoxia-Inducible and Tumor-Associated Carbonic Anhydrases in Ductal Carcinoma in Situ of the Breast. Am. J. Pathol..

[21] Kivelä A., Parkkila S., Saarnio J., Karttunen T.J., Kivelä J., Parkkila A.-K., Waheed A., Sly W.S., Grubb J.H., Shah G. (2000). Expression of a Novel Transmembrane Carbonic Anhydrase Isozyme XII in Normal Human Gut and Colorectal Tumors. Am. J. Pathol..

[22] Watson P.H., Chia S.K., Wykoff C.C., Han C., Leek R.D., Sly W.S., Gatter K.C., Ratcliffe P., Harris A.L. (2003). Carbonic anhydrase XII is a marker of good prognosis in invasive breast carcinoma. Br. J. Cancer.

[23] Ochi F., Shiozaki A., Ichikawa D., Fujiwara H., Nakashima S., Takemoto K., Kosuga T., Konishi H., Komatsu S., Okamoto K. (2015). Carbonic Anhydrase XII as an Independent Prognostic Factor in Advanced Esophageal Squamous Cell Carcinoma. J. Cancer.

[24] Chien M.-H., Ying T.-H., Hsieh Y.-H., Lin C.-H., Shih C.-H., Wei L.-H., Yang S.-F. (2012). Tumor-associated carbonic anhydrase XII is linked to the growth of primary oral squamous cell carcinoma and its poor prognosis. Oral Oncol..

[25] Guerrini G., Criscuoli M., Filippi I., Naldini A., Carraro F. (2018). Inhibition of smoothened in breast cancer cells reduces CAXII expression and cell migration. J. Cell. Physiol..

[26] Hsieh M.-J., Chen K.-S., Chiou H.-L., Hsieh Y.-S. (2010). Carbonic anhydrase XII promotes invasion and migration ability of MDA-MB-231 breast cancer cells through the p38 MAPK signaling pathway. Eur. J. Cell Biol..

[27] Kopecka J., Campia I., Jacobs A., Frei A.P., Ghigo D., Wollscheid B., Riganti C. (2015). Carbonic anhydrase XII is a new therapeutic target to overcome chemoresistance in cancer cells. Oncotarget.

[28] Salaroglio I.C., Mujumdar P., Annovazzi L., Kopecka J., Mellai M., Schiffer D., Poulsen S.-A., Riganti C. (2018). Carbonic Anhydrase XII Inhibitors Overcome P-Glycoprotein–Mediated Resistance to Temozolomide in Glioblastoma. Mol. Cancer Ther..

[29] Mujumdar P., Kopecka J., Bua S., Supuran C.T., Riganti C., Poulsen S.-A. (2019). Carbonic Anhydrase XII Inhibitors Overcome Temozolomide Resistance in Glioblastoma. J. Med. Chem..

[30] Nocentini A., Angeli A., Carta F., Winum J.-Y., Zalubovskis R., Carradori S., Capasso C., Donald W.A., Supuran C.T. (2021). Reconsidering anion inhibitors in the general context of drug design studies of modulators of activity of the classical enzyme carbonic anhydrase. J. Enzym. Inhib. Med. Chem..

[31] Berrino E., Michelet B., Martin-Mingot A., Carta F., Supuran C.T., Thibaudeau S. (2021). Modulating the Efficacy of Carbonic Anhydrase Inhibitors through Fluorine Substitution. Angew. Chem..

[32] Supuran C.T. (2021). Emerging role of carbonic anhydrase inhibitors. Clin. Sci..

[33] McDonald P.C., Chafe S.C., Supuran C.T., Dedhar S. (2022). Cancer Therapeutic Targeting of Hypoxia Induced Carbonic Anhydrase IX: From Bench to Bedside. Cancers.

[34] Kumar A., Siwach K., Supuran C.T., Sharma P.K. (2022). A decade of tail-approach based design of selective as well as potent tumor associated carbonic anhydrase inhibitors. Bioorganic Chem..

[35] Tawfik H.O., Petreni A., Supuran C.T., El-Hamamsy M.H. (2022). Discovery of new carbonic anhydrase IX inhibitors as anticancer agents by toning the hydrophobic and hydrophilic rims of the active site to encounter the dual-tail approach. Eur. J. Med. Chem..

[36] Tawfik H.O., Belal A., Abourehab M.A.S., Angeli A., Bonardi A., Supuran C.T., El-Hamamsy M.H. (2022). Dependence on linkers’ flexibility designed for benzenesulfonamides targeting discovery of novel hCA IX inhibitors as potent anticancer agents. J. Enzyme Inhib. Med. Chem..

[37] Arrighi G., Puerta A., Petrini A., Hicke F.J., Nocentini A., Fernandes M.X., Padrón J.M., Supuran C.T., Fernández-Bolaños J.G., López Ó. (2022). Squaramide-Tethered Sulfonamides and Coumarins: Synthesis, Inhibition of Tumor-Associated CAs IX and XII and Docking Simulations. Int. J. Mol. Sci..

[38] Manzoor S., Angeli A., Zara S., Carradori S., Rahman A., Raza K., Supuran C.T., Hoda N. (2022). Development of benzene and benzothiazole-sulfonamide analogues as selective inhibitors of the tumor-associated carbonic anhydrase IX. Eur. J. Med. Chem..

[39] Lock F.E., McDonald P.C., Lou Y., Serrano I., Chafe S.C., Ostlund C., Aparicio S., Winum J.-Y., Supuran C.T., Dedhar S. (2013). Targeting carbonic anhydrase IX depletes breast cancer stem cells within the hypoxic niche. Oncogene.

[40] Pacchiano F., Carta F., McDonald P.C., Lou Y., Vullo D., Scozzafava A., Dedhar S., Supuran C.T. (2011). Ureido-Substituted Benzenesulfonamides Potently Inhibit Carbonic Anhydrase IX and Show Antimetastatic Activity in a Model of Breast Cancer Metastasis. J. Med. Chem..

[41] Lou Y., McDonald P.C., Oloumi A., Chia S., Ostlund C., Ahmadi A., Kyle A., Keller U.A.D., Leung S., Huntsman D. (2011). Targeting Tumor Hypoxia: Suppression of Breast Tumor Growth and Metastasis by Novel Carbonic Anhydrase IX Inhibitors. Cancer Res.

[42] Kalinin S., Malkova A., Sharonova T., Sharoyko V., Bunev A., Supuran C.T., Krasavin M. (2021). Carbonic Anhydrase IX Inhibitors as Candidates for Combination Therapy of Solid Tumors. Int. J. Mol. Sci..

[43] Boyd N.H., Walker K., Fried J., Hackney J.R., McDonald P.C., Benavides G.A., Spina R., Audia A., Scott S.E., Libby C.J. (2017). Addition of carbonic anhydrase 9 inhibitor SLC-0111 to temozolomide treatment delays glioblastoma growth in vivo. JCI Insight.

[44] Hedlund E.-M.E., McDonald P.C., Nemirovsky O., Awrey S., Jensen L.D., Dedhar S. (2019). Harnessing Induced Essentiality: Targeting Carbonic Anhydrase IX and Angiogenesis Reduces Lung Metastasis of Triple Negative Breast Cancer Xenografts. Cancers.

[45] McDonald P.C., Chafe S.C., Brown W.S., Saberi S., Swayampakula M., Venkateswaran G., Nemirovsky O., Gillespie J.A., Karasinska J.M., Kalloger S.E. (2019). Regulation of pH by Carbonic Anhydrase 9 Mediates Survival of Pancreatic Cancer Cells With Activated KRAS in Response to Hypoxia. Gastroenterology.

[46] Güzel-Akdemir Ö., Demir-Yazıcı K., Vullo D., Supuran C.T., Akdemir A. (2022). New Pyridinium Salt Derivatives of 2-(Hydrazinocarbonyl)-3-phenyl-1H-indole-5-sulfonamide as Selective Inhibitors of Tumour-Related Human Carbonic Anhydrase Isoforms IX and XII. Anti-Cancer Agents Med. Chem..

[47] Singh P., Goud N.S., Swain B., Sahoo S.K., Choli A., Angeli A., Kushwah B.S., Yaddanapudi V.M., Supuran C.T., Arifuddin M. (2022). Synthesis of a new series of quinoline/pyridine indole-3-sulfonamide hybrids as selective carbonic anhydrase IX inhibitors. Bioorganic Med. Chem. Lett..

[48] Kumar S., Rulhania S., Jaswal S., Monga V. (2020). Recent advances in the medicinal chemistry of carbonic anhydrase inhibitors. Eur. J. Med. Chem..

[49] Krymov S.K., Scherbakov A.M., Salnikova D.I., Sorokin D.V., Dezhenkova L.G., Ivanov I.V., Vullo D., De Luca V., Capasso C., Supuran C.T. (2021). Synthesis, biological evaluation, and in silico studies of potential activators of apoptosis and carbonic anhydrase inhibitors on isatin-5-sulfonamide scaffold. Eur. J. Med. Chem..

[50] Thiry A., Ledecq M., Cecchi A., Dogné J.-M., Wouters J., Supuran C.T., Masereel B. (2006). Indanesulfonamides as Carbonic Anhydrase Inhibitors. Toward Structure-Based Design of Selective Inhibitors of the Tumor-Associated Isozyme CA IX. J. Med. Chem..

[51] Dubois L., Peeters S., Lieuwes N.G., Geusens N., Thiry A., Wigfield S., Carta F., Mcintyre A., Scozzafava A., Dogné J.-M. (2011). Specific inhibition of carbonic anhydrase IX activity enhances the in vivo therapeutic effect of tumor irradiation. Radiother. Oncol..

[52] Langdon S.R., Ertl P., Brown N. (2010). Bioisosteric Replacement and Scaffold Hopping in Lead Generation and Optimization. Mol. Inform..

[53] Hu Y., Stumpfe D., Bajorath J. (2016). Recent Advances in Scaffold Hopping. J. Med. Chem..

[54] Böhm H.-J., Flohr A., Stahl M. (2004). Scaffold hopping. Drug Discov. Today: Technol..

[55] Dick A., Cocklin S. (2020). Bioisosteric Replacement as a Tool in Anti-HIV Drug Design. Pharmaceuticals.

[56] Alhadrami H.A., Sayed A.M., Al-Khatabi H., Alhakamy N.A., Rateb M.E. (2021). Scaffold Hopping of α-Rubromycin Enables Direct Access to FDA-Approved Cromoglicic Acid as a SARS-CoV-2 M^Pro^ Inhibitor. Pharmaceuticals.

[57] Wang S.-Y., Liu X., Meng L.-W., Li M.-M., Li Y.-R., Yu G.-X., Song J., Zhang H.-Y., Chen P., Zhang S.-Y. (2021). Discovery of indoline derivatives as anticancer agents via inhibition of tubulin polymerization. Bioorganic Med. Chem. Lett..

[58] Thakur A., Singh A., Kaur N., Ojha R., Nepali K. (2019). Steering the antitumor drug discovery campaign towards structurally diverse indolines. Bioorganic Chem..

[59] Fu D.-J., Li M., Zhang S.-Y., Li J.-F., Sha B., Wang L., Zhang Y.-B., Chen P., Hu T. (2019). Discovery of indoline derivatives that inhibit esophageal squamous cell carcinoma growth by Noxa mediated apoptosis. Bioorganic Chem..

[60] Preobrazhenskaya M.N. (1967). Synthesis of Substituted Indoles via Indolines. Russ. Chem. Rev..

[61] Supuran C.T. (2016). Structure and function of carbonic anhydrases. Biochem. J..

[62] Mishra C.B., Tiwari M., Supuran C.T. (2020). Progress in the development of human carbonic anhydrase inhibitors and their pharmacological applications: Where are we today?. Med. Res. Rev..

[63] Supuran C.T. (2021). Novel carbonic anhydrase inhibitors. Future Med. Chem..

[64] Glamkowski E.J., Reitano P.A. (1979). Synthesis and evaluation for diuretic activity of 1-substituted 6-chloro-5-sulfamylindolines. J. Med. Chem..

[65] Ren Y., Hao P., Dutta B., Cheow E.S.H., Sim K.H., Gan C.S., Lim S.K., Sze S.K. (2013). Hypoxia Modulates A431 Cellular Pathways Association to Tumor Radioresistance and Enhanced Migration Revealed by Comprehensive Proteomic and Functional Studies. Mol. Cell. Proteom..

[66] Ren Y., Hao P., Law S.K.A., Sze S.K. (2014). Hypoxia-induced Changes to Integrin α 3 Glycosylation Facilitate Invasion in Epidermoid Carcinoma Cell Line A431. Mol. Cell. Proteom..

[67] Misra A., Pandey C., Sze S.K., Thanabalu T. (2012). Hypoxia Activated EGFR Signaling Induces Epithelial to Mesenchymal Transition (EMT). PLoS ONE.

[68] Boström P., Thoms J., Sykes J., Ahmed O., Evans A., van Rhijn B.G., Mirtti T., Stakhovskyi O., Laato M., Margel D. (2016). Hypoxia Marker GLUT-1 (Glucose Transporter 1) is an Independent Prognostic Factor for Survival in Bladder Cancer Patients Treated with Radical Cystectomy. Bladder Cancer.

[69] Smith V., Mukherjee D., Lunj S., Choudhury A., Hoskin P., West C., Illidge T. (2021). The effect of hypoxia on PD-L1 expression in bladder cancer. BMC Cancer.

[70] Teodori E., Braconi L., Bua S., Lapucci A., Bartolucci G., Manetti D., Romanelli M.N., Dei S., Supuran C.T., Coronnello M. (2020). Dual P-Glycoprotein and CA XII Inhibitors: A New Strategy to Reverse the P-gp Mediated Multidrug Resistance (MDR) in Cancer Cells. Molecules.

[71] Kopecka J., Rankin G.M., Salaroglio I.C., Poulsen S.-A., Riganti C. (2016). P-glycoprotein-mediated chemoresistance is reversed by carbonic anhydrase XII inhibitors. Oncotarget.

[72] Borror A.L., Chinoporos E., Filosa M.P., Herchen S.R., Petersen C.P., Stern C.A., Onan K.D. (1988). Regioselectivity of electrophilic aromatic substitution: Syntheses of 6- and 7-sulfamoylindolines and -indoles. J. Org. Chem..

[73] Khalifah R.G. (1971). The carbon dioxide hydration activity of carbonic anhydrase. I. Stop-flow kinetic studies on the native human isoenzymes B and C. J. Biol. Chem..

[74] Del Prete S., Vullo D., De Luca V., Carginale V., di Fonzo P., Osman S.M., AlOthman Z., Supuran C.T., Capasso C. (2016). Anion inhibition profiles of the complete domain of the η-carbonic anhydrase from Plasmodium falciparum. Bioorganic Med. Chem..

[75] Del Prete S., Vullo D., De Luca V., Carginale V., di Fonzo P., Osman S.M., AlOthman Z., Supuran C.T., Capasso C. (2016). Anion inhibition profiles of α-, β- and γ-carbonic anhydrases from the pathogenic bacterium Vibrio cholerae. Bioorganic Med. Chem..

[76] De Luca V., Vullo D., Del Prete S., Carginale V., Osman S.M., AlOthman Z., Supuran C.T., Capasso C. (2016). Cloning, characterization and anion inhibition studies of a γ-carbonic anhydrase from the Antarctic bacterium Colwellia psychrerythraea. Bioorganic Med. Chem..

[77] Sagnou M., Novikov F.N., Ivanova E.S., Alexiou P., Stroylov V.S., Titov I.Y., Tatarskiy V.V., Vagida M.S., Pelecanou M., Shtil A.A. (2020). Novel curcumin derivatives as P-glycoprotein inhibitors: Molecular modeling, synthesis and sensitization of multidrug resistant cells to doxorubicin. Eur. J. Med. Chem..

[78] Shchekotikhin A.E., Dezhenkova L.G., Susova O.Y., Glazunova V.A., Luzikov Y.N., Sinkevich Y.B., Buyanov V.N., Shtil A.A., Preobrazhenskaya M.N. (2007). Naphthoindole-based analogues of tryptophan and tryptamine: Synthesis and cytotoxic properties. Bioorganic Med. Chem..

[79] Scherbakov A.M., Lobanova Y.S., Shatskaya V.A., Onopchenko O.V., Gershtein E.S., Krasil’nikov M.A. (2006). Activation of mitogenic pathways and sensitization to estrogen-induced apoptosis: Two independent characteristics of tamoxifen-resistant breast cancer cells?. Breast Cancer Res. Treat..

[80] Mruk D.D., Cheng C.Y. (2011). Enhanced chemiluminescence (ECL) for routine immunoblotting: An inexpensive alternative to commercially available kits. Spermatogenesis.

