# Supplementary material for: Indoline-5-Sulfonamides: A Role of the Core in Inhibition of Cancer-Related Carbonic Anhydrases, Antiproliferative Activity and Circumventing of Multidrug Resistance
Source: Pharmaceuticals (Basel). 2022 Nov 23;15(12):1453. doi: 10.3390/ph15121453 (PMC9783868; doi:10.3390/ph15121453)
Supplement: Supplementary file 1 [file pharmaceuticals-15-01453-s001.zip › pharmaceuticals-2014909-supplementary.pdf]

1-benzoylindoline-5-sulfonamide **(4a)**

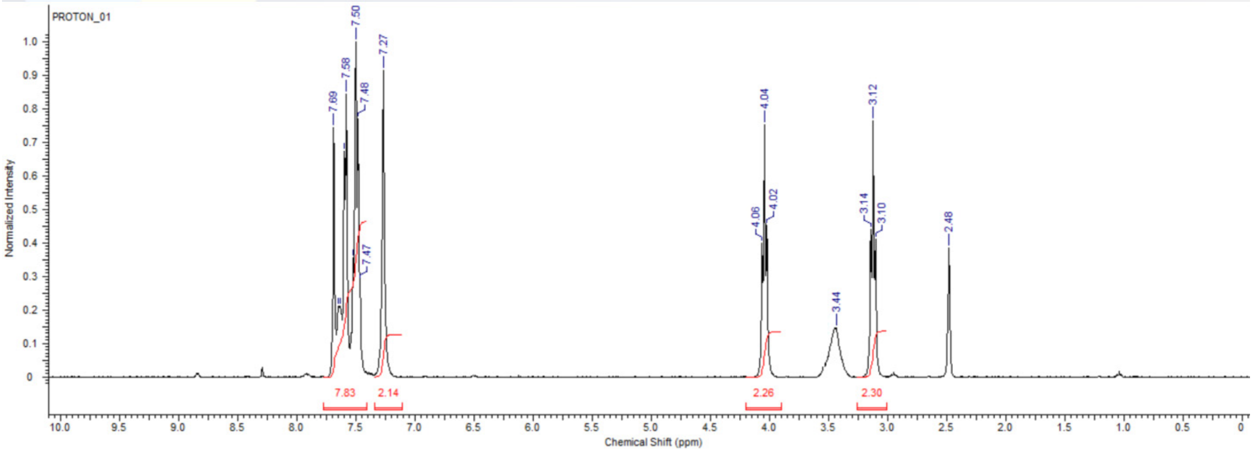

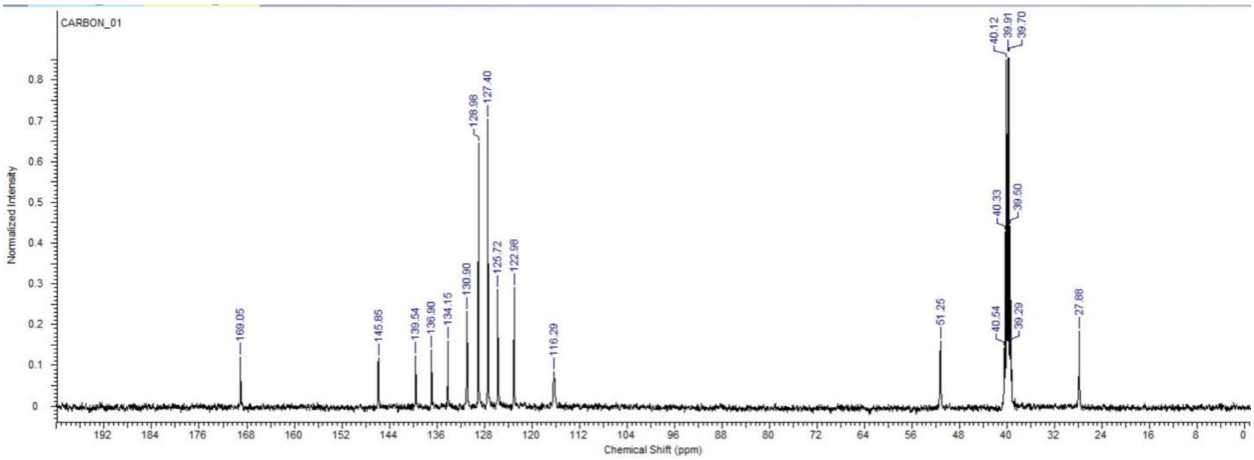

Chromatogram
ST-11 C:\LabSolutions\Data\CHEK\ST O1011-03.lcd

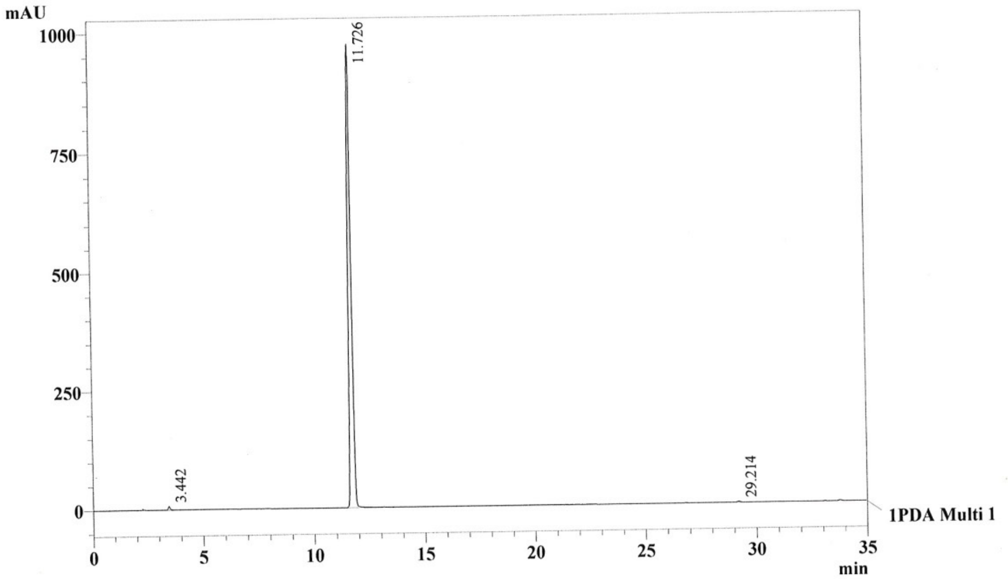

1 PDA Multi 1 / 300nm 4nm

PeakTable

PDA Ch1 300nm 4nm

Peak#	Ret. Time	Area	Height	Area %
1	3.442	43396	7426	0.472
2	11.726	9132228	973253	99.349
3	29.214	16412	1916	0.179
Total		9192035	982595	100.000

Method

<<LC Program>>

Time	Unit	Command	Value
0.10	Pumps	B.Conc	30
30.00	Pumps	B.Conc	70
33.00	Pumps	B.Conc	30
45.00	Controller	Stop	

Method Filename : FOS B.lcm

Shimadzu LC-20AD; 2-System FOS, Colon Kromasil 100-C18,.size 5mkm, 4,6*250mm, N 86912
Elution: A - H3PO4 0.01M pH 2.6; B - MeCN, fl. 1,0 ml/min, loop 20mkl.

1-(3-fluorobenzoyl)indoline-5-sulfonamide **(4b)**

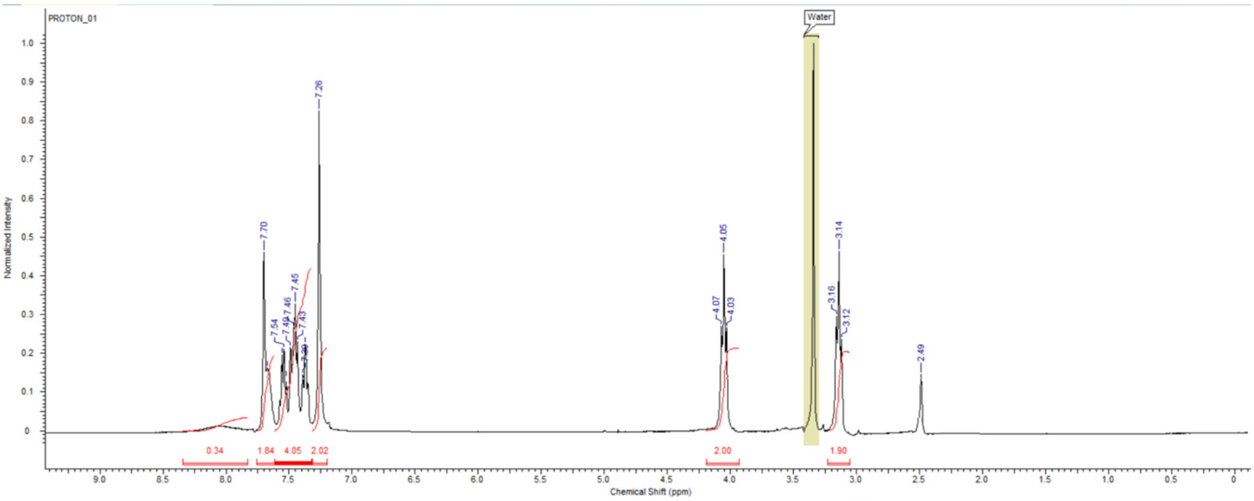

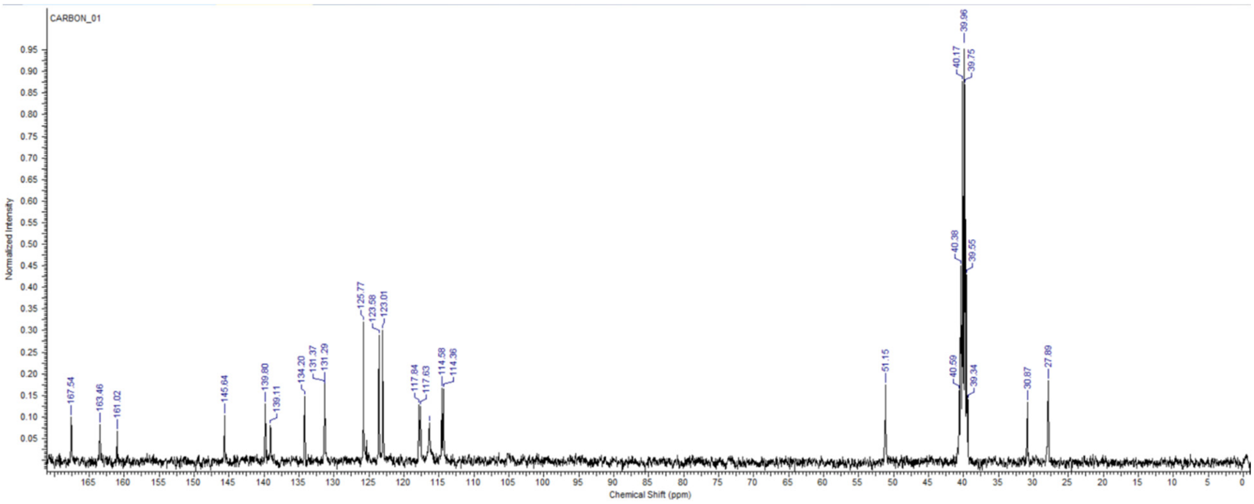

Chromatogram

ST-110 C:\LabSolutions\Data\CHEK\ST P1030-03.lcd

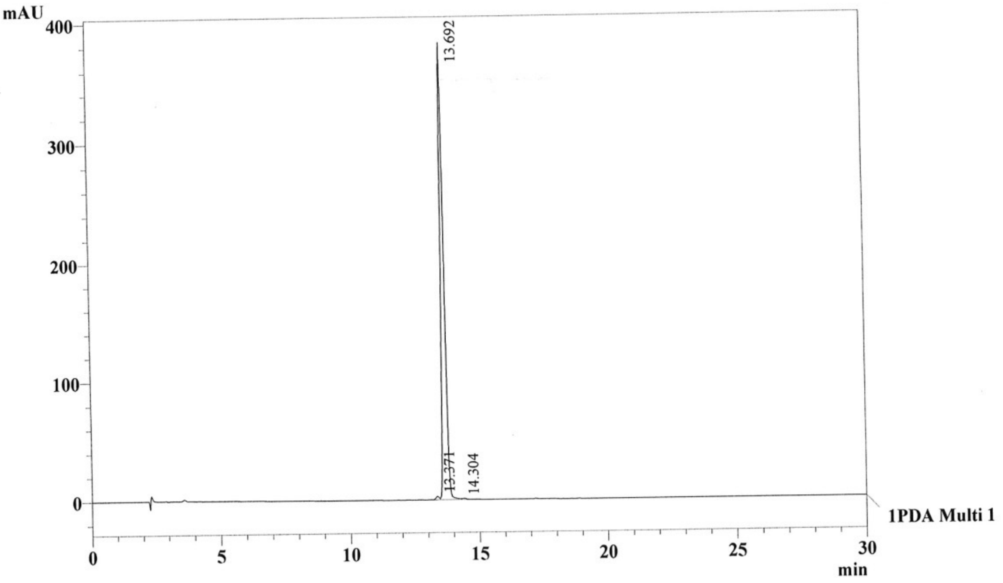

1 PDA Multi 1 / 300nm 4nm

PeakTable

PDA Ch1 300nm 4nm

Peak#	Ret. Time	Area	Height	Area %
1	13.371	23986	2782	0.683
2	13.692	3478688	382890	99.080
3	14.304	8303	590	0.236
Total		3510977	386261	100.000

Method

<<LC Program>>

Time	Unit	Command	Value
0.10	Pumps	B.Conc	30
30.00	Pumps	B.Conc	70
33.00	Pumps	B.Conc	30
45.00	Controller	Stop	

Method Filename : FOS B.lcm

Shimadzu LC-20AD; 2-System FOS, Colon Kromasil 100-C18,.size 5mkm, 4,6*250mm, N 86912
Elution: A - H3PO4 0.01M pH 2.6; B - MeCN, fl. 1,0 ml/min, loop 20mkl.

1-(4-fluorobenzoyl)indoline-5-sulfonamide **(4c)**

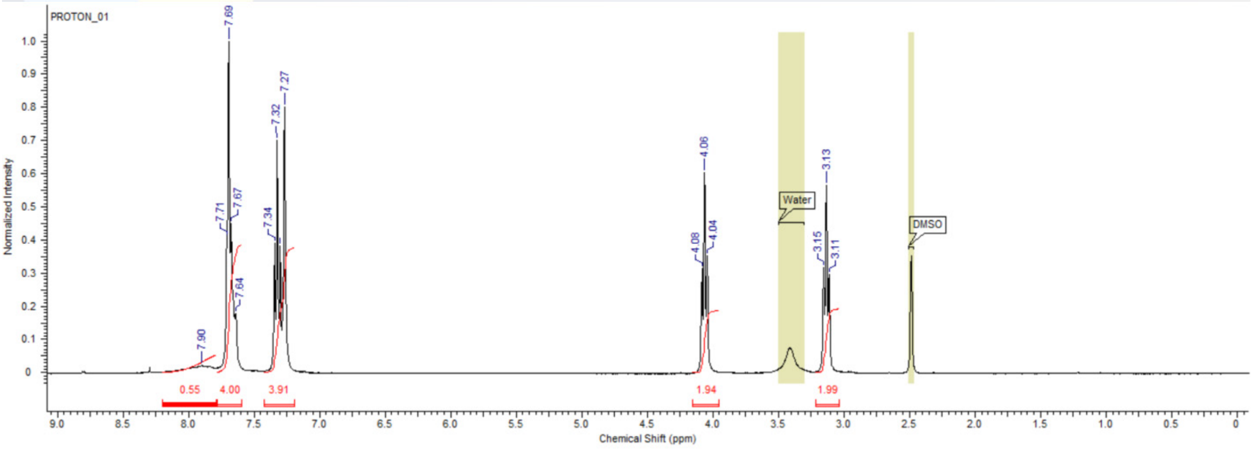

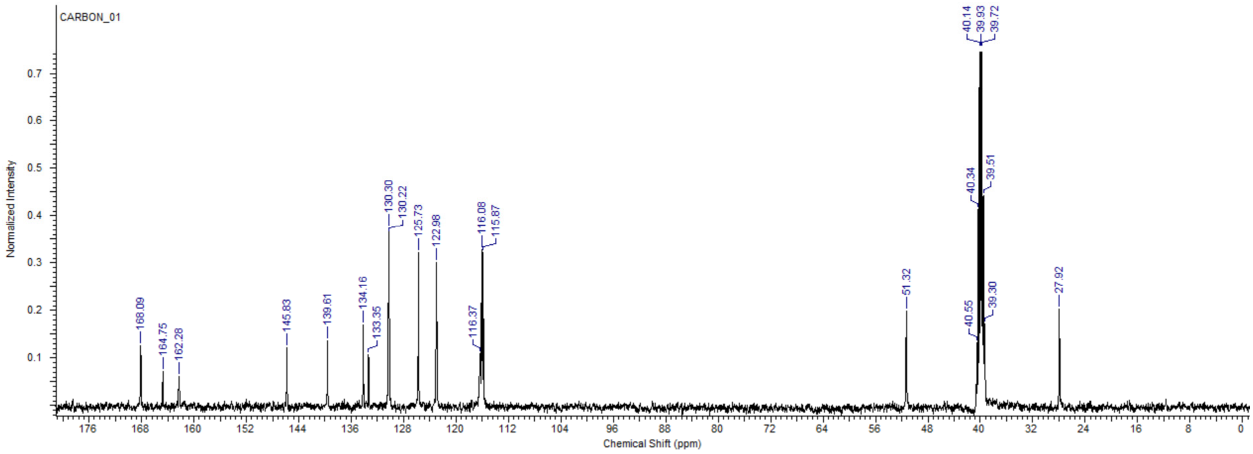

Chromatogram
ST-98 C:\LabSolutions\Data\CHEK\ST P1002-007.lcd

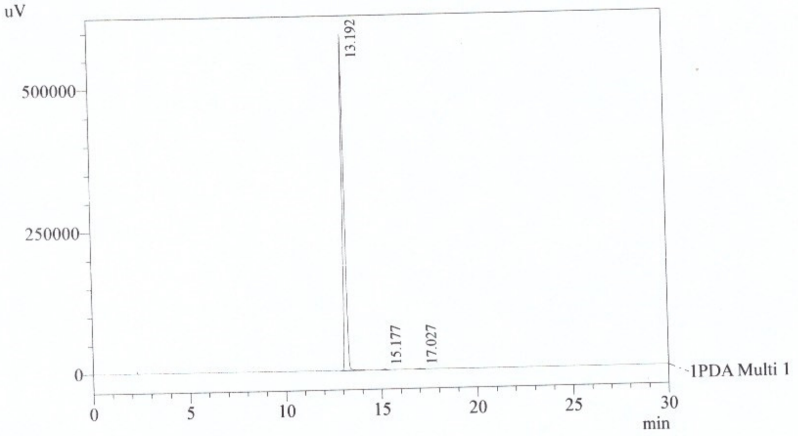

1 PDA Multi 1 / 300nm 4nm

PDA Ch1 300nm 4nm

Peak#	Ret. Time	Area	Height	Area %
1	13.192	4940418	594007	99.603
2	15.177	12191	1501	0.246
3	17.027	7507	946	0.151
Total		4960116	596454	100.000

Method Filename : FOS B.lcm 05.10.2020 14:44:25

Time	Unit	Command	Valu
0.01	Pumps	B.Conc	30
30.00	Pumps	B.Conc	70
33.00	Pumps	B.Conc	30
45.00	Controller	Stop	

Shimadzu LC-20 AD; System - FOS Colon- Kromasil-100-5mkm. C-18, 4,6x250 mm. N 62511
Elution: A - H3PO4 0,01M pH 2,6; B - MeCN, fl - 1.0 ml/min, loop 20 mkl

1-(2,6-difluorobenzoyl)indoline-5-sulfonamide **(4d)**

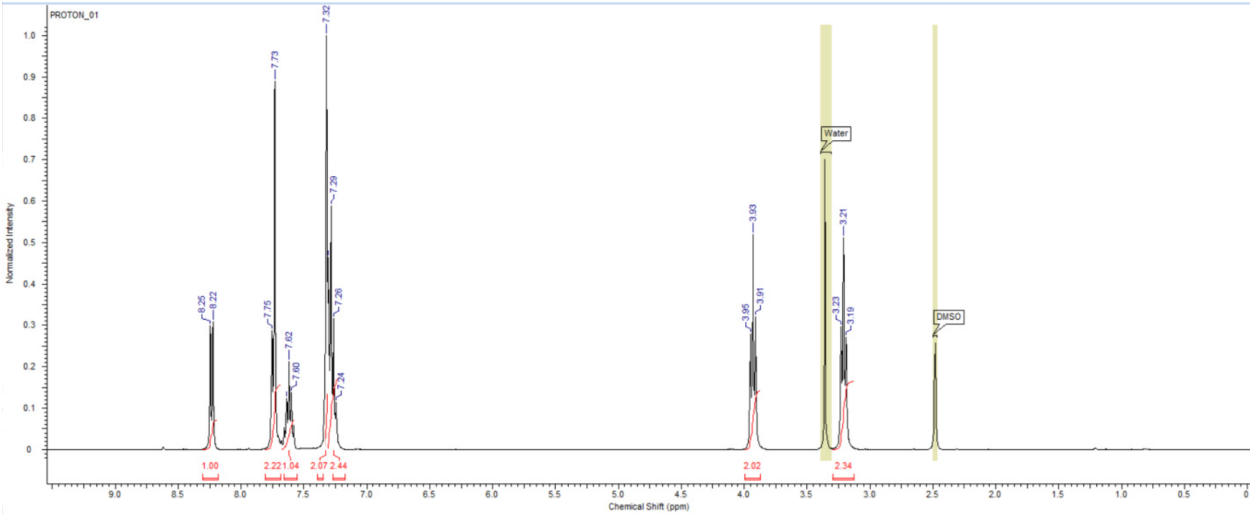

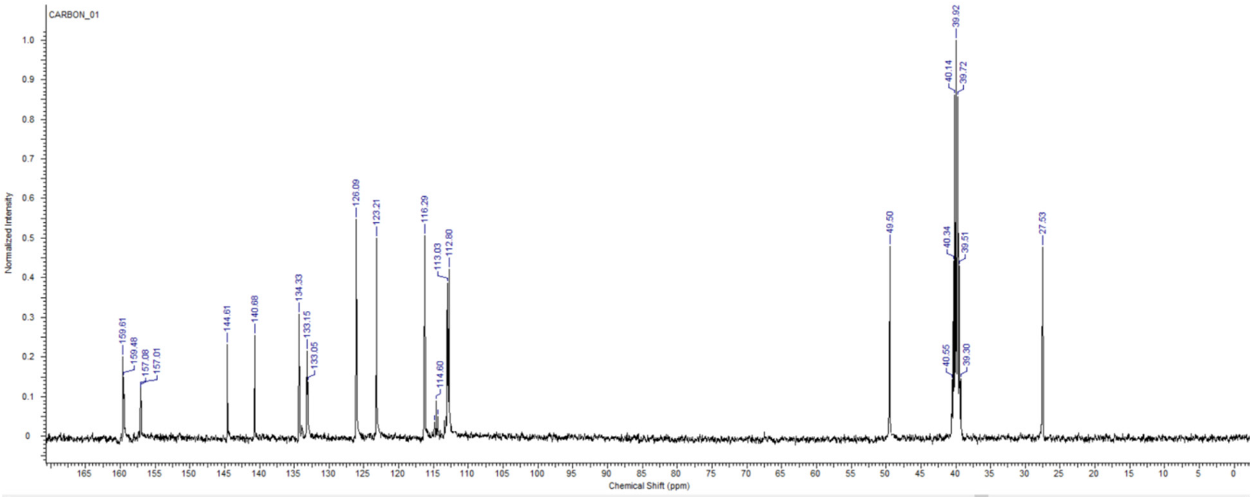

Chromatogram
ST-93 C:\LabSolutions\Data\CHEK\ST P1002-002.lcd

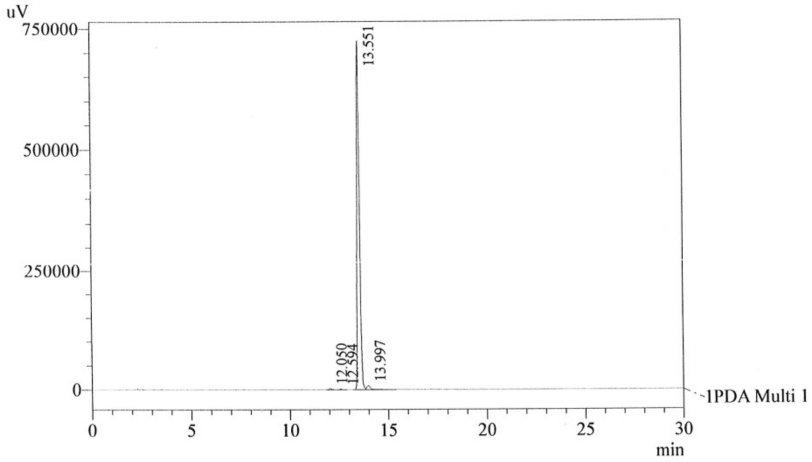

1 PDA Multi 1 / 270nm 4nm

PDA Ch1 270nm 4nm

Peak#	Ret. Time	Area	Height	Area %
1	12.050	26734	2569	0.382
2	12.594	13156	1519	0.188
3	13.551	6835590	725024	97.689
4	13.997	121848	8601	1.741
Total		6997327	737713	100.000

Method Filename : FOS B.lcm 02.10.2020 14:08:30

Time	Unit	Command	Valu
0.01	Pumps	B.Conc	30
30.00	Pumps	B.Conc	70
33.00	Pumps	B.Conc	30
45.00	Controller	Stop	

Shimadzu LC-20 AD; System - FOS Colon- Kromasil-100-5mkm. C-18, 4,6x250 mm. N 62511
Elution: A - H3PO4 0,01M pH 2,6; B - MeCN, fl - 1.0 ml/min, loop 20 mkl

1-(perfluorobenzoyl)indoline-5-sulfonamide **(4e)**

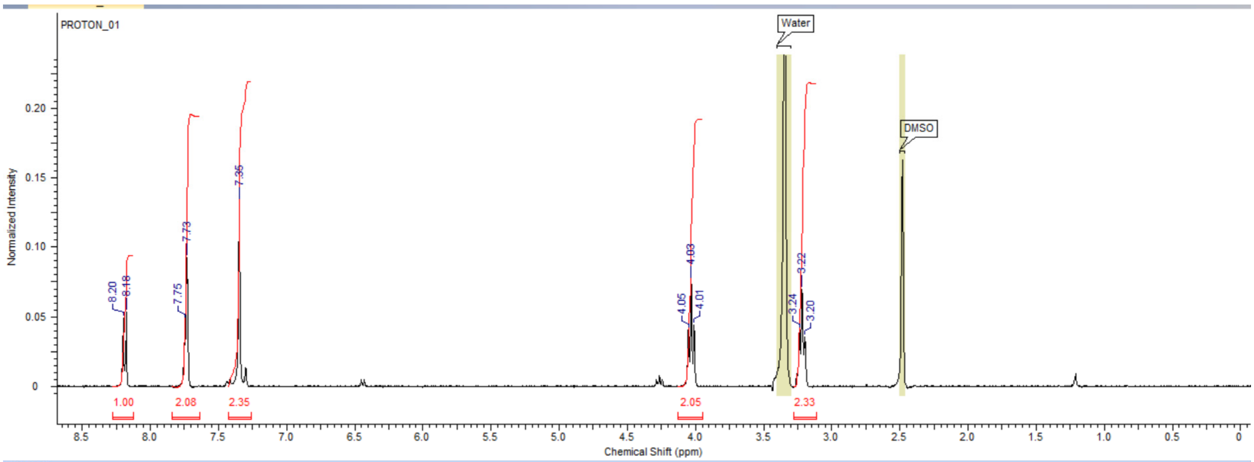

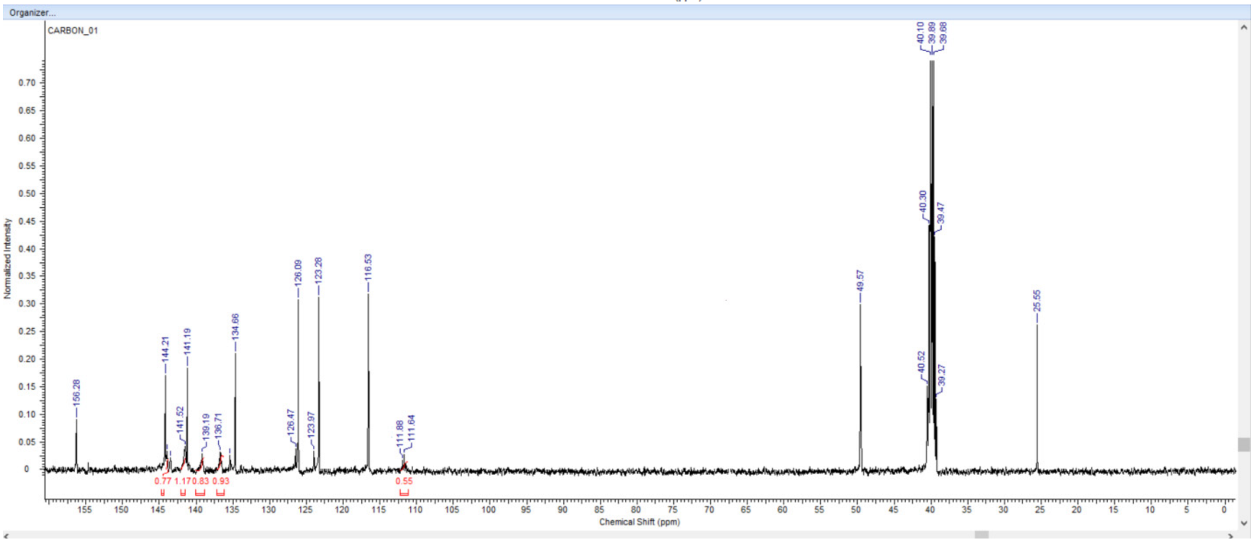

Title: **ST-12 3238

Пн Ноя 07 12:36:46 2022 (GM

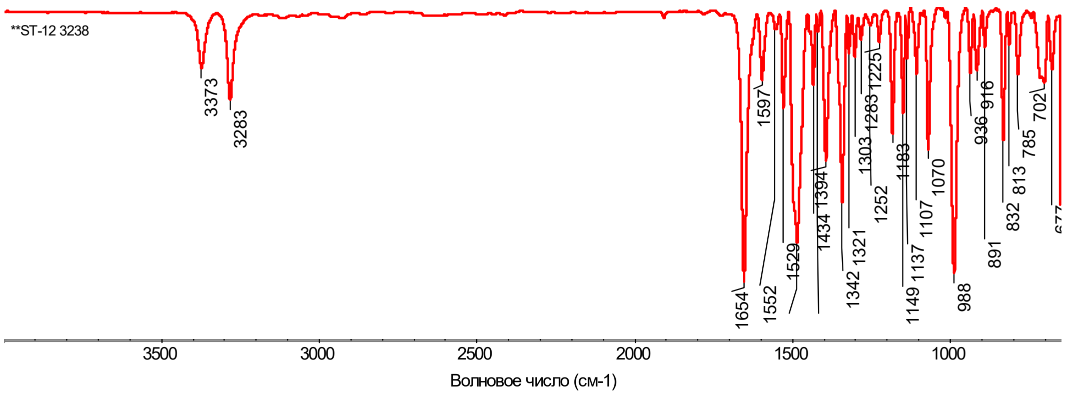

Expanded fingerprint region:

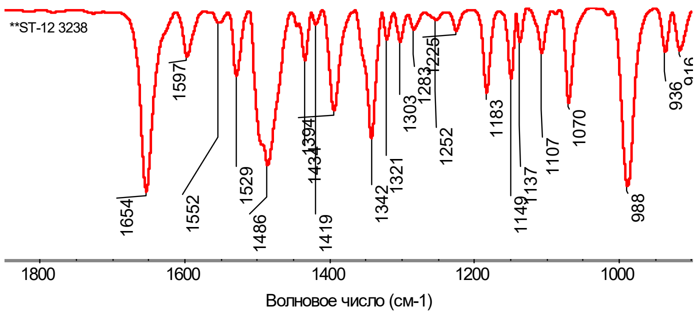

Время измерения: Пн Ноя 07 12:15:28 2022
Число сканов пробы: 64
Число сканов сравнения: 64
Разрешение: 4.000
Усиление: 8.0
Скорость зеркала: 0.4747
Диафрагма: 150.00

Chromatogram
ST-12 C:\LabSolutions\Data\CHEK\ST O1018-02.lcd

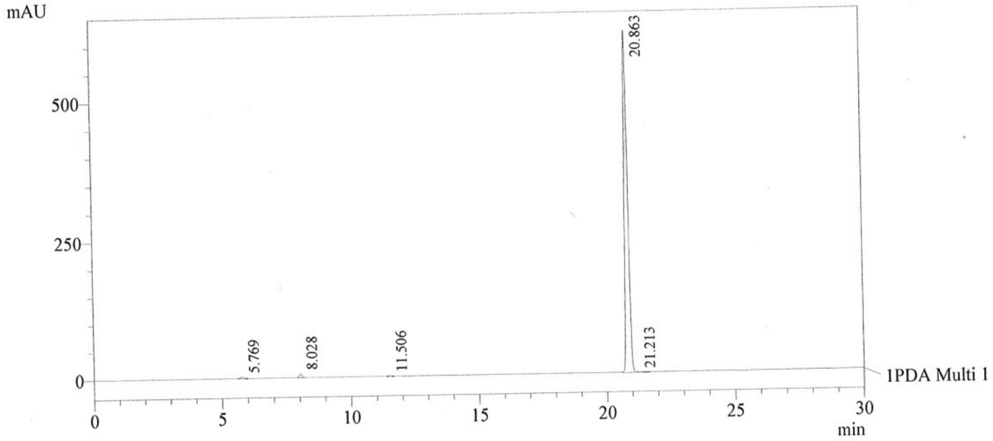

1 PDA Multi 1 / 300nm 4nm

PeakTable

PDA Ch1 300nm 4nm

Peak#	Ret. Time	Area	Height	Area %
1	5.769	22824	2434	0.399
2	8.028	45020	6624	0.787
3	11.506	7331	1074	0.128
4	20.863	5640910	615704	98.552
5	21.213	7713	662	0.135
Total		5723799	626499	100.000

Method

<<LC Program>>

Time	Unit	Command	Value
0.10	Pumps	B.Conc	30
30.00	Pumps	B.Conc	70
33.00	Pumps	B.Conc	30
45.00	Controller	Stop	

Sample Information

Method Filename : FRA02 Bv A.lcm
Date Acquired : 18.10.2019

Shimadzu LC-20AD; 2-System FRA02vA, Colon Kromasil 100-C18,.size 5mkm, 4,6*250mm, N 44650
Elution: A - COOHNH4 0,2% pH4.5 : B - MeCN, fl. 1,0 ml/min, loop 20mkl.

1-(3-chlorobenzoyl)indoline-5-sulfonamide **(4f)**

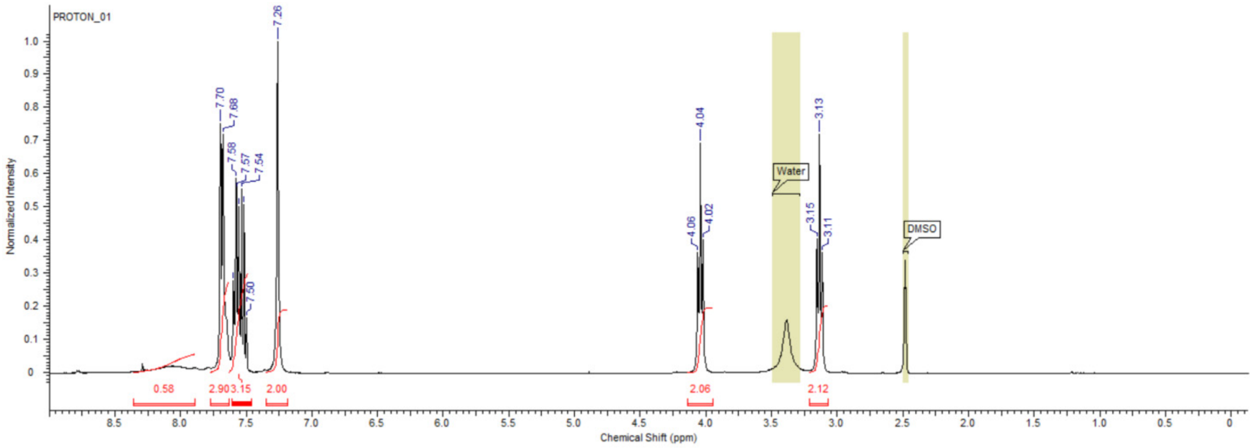

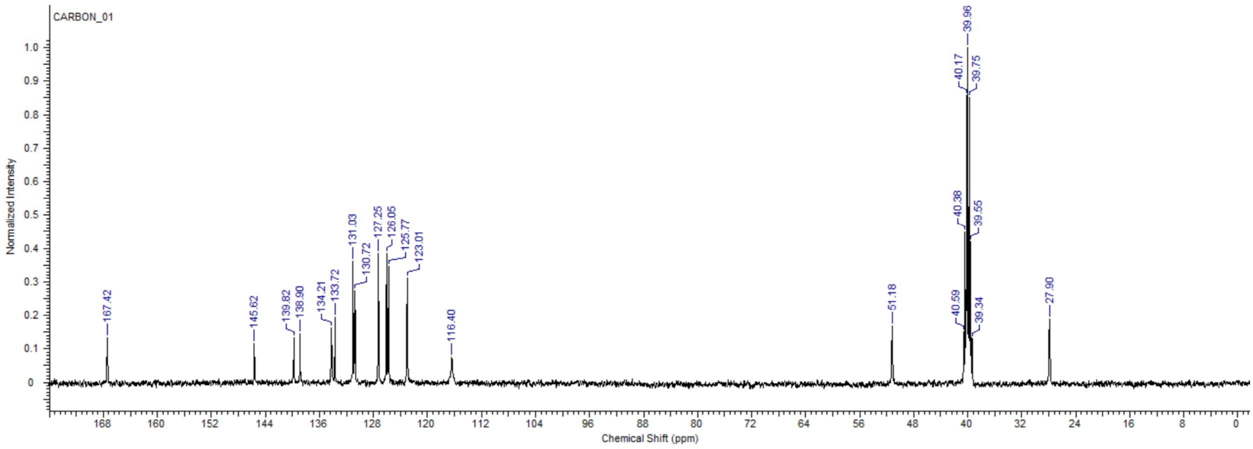

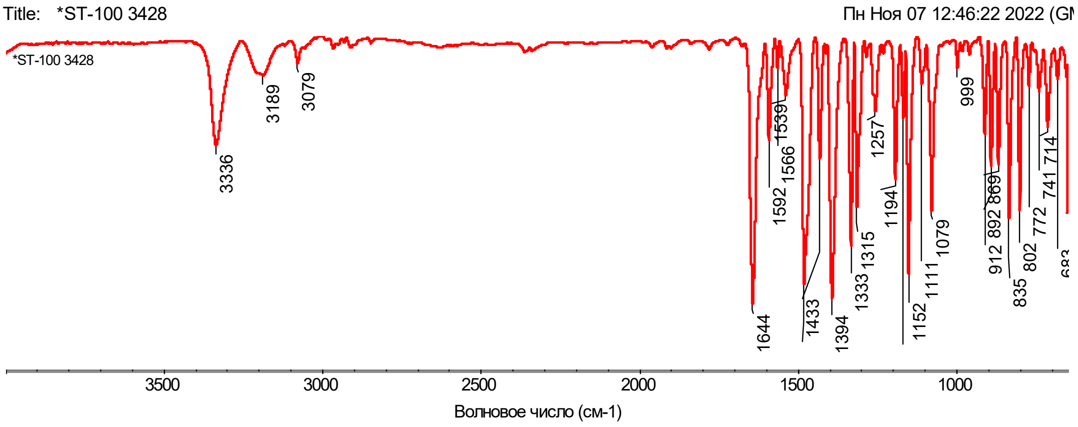

Expanded fingerprint region:

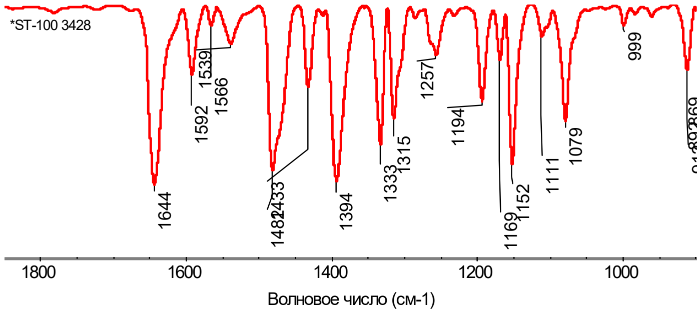

Время измерения: Пн Ноя 07 12:43:28 2022
Число сканов пробы: 64
Число сканов сравнения: 64
Разрешение: 4.000
Усиление: 8.0
Скорость зеркала: 0.4747
Диафрагма: 150.00

Chromatogram
ST-100 C:\LabSolutions\Data\CHEK\ST P0906-002.lcd

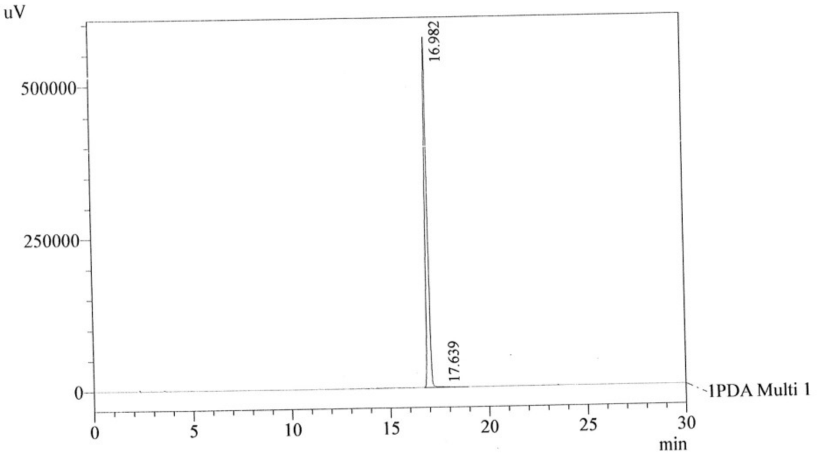

1 PDA Multi 1 / 300nm 4nm

PDA Ch1 300nm 4nm

Peak#	Ret. Time	Area	Height	Area %
1	16.982	5018288	574643	99.560
2	17.639	22195	1035	0.440
Total		5040483	575678	100.000

Method Filename : FOS B.lcm 06.10.2020 14:10:26

Time	Unit	Command	Valu
0.01	Pumps	B.Conc	30
30.00	Pumps	B.Conc	70
33.00	Pumps	B.Conc	30
45.00	Controller	Stop	

Shimadzu LC-20 AD; System - FOS Colon- Kromasil-100-5mkm. C-18, 4,6x250 mm. N 62511
Elution: A - H3PO4 0,01M pH 2,6; B - MeCN, fl - 1.0 ml/min, loop 20 mkl

1-(4-chlorobenzoyl)indoline-5-sulfonamide **(4g)**

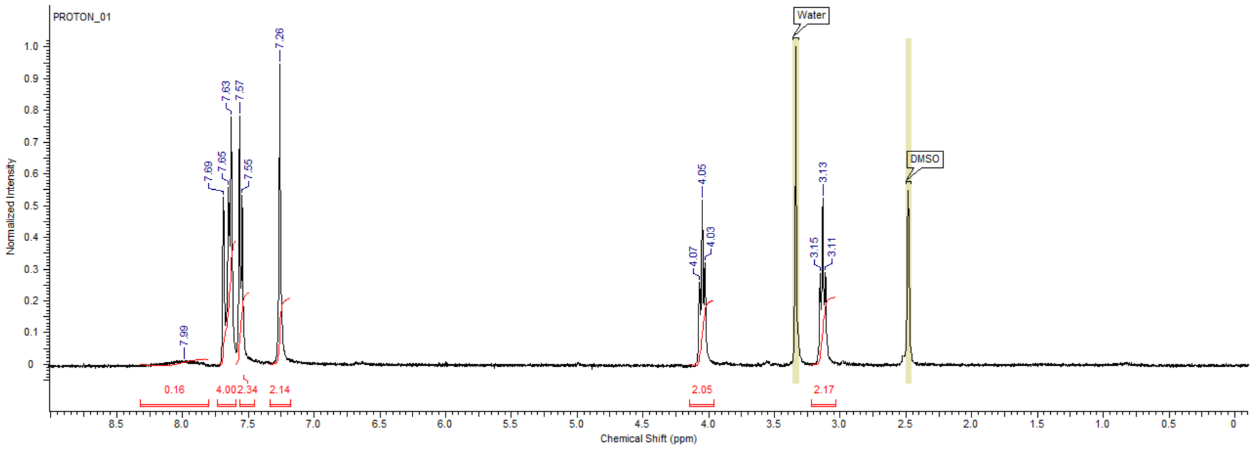

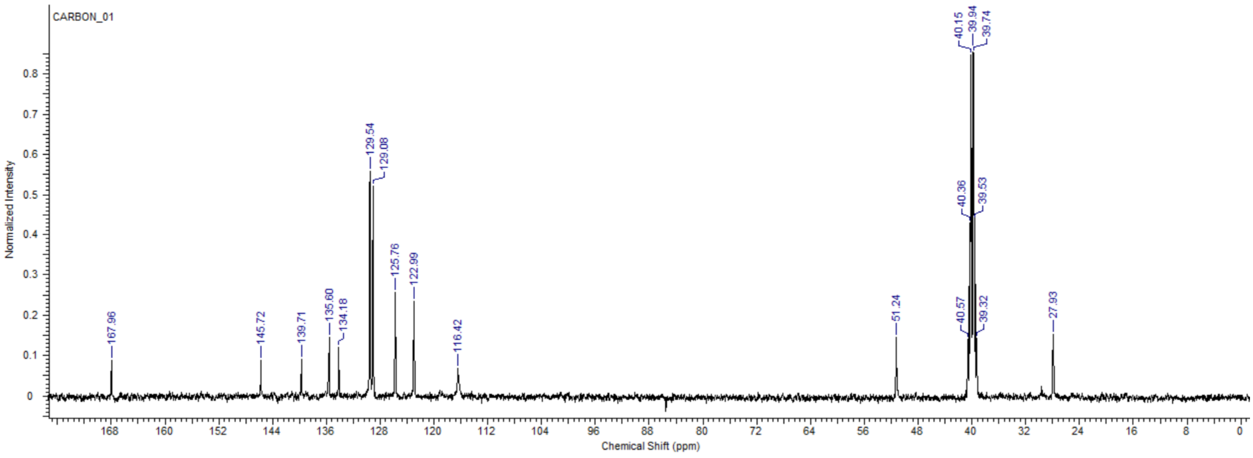

Chromatogram
ST-97 C:\LabSolutions\Data\CHEK\ST P1002-006.lcd

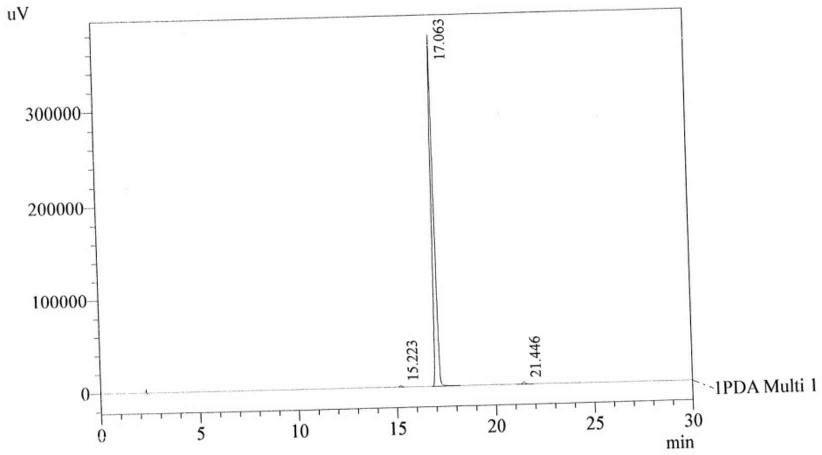

1 PDA Multi 1 / 300nm 4nm

PDA Ch1 300nm 4nm

Peak#	Ret. Time	Area	Height	Area %
1	15.223	13170	1606	0.405
2	17.063	3213095	374424	98.863
3	21.446	23791	2491	0.732
Total		3250056	378520	100.000

Method Filename : FOS B.lcm 05.10.2020 13:53:48

Time	Unit	Command	Valu
0.01	Pumps	B.Conc	30
30.00	Pumps	B.Conc	70
33.00	Pumps	B.Conc	30
45.00	Controller	Stop	

Shimadzu LC-20 AD; System - FOS Colon- Kromasil-100-5mkm. C-18, 4,6x250 mm. N 62511
Elution: A - H3PO4 0,01M pH 2,6; B - MeCN, fl - 1.0 ml/min, loop 20 mkl

1-(3,4-dichlorobenzoyl)indoline-5-sulfonamide **(4h)**

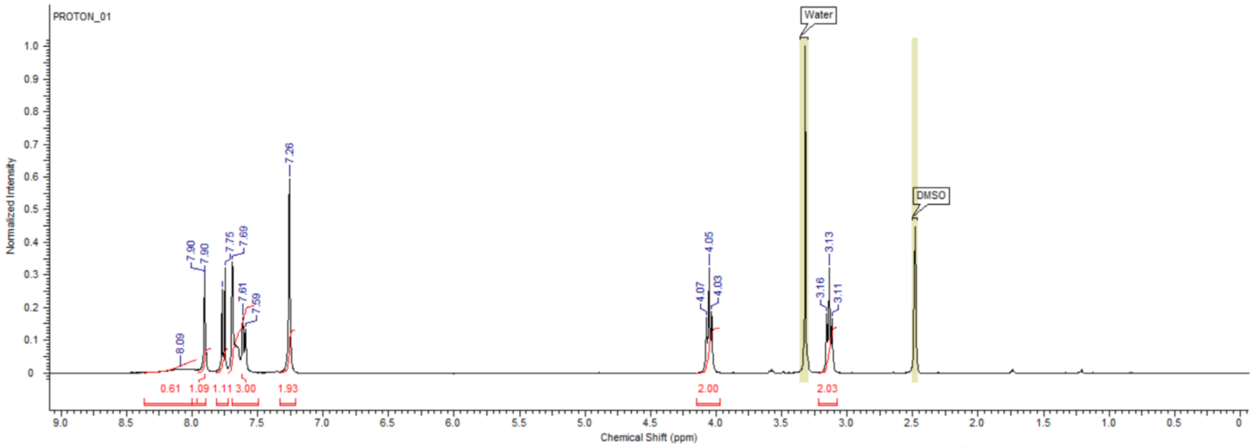

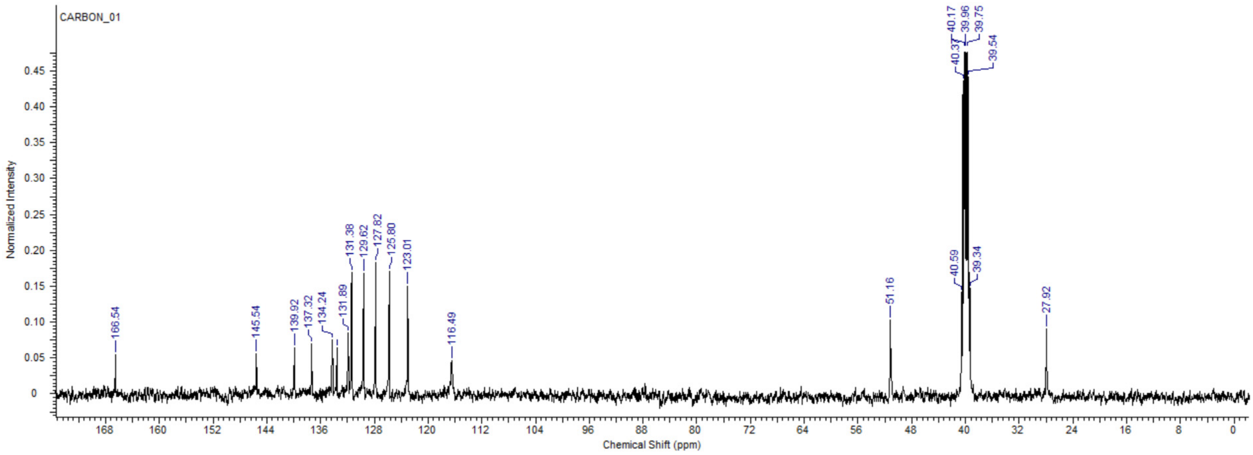

Chromatogram
ST-96 C:\LabSolutions\Data\CHEK\ST P1002-005.lcd

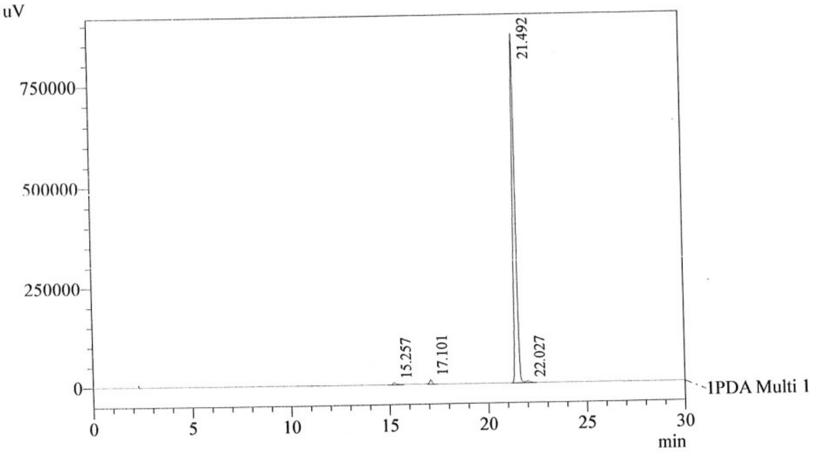

1 PDA Multi 1 / 300nm 4nm

PDA Ch1 300nm 4nm

Peak#	Ret. Time	Area	Height	Area %
1	15.257	66441	5083	0.778
2	17.101	96445	11095	1.129
3	21.492	8318492	869385	97.357
4	22.027	62979	4719	0.737
Total		8544356	890283	100.000

Method Filename : FOS B.lcm 05.10.2020 12:40:30

Time	Unit	Command	Valu
0.01	Pumps	B.Conc	30
30.00	Pumps	B.Conc	70
33.00	Pumps	B.Conc	30
45.00	Controller	Stop	

Shimadzu LC-20 AD; System - FOS Colon- Kromasil-100-5mkm. C-18, 4,6x250 mm. N 62511
Elution: A - H3PO4 0,01M pH 2,6; B - MeCN, fl - 1.0 ml/min, loop 20 mkl

1-(3-methylbenzoyl)indoline-5-sulfonamide **(4i)**

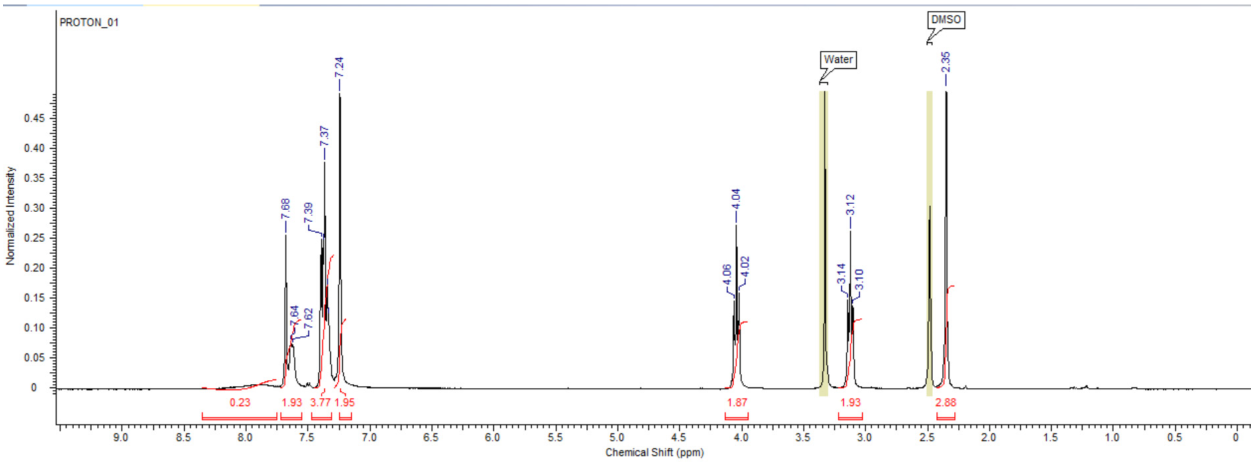

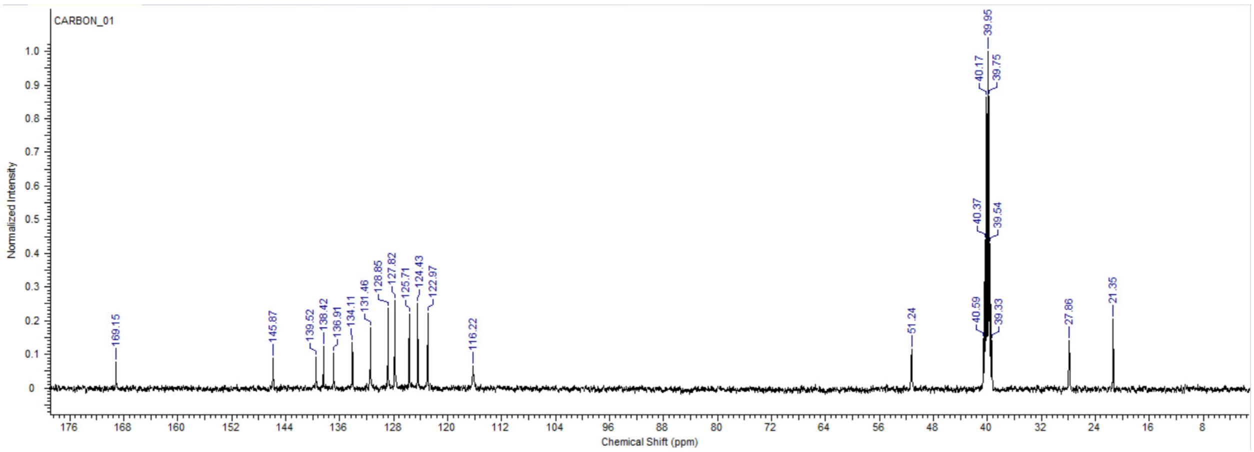

Chromatogram
ST-95 C:\LabSolutions\Data\CHEK\ST P1002-004.lcd

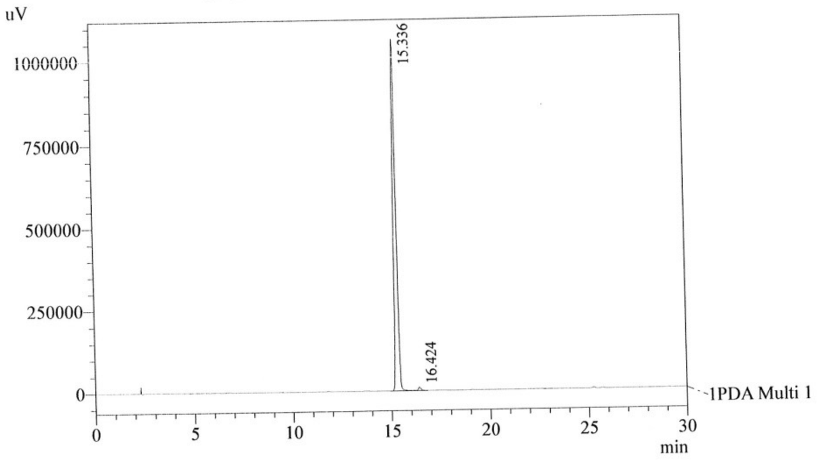

1 PDA Multi 1 / 300nm 4nm

PDA Ch1 300nm 4nm

Peak#	Ret. Time	Area	Height	Area %
1	15.336	10702805	1061615	99.000
2	16.424	108055	9919	1.000
Total		10810860	1071534	100.000

Method Filename : FOS B.lcm 05.10.2020 11:52:51

Time	Unit	Command	Valu
0.01	Pumps	B.Conc	30
30.00	Pumps	B.Conc	70
33.00	Pumps	B.Conc	30
45.00	Controller	Stop	

Shimadzu LC-20 AD; System - FOS Colon- Kromasil-100-5mkm. C-18, 4,6x250 mm. N 62511
Elution: A - H_3PO_4 0,01M pH 2,6; B - MeCN, fl - 1.0 ml/min, loop 20 mkl

1-(3-methoxybenzoyl)indoline-5-sulfonamide **(4j)**

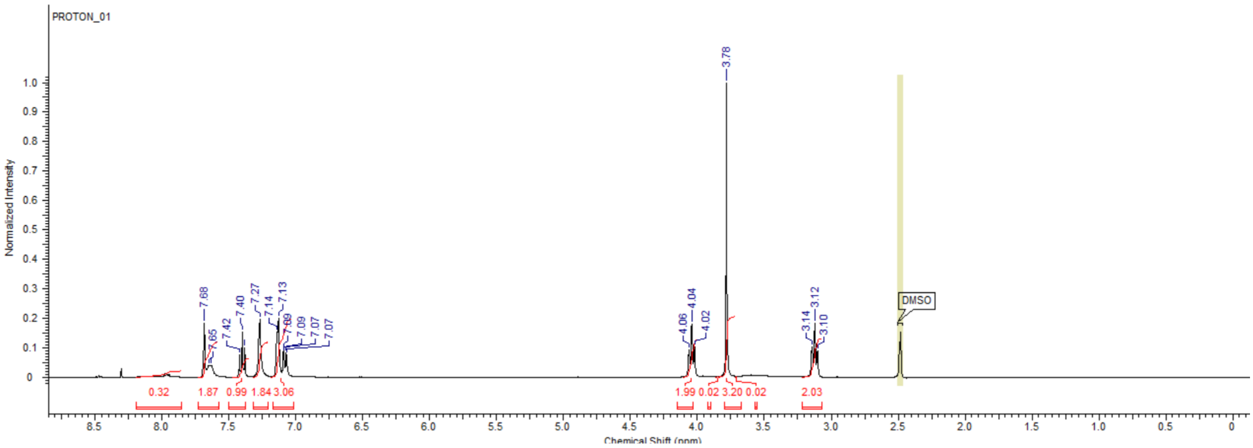

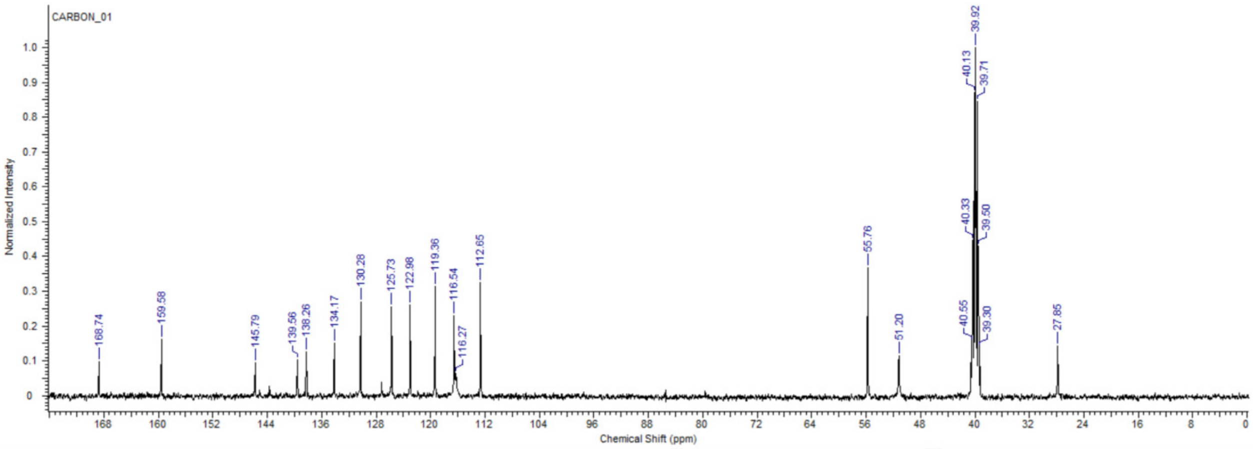

Chromatogram
ST-103 C:\LabSolutions\Data\CHEK\ST P1030-02.lcd

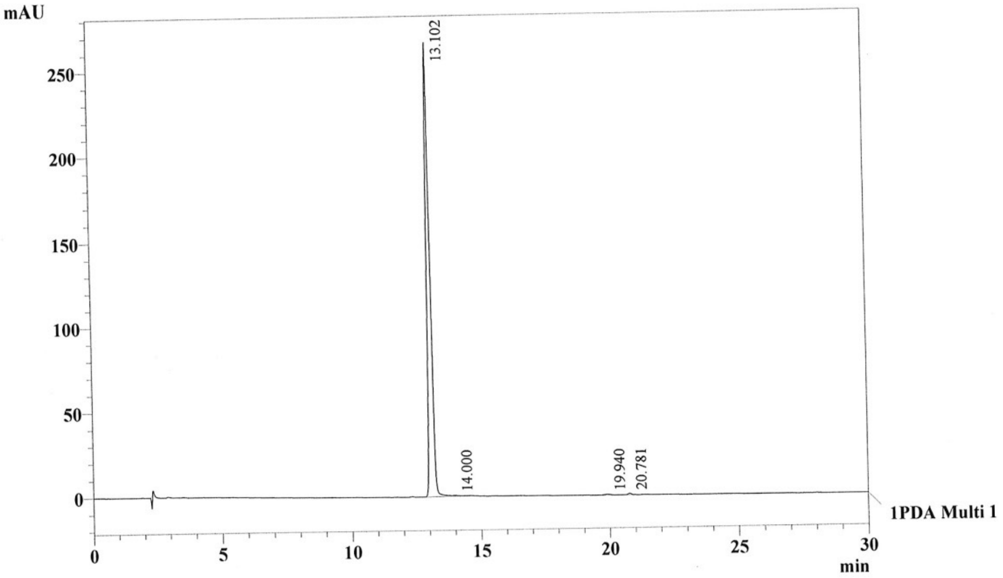

1 PDA Multi 1 / 300nm 4nm

PeakTable

PDA Ch1 300nm 4nm

Peak#	Ret. Time	Area	Height	Area %
1	13.102	2411337	267365	99.038
2	14.000	9469	434	0.389
3	19.940	6510	417	0.267
4	20.781	7454	834	0.306
Total		2434770	269050	100.000

Method

<<LC Program>>

Time	Unit	Command	Value
0.10	Pumps	B.Conc	30
30.00	Pumps	B.Conc	70
33.00	Pumps	B.Conc	30
45.00	Controller	Stop	

Method Filename : FOS B.lcm

Shimadzu LC-20AD; 2-System FOS, Colon Kromasil 100-C18,.size 5mkm, 4,6*250mm, N 86912
Elution: A - H3PO4 0.01M pH 2.6; B - MeCN, fl. 1,0 ml/min, loop 20mkl.

1-(4-methoxybenzoyl)indoline-5-sulfonamide **(4k)**

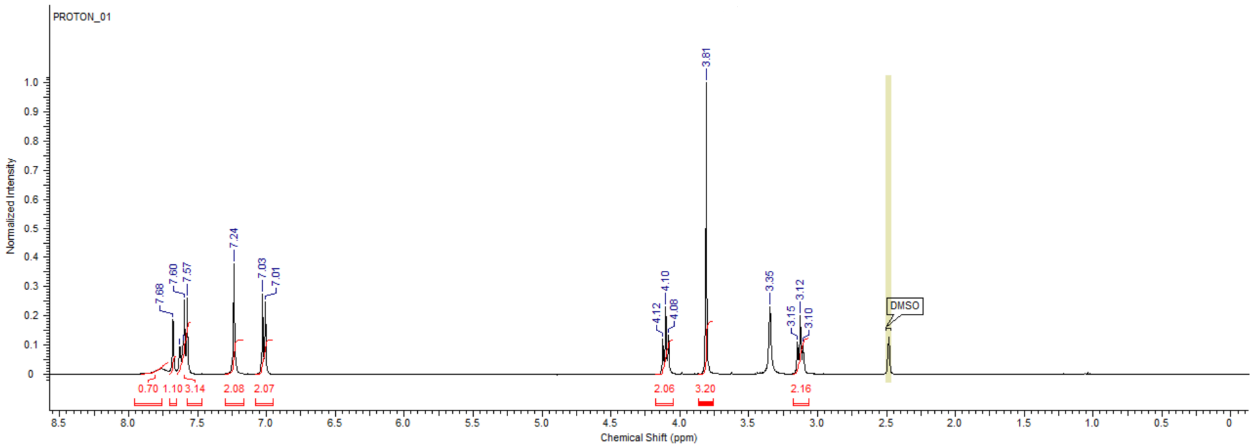

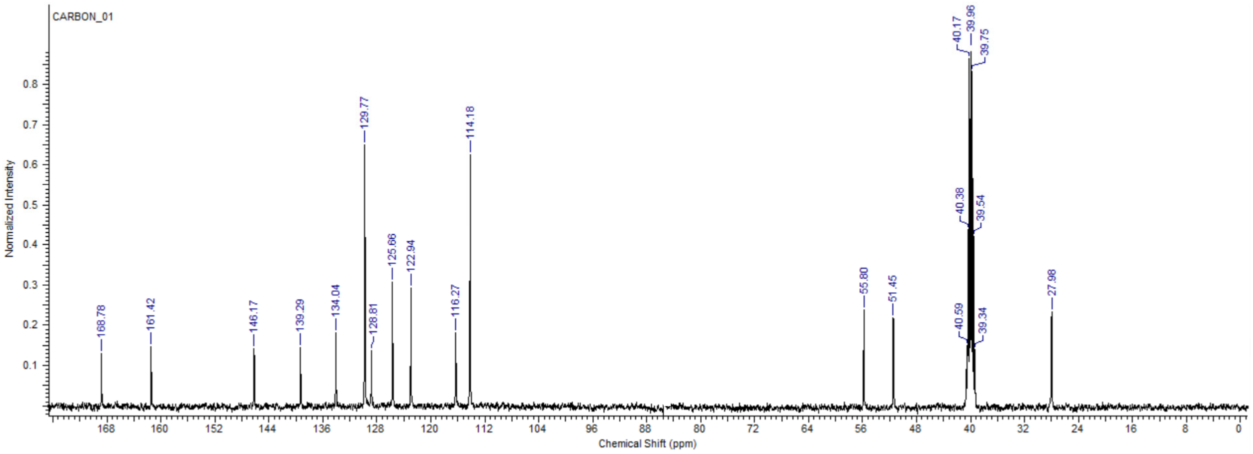

Chromatogram
ST-101 C:\LabSolutions\Data\CHEK\ST P0906-003.lcd

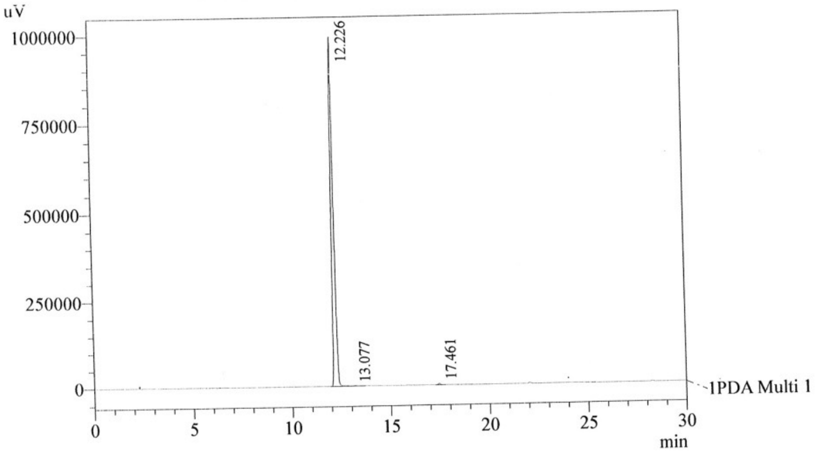

1 PDA Multi 1 / 300nm 4nm

PDA Ch1 300nm 4nm

Peak#	Ret. Time	Area	Height	Area %
1	12.226	8740989	992449	99.528
2	13.077	6703	418	0.076
3	17.461	34730	4455	0.395
Total		8782421	997321	100.000

Method Filename : FOS B.lcm 06.10.2020 14:50:25

Time	Unit	Command	Valu
0.01	Pumps	B.Conc	30
30.00	Pumps	B.Conc	70
33.00	Pumps	B.Conc	30
45.00	Controller	Stop	

Shimadzu LC-20 AD; System - FOS Colon- Kromasil-100-5mkm. C-18, 4,6x250 mm. N 62511
Elution: A - H3PO4 0,01M pH 2,6; B - MeCN, fl - 1.0 ml/min, loop 20 mkl

1-(2,4-dimethoxybenzoyl)indoline-5-sulfonamide **(4l)**

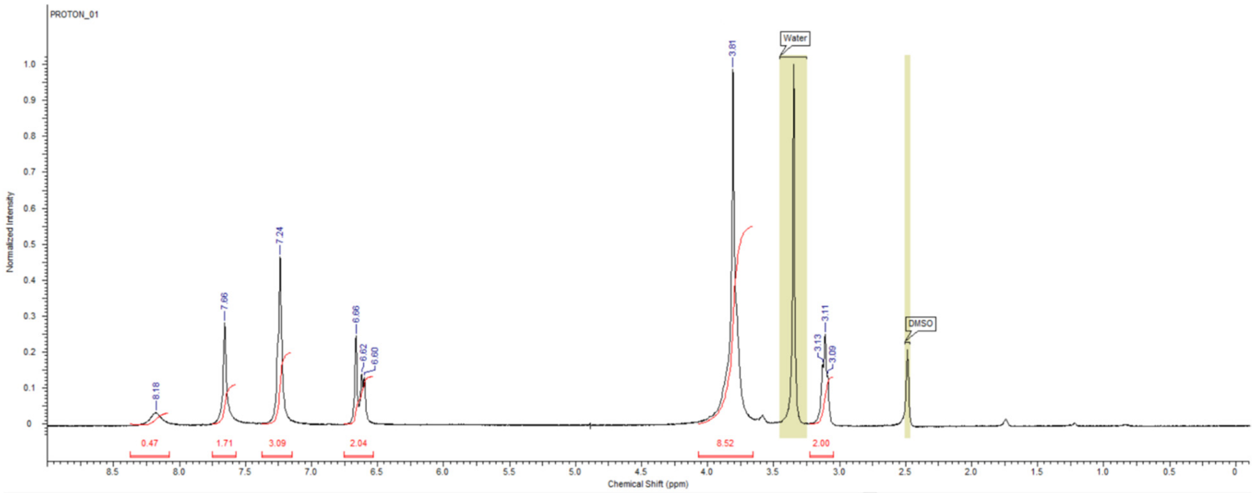

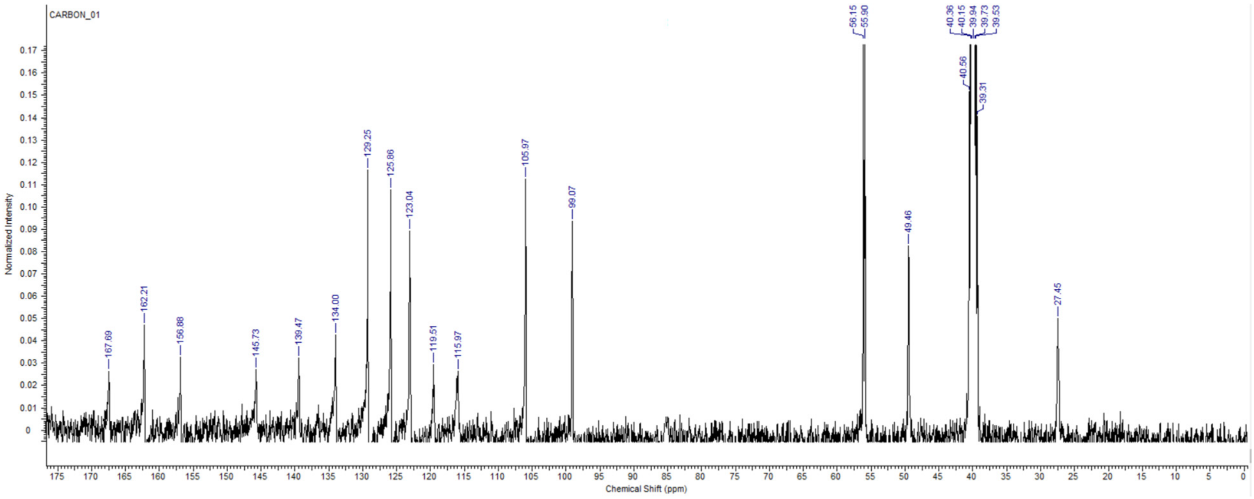

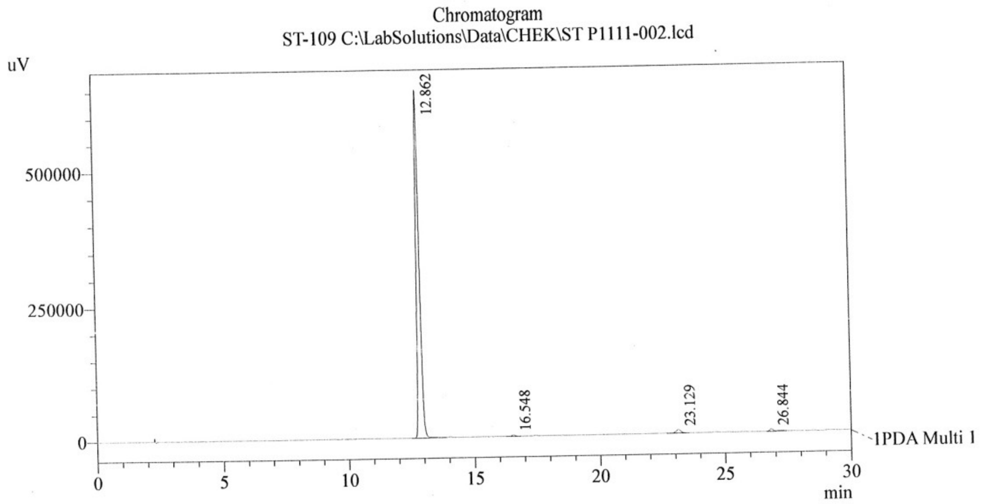

1 PDA Multi 1 / 300nm 4nm

PDA Ch1 300nm 4nm

Peak#	Ret. Time	Area	Height	Area %
1	12.862	5746904	647802	97.111
2	16.548	23329	2575	0.394
3	23.129	94185	6712	1.592
4	26.844	53446	4094	0.903
Total		5917864	661183	100.000

Method Filename : FOS B.lcm 11.11.2020 13:56:50

Time	Unit	Command	Valu
0.01	Pumps	B.Conc	30
30.00	Pumps	B.Conc	70
33.00	Pumps	B.Conc	30
45.00	Controller	Stop	

Shimadzu LC-20 AD; System - FOS Colon- Kromasil-100-5mkm. C-18, 4,6x250 mm. N 62511
Elution: A - H3PO4 0,01M pH 2,6; B - MeCN, fl - 1.0 ml/min, loop 20 mkl

1-(3,4,5-trimethoxybenzoyl)indoline-5-sulfonamide **(4m)**

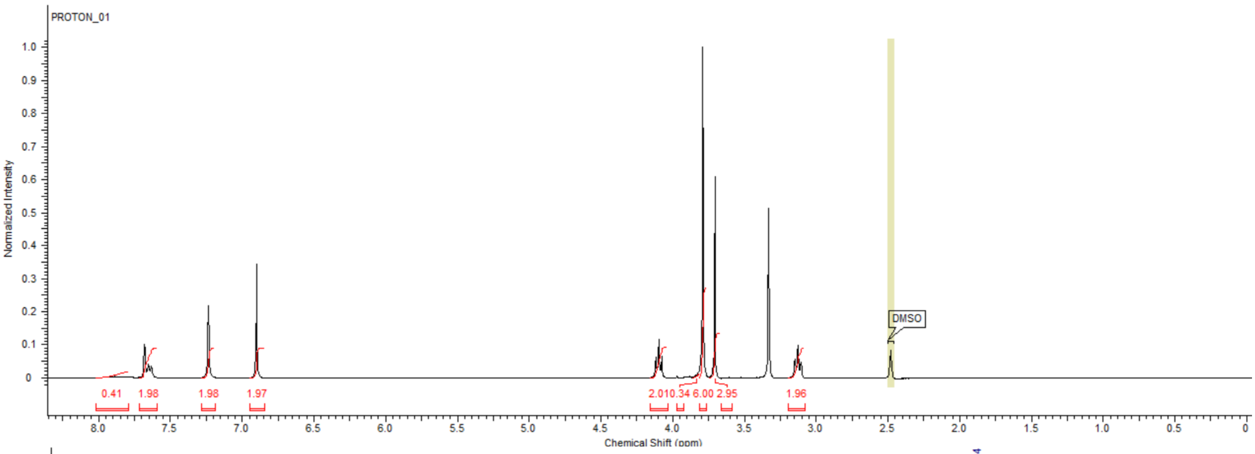

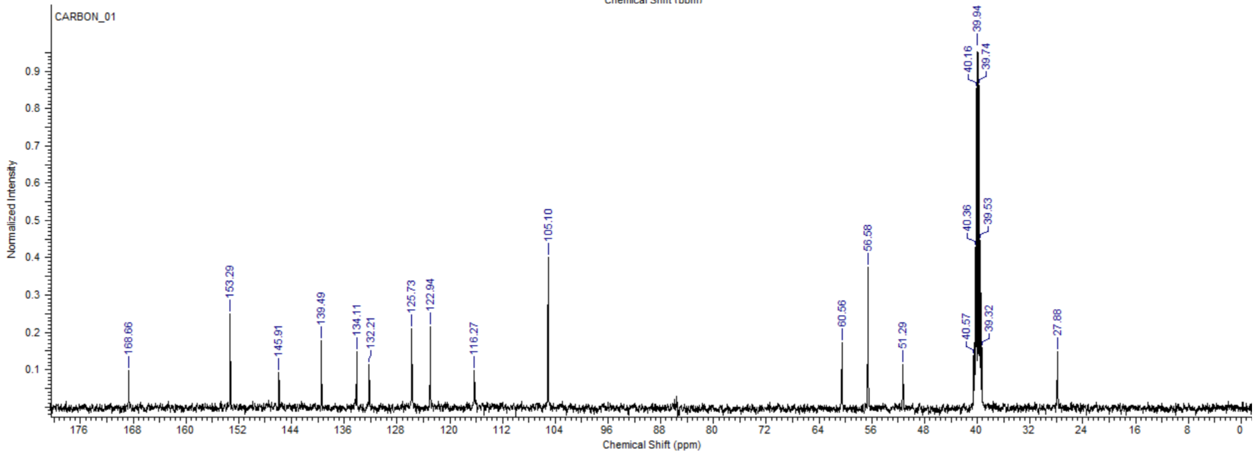

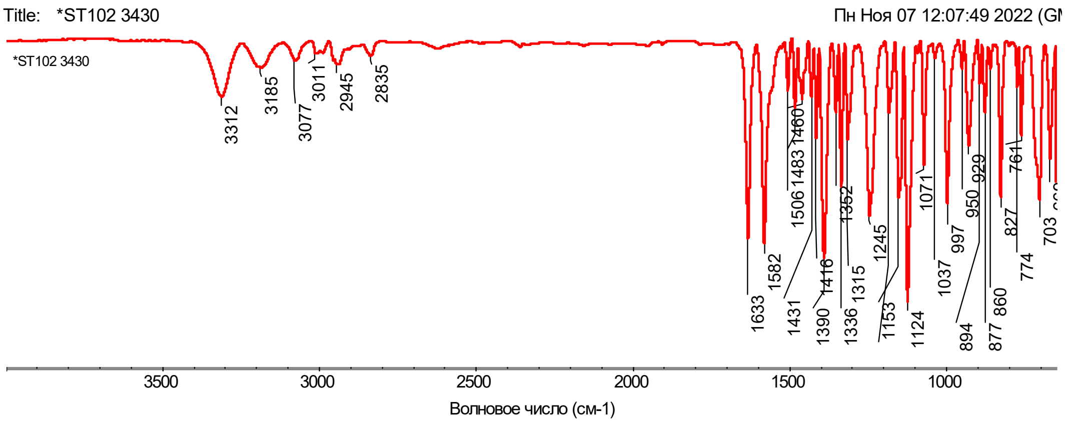

Expanded fingerprint region:

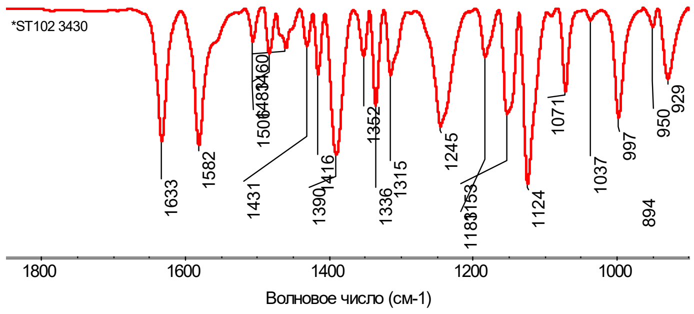

Время измерения: Пн Ноя 07 12:02:47 2022
Число сканов пробы: 64
Число сканов сравнения: 64
Разрешение: 4.000
Усиление: 8.0
Скорость зеркала: 0.4747
Диафрагма: 150.00

Chromatogram
ST-102 C:\LabSolutions\Data\CHEK\ST P0906-004.lcd

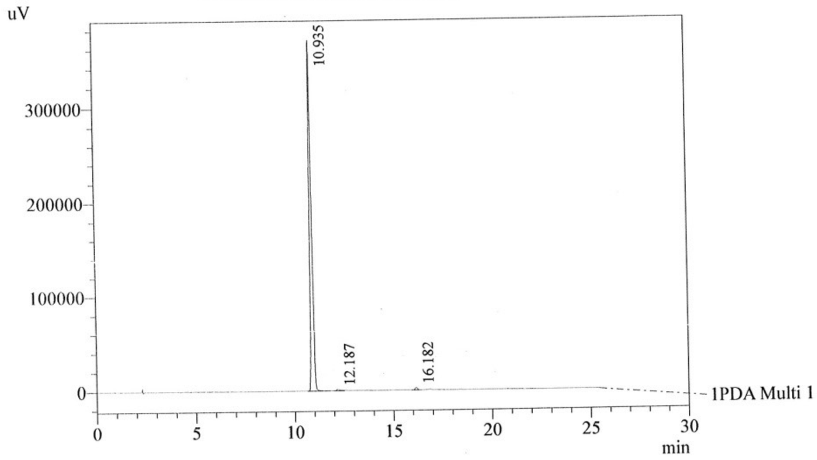

1 PDA Multi 1 / 300nm 4nm

PDA Ch1 300nm 4nm

Peak#	Ret. Time	Area	Height	Area %
1	10.935	2964625	369482	98.569
2	12.187	20123	1367	0.669
3	16.182	22931	2533	0.762
Total		3007679	373382	100.000

Method Filename : FOS B.lcm 06.10.2020 15:38:53

Time	Unit	Command	Valu
0.01	Pumps	B.Conc	30
30.00	Pumps	B.Conc	70
33.00	Pumps	B.Conc	30
45.00	Controller	Stop	

Shimadzu LC-20 AD; System - FOS Colon- Kromasil-100-5mkm. C-18, 4,6x250 mm. N 62511
Elution: A - H3PO4 0,01M pH 2,6; B - MeCN, fl - 1.0 ml/min, loop 20 mkl

1-(4-(methylthio)benzoyl)indoline-5-sulfonamide **(4n)**

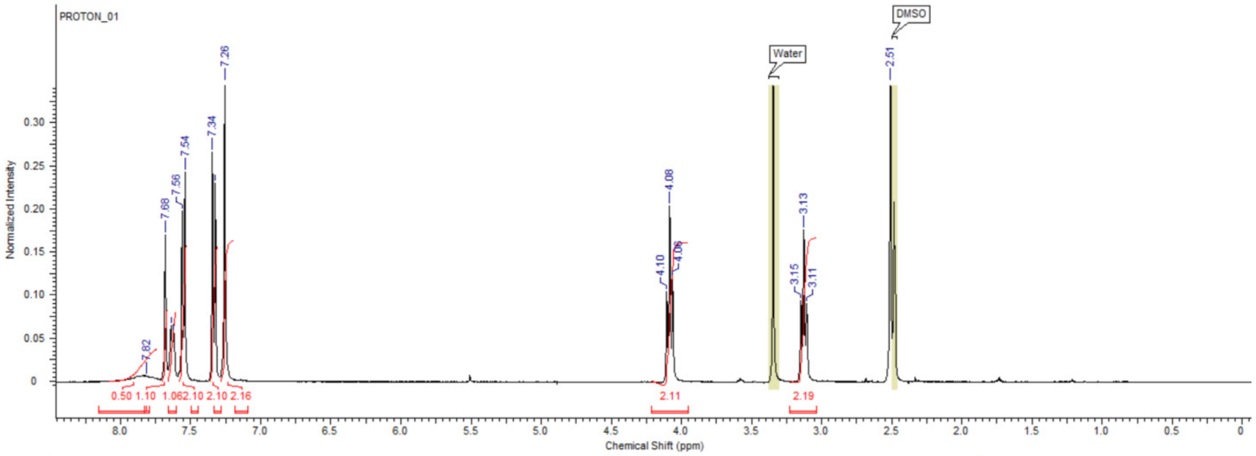

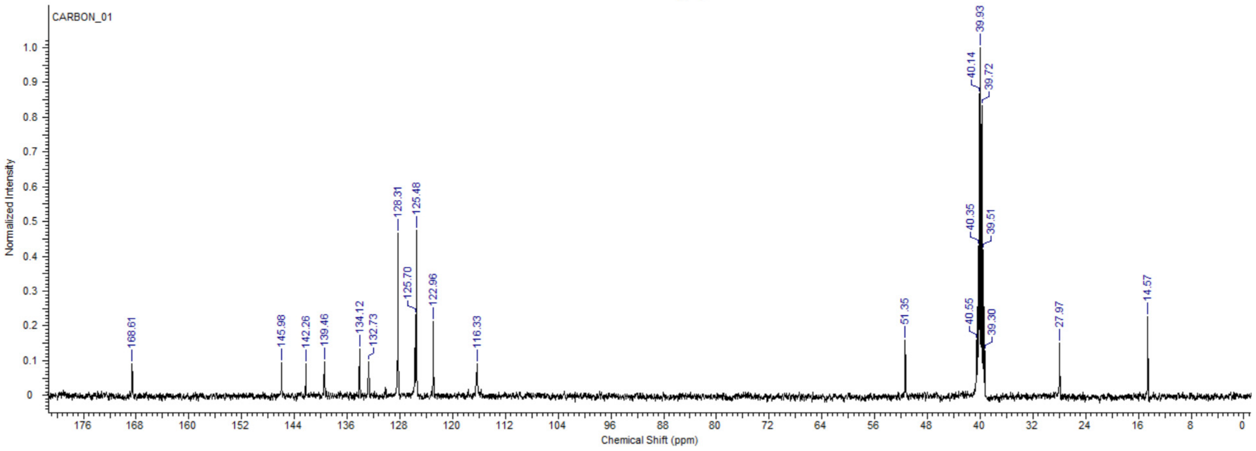

Chromatogram
ST-94 C:\LabSolutions\Data\CHEK\ST P1002-003.lcd

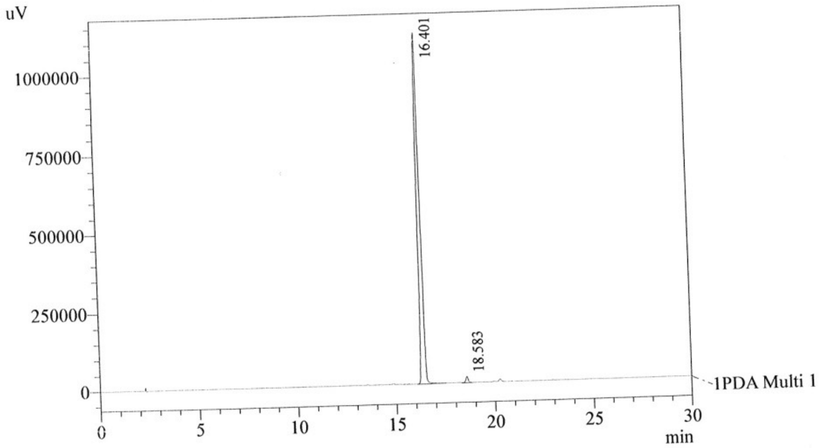

1 PDA Multi 1 / 300nm 4nm

PDA Ch1 300nm 4nm

Peak#	Ret. Time	Area	Height	Area %
1	16.401	11220008	1115012	98.577
2	18.583	161929	19590	1.423
Total		11381937	1134602	100.000

Method Filename : FOS B.lcm 02.10.2020 14:57:52

Time	Unit	Command	Valu
0.01	Pumps	B.Conc	30
30.00	Pumps	B.Conc	70
33.00	Pumps	B.Conc	30
45.00	Controller	Stop	

Shimadzu LC-20 AD; System - FOS Colon- Kromasil-100-5mkm. C-18, 4,6x250 mm. N 62511
Elution: A - H_3PO_4 0,01M pH 2,6; B - MeCN, fl - 1.0 ml/min, loop 20 mkl

1-(2-nitrobenzoyl)indoline-5-sulfonamide **(4o)**

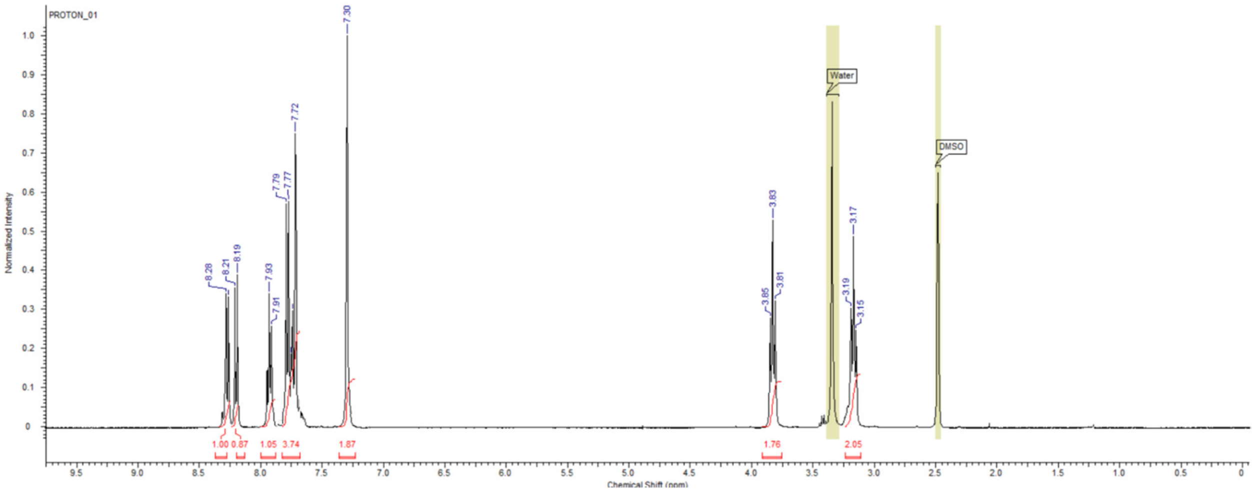

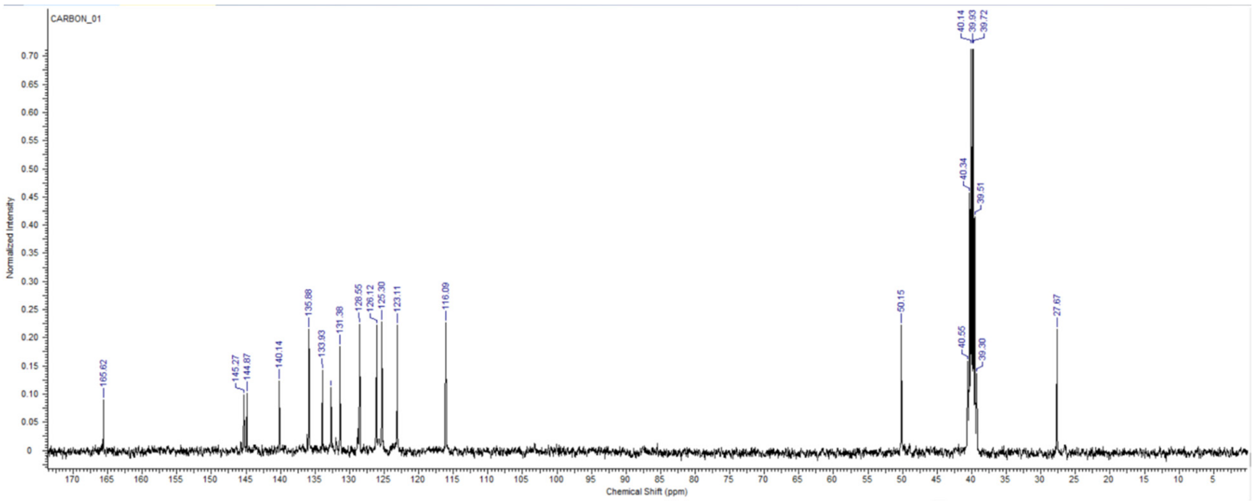

Chromatogram
ST-104 C:\LabSolutions\Data\CHEK\ST P1125-05.lcd

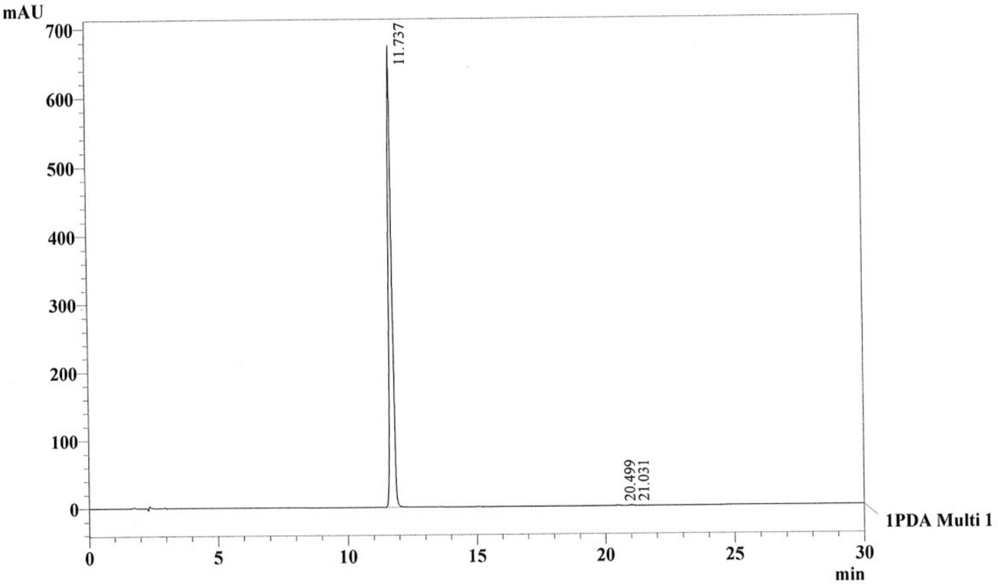

1 PDA Multi 1 / 270nm 4nm

PeakTable

PDA Ch1 270nm 4nm

Peak#	Ret. Time	Area	Height	Area %
1	11.737	5937221	674277	99.774
2	20.499	5545	637	0.093
3	21.031	7886	912	0.133
Total		5950653	675825	100.000

Method

<<LC Program>>

Time	Unit	Command	Value
0.10	Pumps	B.Conc	30
30.00	Pumps	B.Conc	70
33.00	Pumps	B.Conc	30
45.00	Controller	Stop	

Method Filename : FOS B.lcm

Shimadzu LC-20AD; 2-System FOS, Colon Kromasil 100-C18,.size 5mkm, 4,6*250mm, N 86912
Elution: A - H3PO4 0.01M pH 2.6; B - MeCN, fl. 1,0 ml/min, loop 20mkl.

1-(4-nitrobenzoyl)indoline-5-sulfonamide **(4p)**

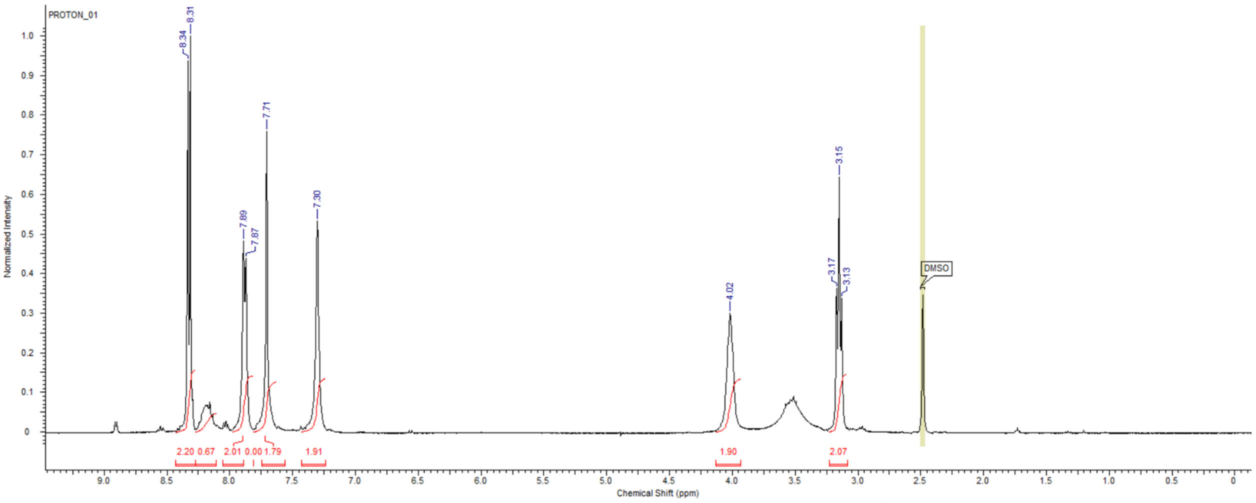

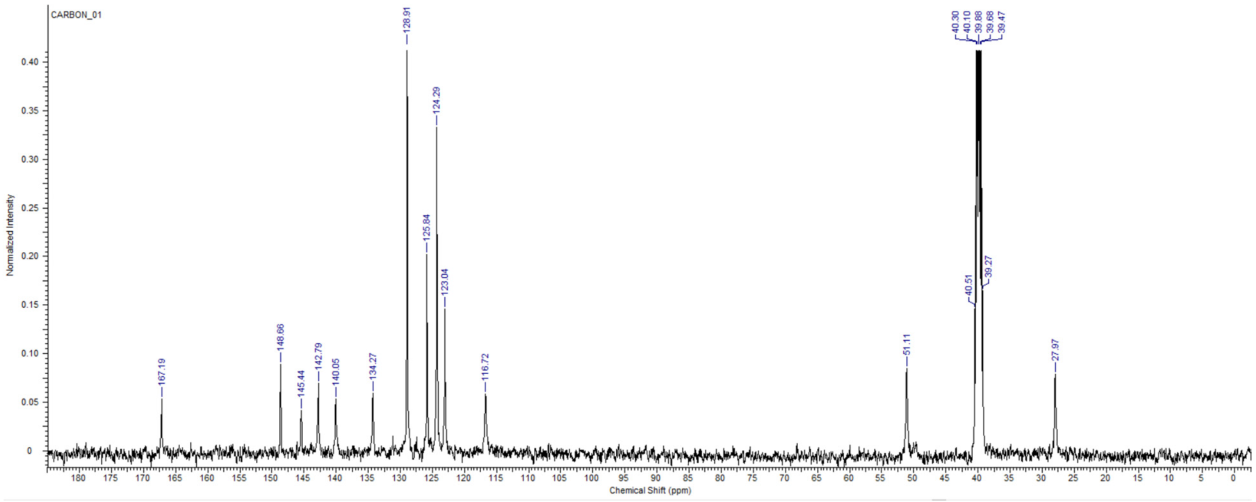

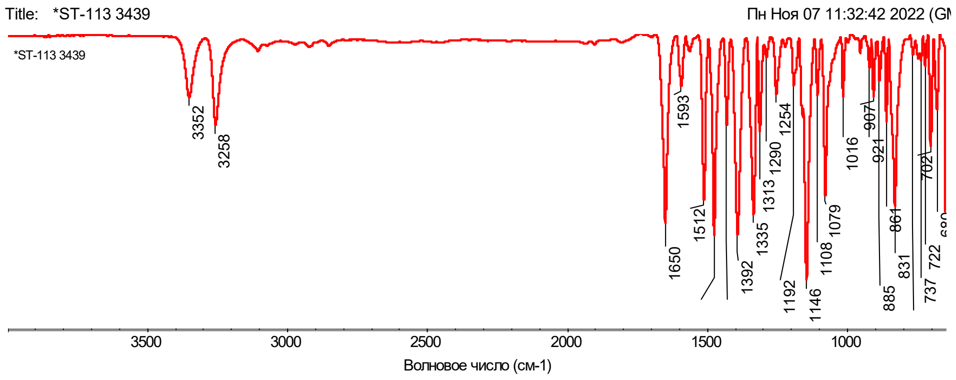

Expanded fingerprint region:

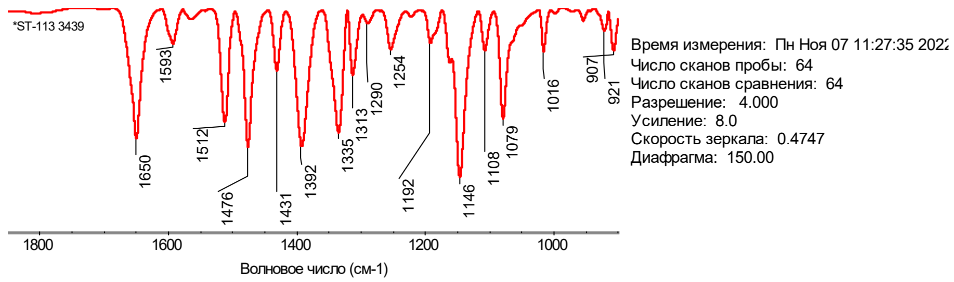

Chromatogram
ST-113 C:\LabSolutions\Data\CHEK\ST P1125-07.lcd

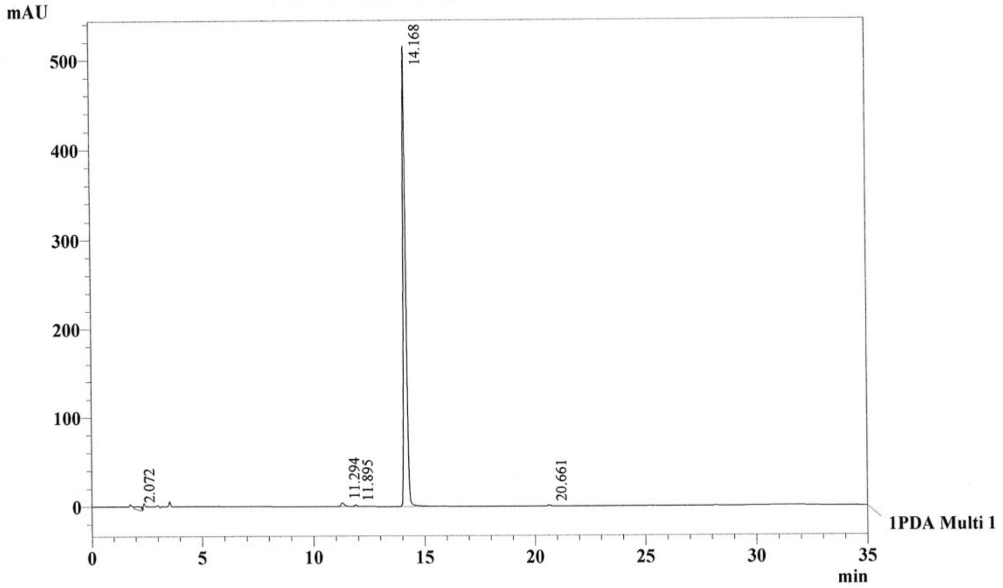

1 PDA Multi 1 / 270nm 4nm

PeakTable

PDA Ch1 270nm 4nm

Peak#	Ret. Time	Area	Height	Area %
1	2.072	64301	3435	1.427
2	11.294	39543	3815	0.878
3	11.895	13608	1563	0.302
4	14.168	4378896	515492	97.212
5	20.661	8123	1032	0.180
Total		4504471	525337	100.000

Method

<<LC Program>>

Time	Unit	Command	Value
0.10	Pumps	B.Conc	30
30.00	Pumps	B.Conc	70
33.00	Pumps	B.Conc	30
45.00	Controller	Stop	

Method Filename : FOS B.lcm

Shimadzu LC-20AD; 2-System FOS, Colon Kromasil 100-C18,.size 5mkm, 4,6*250mm, N 86912
Elution: A - H3PO4 0.01M pH 2.6; B - MeCN, fl. 1,0 ml/min, loop 20mkl.

1-isonicotinoylindoline-5-sulfonamide **(4q)**

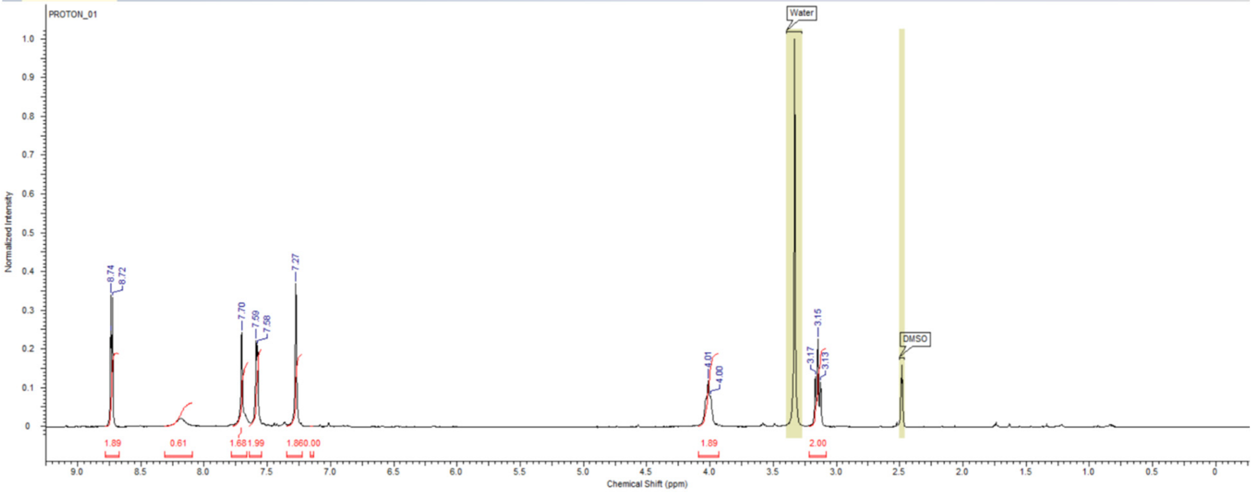

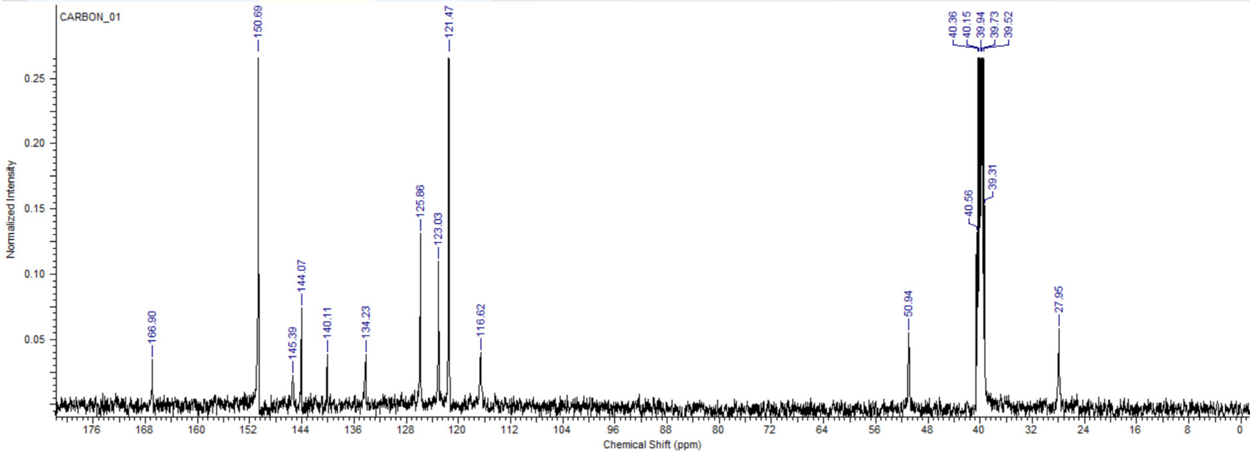

Title: *ST-111 3437 Пн Ноя 07 11:18:14 2022 (GM

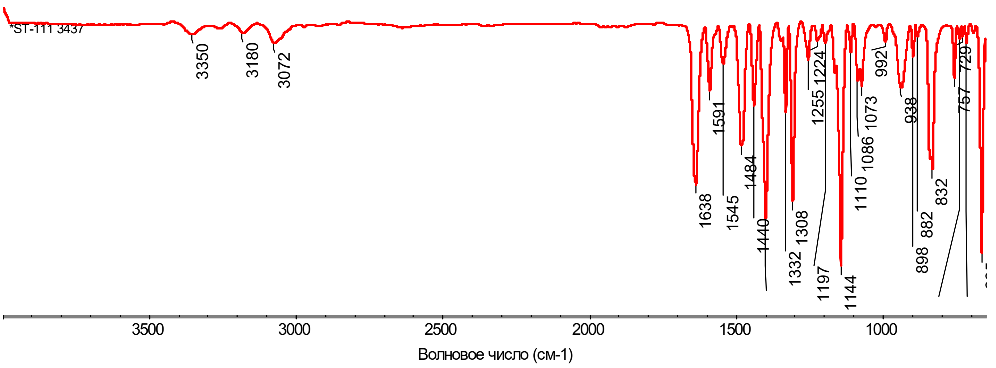

Expanded fingerprint region:

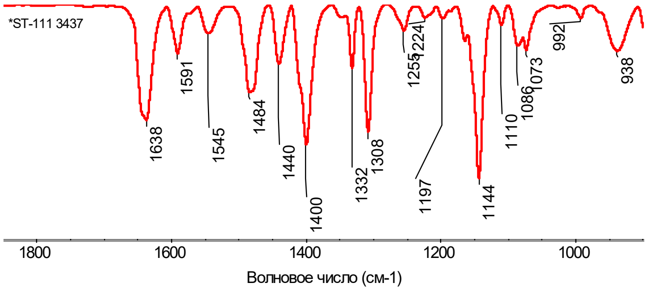

Время измерения: Пн Ноя 07 11:13:20 2022
Число сканов пробы: 64
Число сканов сравнения: 64
Разрешение: 4.000
Усиление: 8.0
Скорость зеркала: 0.4747
Диафрагма: 150.00

Chromatogram
ST-111 C:\LabSolutions\Data\CHEK\ST P1111-006.lcd

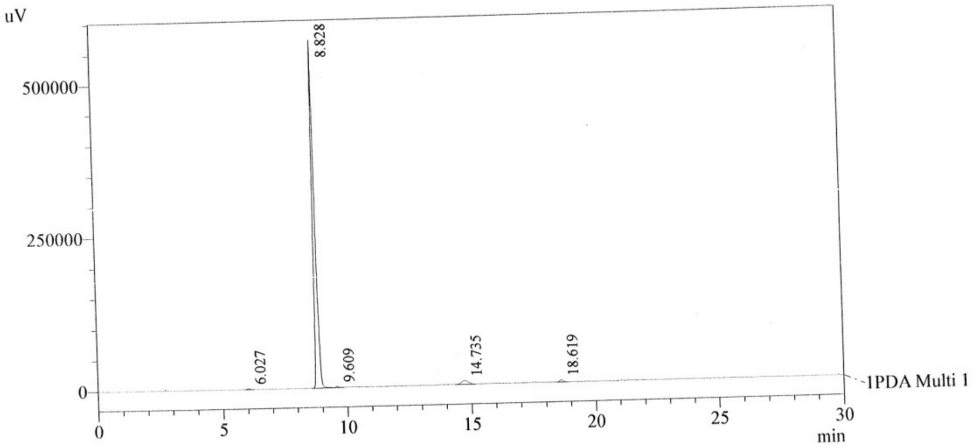

1 PDA Multi 1 / 260nm 4nm

PDA Ch1 260nm 4nm

Peak#	Ret. Time	Area	Height	Area %
1	6.027	12194	1616	0.290
2	8.828	4009315	569775	95.491
3	9.609	15424	1983	0.367
4	14.735	124637	6441	2.969
5	18.619	37066	3580	0.883
Total		4198636	583394	100.000

Method Filename : FOS B.lcm 12.11.2020 12:18:15

Time	Unit	Command	Valu
0.01	Pumps	B.Conc	10
30.00	Pumps	B.Conc	50
33.00	Pumps	B.Conc	10
45.00	Controller	Stop	

Shimadzu LC-20 AD; System - FOS Colon- Kromasil-100-5mkm. C-18, 4,6x250 mm. N 62511
Elution: A - H3PO4 0,01M pH 2,6; B - MeCN, fl - 1.0 ml/min, loop 20 mkl

1-(thiophene-2-carbonyl)indoline-5-sulfonamide **(4r)**

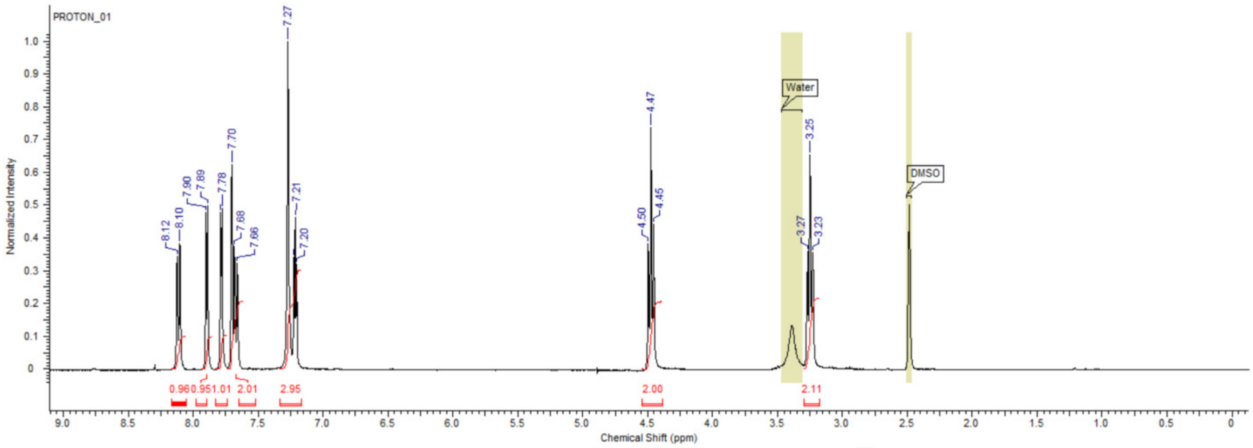

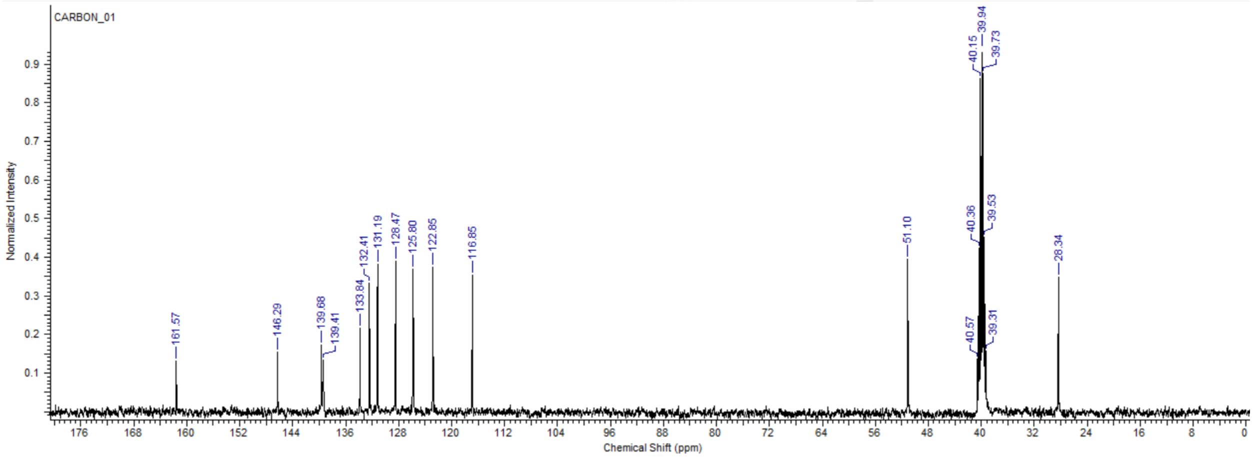

Chromatogram
ST-99 C:\LabSolutions\Data\CHEK\ST P1002-008.lcd

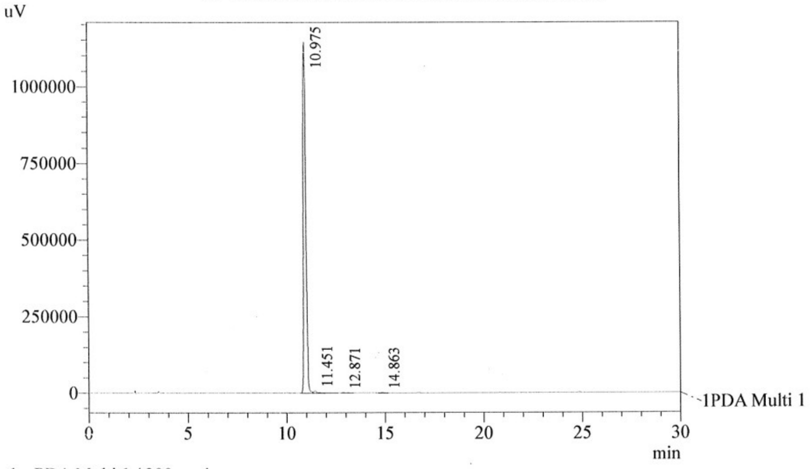

1 PDA Multi 1 / 300nm 4nm

PDA Ch1 300nm 4nm

Peak#	Ret. Time	Area	Height	Area %
1	10.975	11010663	1143769	98.850
2	11.451	94362	7117	0.847
3	12.871	18831	1116	0.169
4	14.863	14933	1719	0.134
Total		11138790	1153721	100.000

Method Filename : FOS B.lcm 05.10.2020 15:33:15

Time	Unit	Command	Valu
0.01	Pumps	B.Conc	30
30.00	Pumps	B.Conc	70
33.00	Pumps	B.Conc	30
45.00	Controller	Stop	

Shimadzu LC-20 AD; System - FOS Colon- Kromasil-100-5mkm. C-18, 4,6x250 mm. N 62511
Elution: A - H3PO4 0,01M pH 2,6; B - MeCN, fl - 1.0 ml/min, loop 20 mkl

1-(cyclopentanecarbonyl)indoline-5-sulfonamide **(4s)**

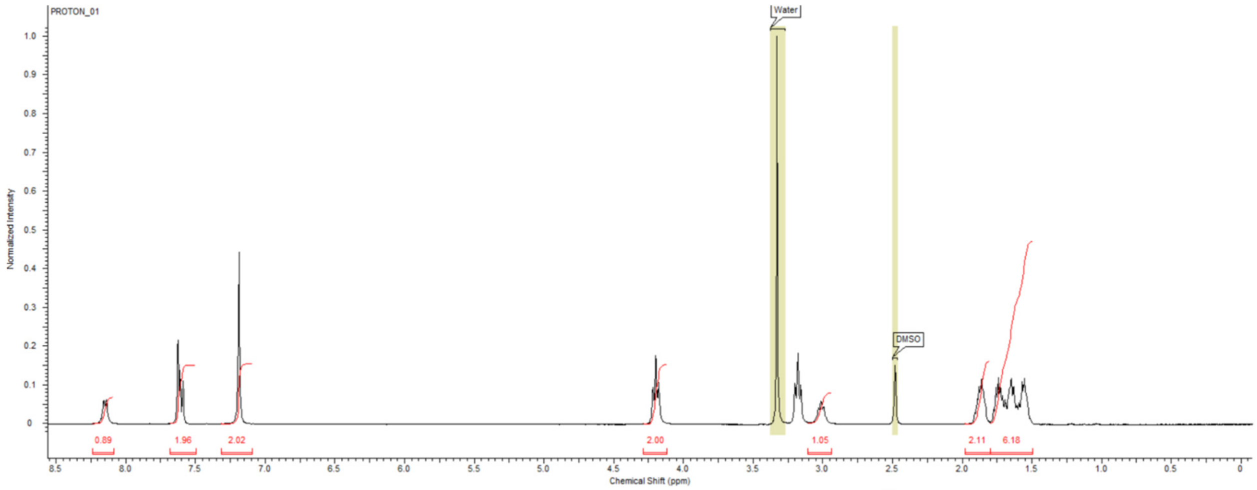

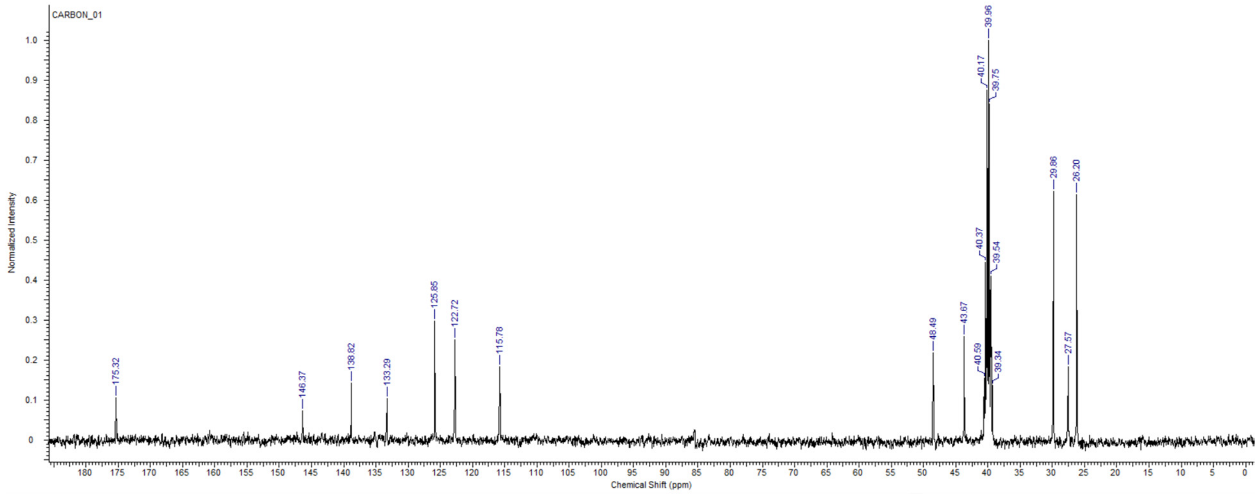

Chromatogram
ST-107 C:\LabSolutions\Data\CHEK\ST P1007-001.lcd

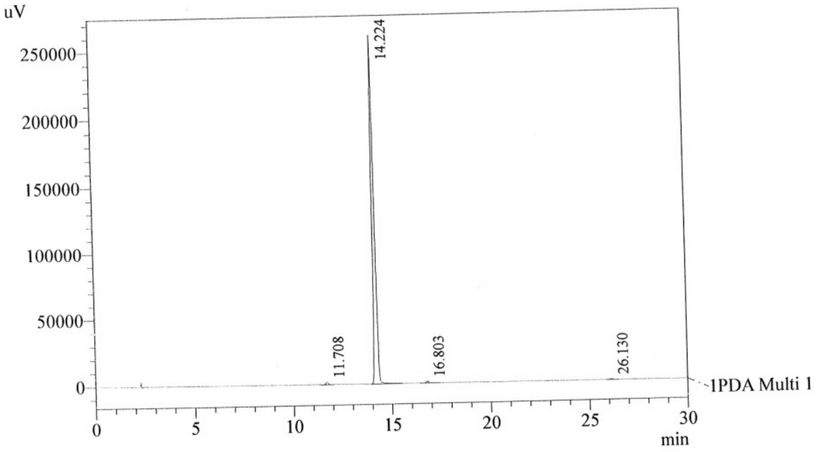

1 PDA Multi 1 / 300nm 4nm

PDA Ch1 300nm 4nm

Peak#	Ret. Time	Area	Height	Area %
1	11.708	14481	1913	0.638
2	14.224	2238145	261015	98.644
3	16.803	9786	1350	0.431
4	26.130	6491	758	0.286
Total		2268904	265037	100.000

Method Filename : FOS B.lcm 07.10.2020 14:29:08

Time	Unit	Command	Valu
0.01	Pumps	B.Conc	30
30.00	Pumps	B.Conc	70
33.00	Pumps	B.Conc	30
45.00	Controller	Stop	

Shimadzu LC-20 AD; System - FOS Colon- Kromasil-100-5mkm. C-18, 4,6x250 mm. N 62511
Elution: A - H3PO4 0,01M pH 2,6; B - MeCN, fl - 1.0 ml/min, loop 20 mkl

1-(2-cyclopentylacetyl)indoline-5-sulfonamide **(4t)**

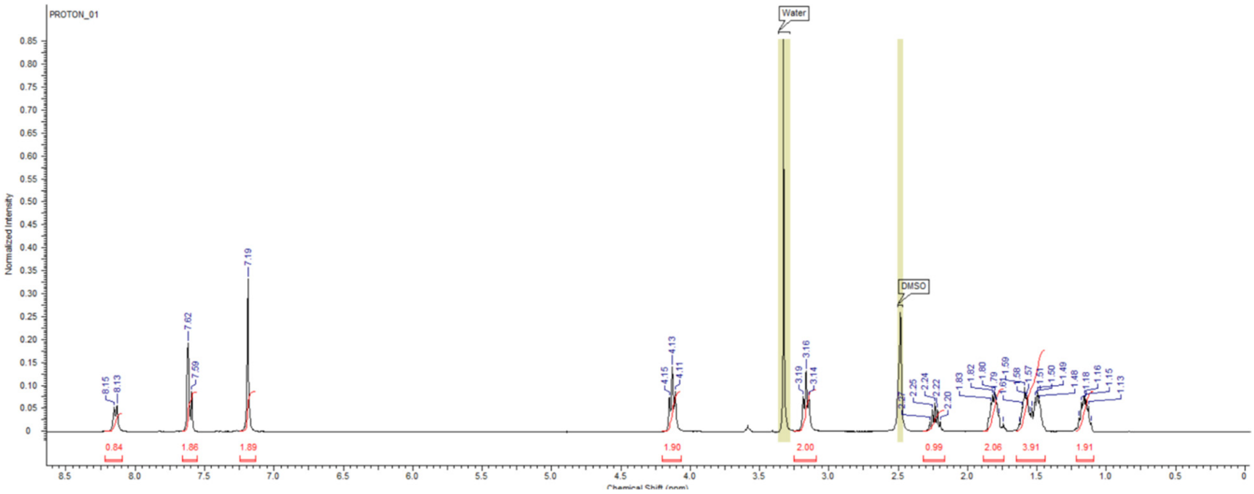

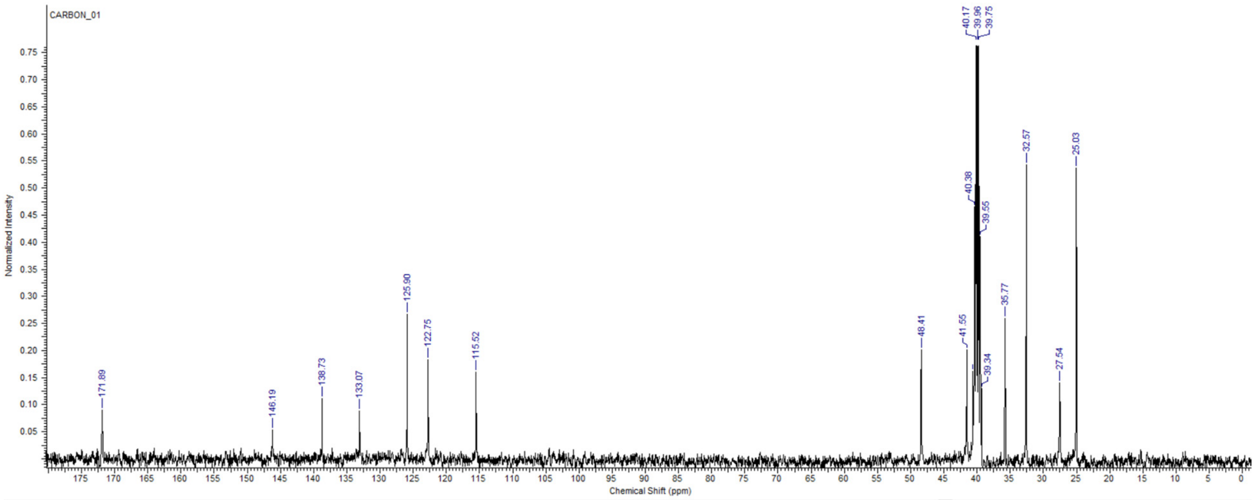

Chromatogram
ST-108 C:\LabSolutions\Data\CHEK\ST P1007-002.lcd

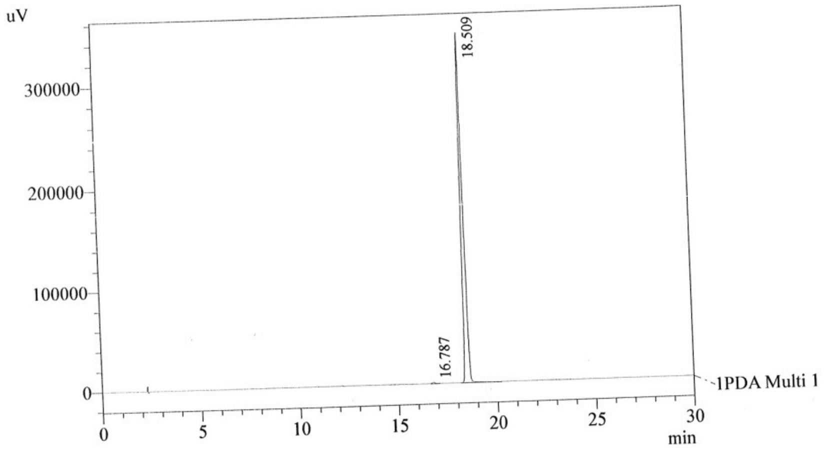

1 PDA Multi 1 / 300nm 4nm

PDA Ch1 300nm 4nm

Peak#	Ret. Time	Area	Height	Area %
1	16.787	11915	1321	0.383
2	18.509	3102682	343899	99.617
Total		3114597	345219	100.000

Method Filename : FOS B.lcm 07.10.2020 15:28:05

Time	Unit	Command	Valu
0.01	Pumps	B.Conc	30
30.00	Pumps	B.Conc	70
33.00	Pumps	B.Conc	30
45.00	Controller	Stop	

Shimadzu LC-20 AD; System - FOS Colon- Kromasil-100-5mkm. C-18, 4,6x250 mm. N 62511
Elution: A - H_3PO_4 0,01M pH 2,6; B - MeCN, fl - 1.0 ml/min, loop 20 mkl

1-(cyclohexanecarbonyl)indoline-5-sulfonamide **(4u)**

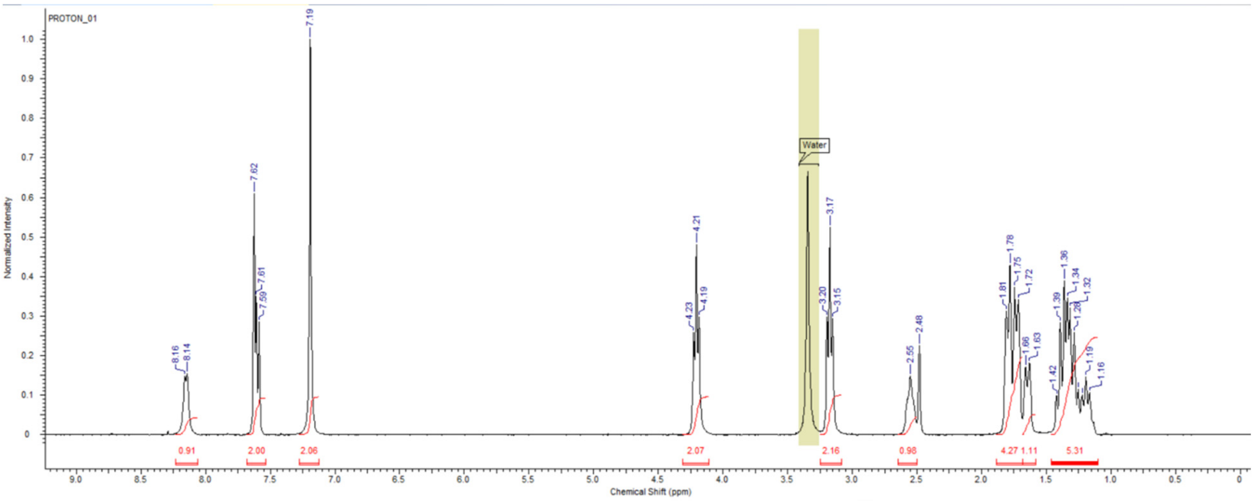

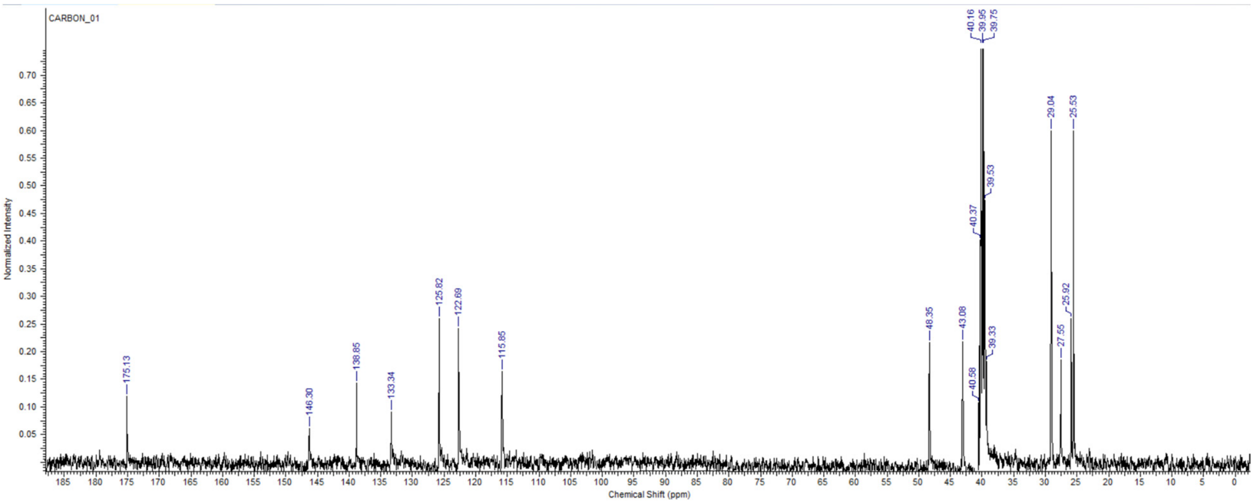

Chromatogram
ST-112 C:\LabSolutions\Data\CHEK\ST P1030-04.lcd

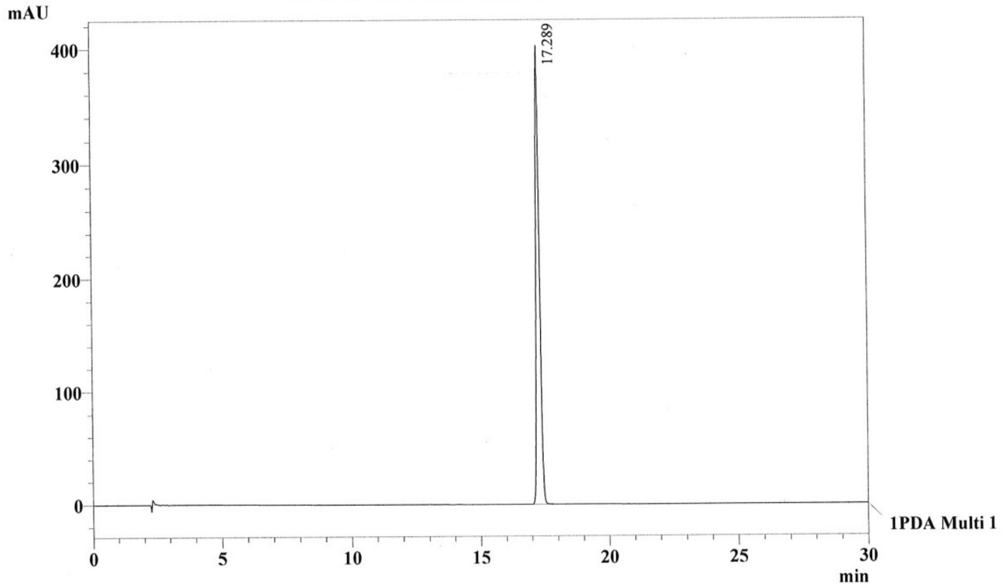

1 PDA Multi 1 / 300nm 4nm

PeakTable

PDA Ch1 300nm 4nm

Peak#	Ret. Time	Area	Height	Area %
1	17.289	3894839	402546	100.000
Total		3894839	402546	100.000

Method

<<LC Program>>

Time	Unit	Command	Value
0.10	Pumps	B.Conc	30
30.00	Pumps	B.Conc	70
33.00	Pumps	B.Conc	30
45.00	Controller	Stop	

Method Filename : FOS B.lcm

Shimadzu LC-20AD; 2-System FOS, Colon Kromasil 100-C18,.size 5mkm, 4,6*250mm, N 86912
Elution: A - H3PO4 0.01M pH 2.6; B - MeCN, fl. 1,0 ml/min, loop 20mkl.

1-benzylindoline-5-sulfonamide **(10)**

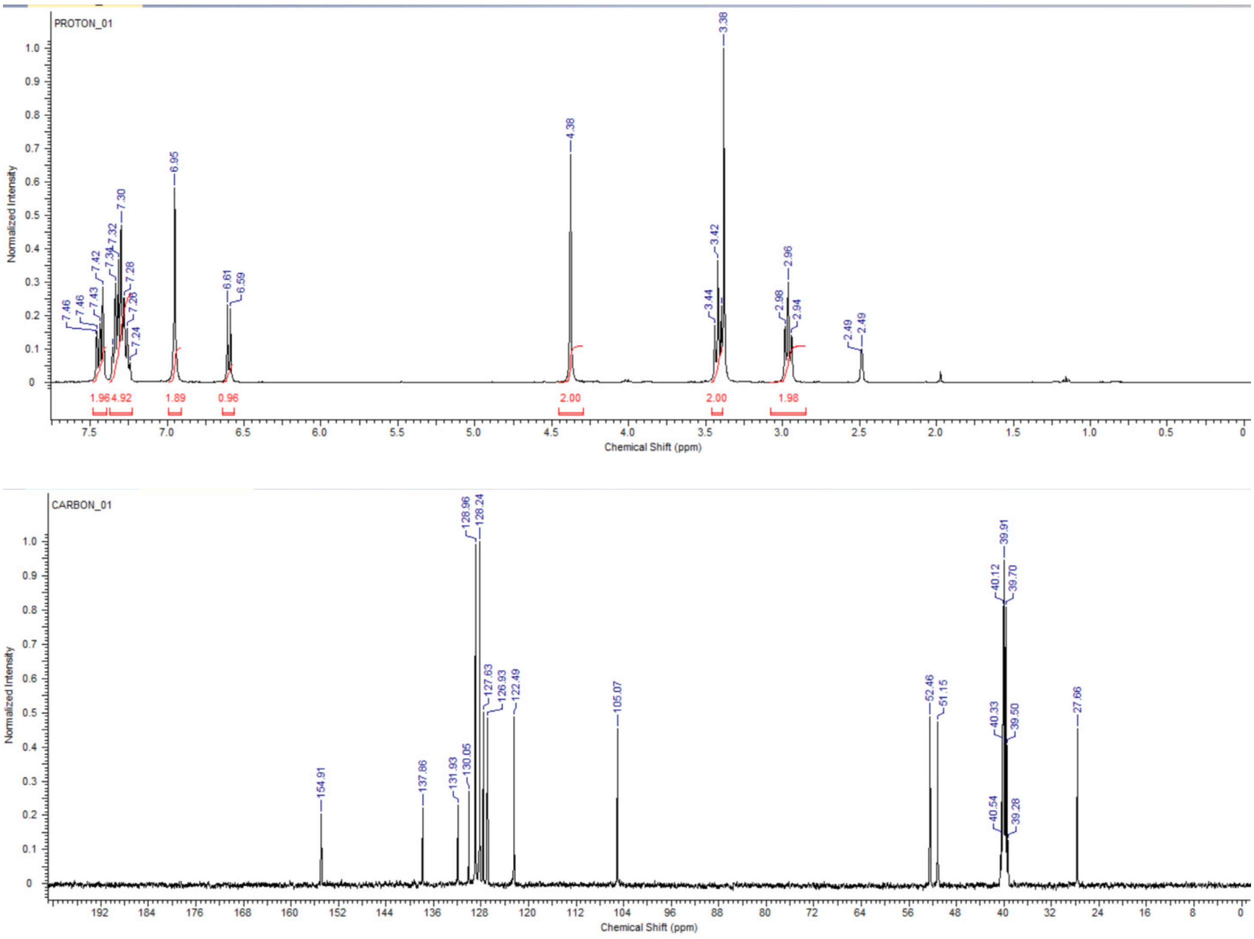

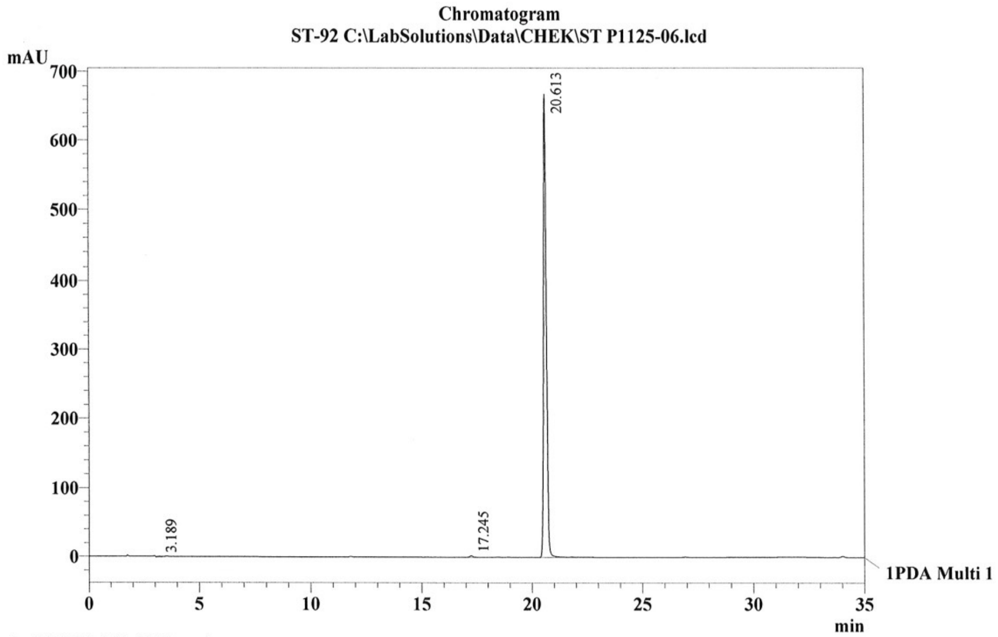

1 PDA Multi 1 / 290nm 4nm

PeakTable

PDA Ch1 290nm 4nm

Peak#	Ret. Time	Area	Height	Area %
1	3.189	11981	664	0.198
2	17.245	19525	2087	0.323
3	20.613	6005808	668401	99.478
Total		6037313	671152	100.000

Method

<<LC Program>>

Time	Unit	Command	Value
0.10	Pumps	B.Conc	30
30.00	Pumps	B.Conc	70
33.00	Pumps	B.Conc	30
45.00	Controller	Stop	

Method Filename : FOS B.lcm

Shimadzu LC-20AD; 2-System FOS, Colon Kromasil 100-C18,.size 5mkm, 4,6*250mm, N 86912
Elution: A - H_3PO_4 0.01M pH 2.6; B - MeCN, fl. 1,0 ml/min, loop 20mkl.
